# Influence of substituting 25% alfalfa hay with *Panicum maximum cv. Mombasa* with or without spirulina supplementation on the productive performance of fattening Barki lambs

**DOI:** 10.1038/s41598-025-28525-1

**Published:** 2026-01-10

**Authors:** Mohamed Ibrahim Meteab, Mahmoud Mohamed Khorshed, Abeer Mohamed EL-Essawy, Mahmoud Saber Nassar, Nasr El Sayed El-Bordeny

**Affiliations:** 1 Animal and Poultry Nutrition Department, Animal and Poultry Division, Desert Research Center, Mataryia, Cairo Egypt; 2 Animal Production Department, Faculty of Agriculture, Ain-Shams University, 68 Hadayek Shoubra 111241, Cairo, Egypt

**Keywords:** Fattening lambs, Guinea grass, Algae supplementation, Productive performance, Rumen fermentation, Biochemistry, Zoology, Animal behaviour, Animal physiology

## Abstract

Combining salt-tolerant forages with conventional forage can provide an alternative solution for enhancing livestock production in areas affected by salt. This study aimed to evaluate the effects of substituting 25% of alfalfa hay with Panicum maximum hay, with or without Spirulina (2 mg/g), on the feed intake, digestibility, ruminal fermentation, blood parameters, and growth rate of Barki lambs. Thirty-two lambs, aged 3 months, with an average initial body weight of 21.62 kg (± 3.23), were used in the fattening experiment. The animals were weighed and then randomly assigned to four groups, eight lambs each. The experimental diets were isonitrogenous and isocaloric, containing 40% forage. Alfalfa was partially replaced with Panicum hay, with T1 and T2 having 0% Panicum and T3 and T4 containing 25%. Additionally, animals in T2 and T4 received Spirulina supplementation at 2 mg/g. The substitution of 25% of alfalfa hay with Panicum resulted in a significant reduction (*p* < 0.0001) in feed intake, TVFA, and NH_3_ levels. Lambs-fed diets containing 40% alfalfa hay demonstrated the highest nutrient digestibility (CP 790.2 vs. 765.85, EE 817.7 vs. 758.6, CF 632.90 vs. 551.60), nutritive value (TDN 741.90 vs. 711.70), blood parameters (TP 6.44 vs. 5.97, Alb 3.5 vs. 3.27), and growth rates (FBW 49.75 vs. 46.06, TG 28.12 vs. 24.44, ADG 234.37 vs. 203.) compared to those receiving diets with 10% Panicum. Supplementation with Spirulina at 2 mg/g significantly increased feed intake, TVFA (7.87 vs. 6.84), NH3(18.10 vs. 16.78) levels in the rumen, digestibility (CP 746.38 vs. 710.61, EE 812.4 vs. 763.9, CF 622.10 vs. 562.40), nutritive value (TDN 736.50 vs. 717.10), blood parameters (TP 6.34 vs. 6.07, Alb 3.49 vs. 3.32), and growth rates (FBW 49.16 vs. 46.65, TG 27.53 vs. 25.03, ADG 229.47 vs. 208.59)(*p* < 0.0001). In conclusion, incorporating 25% Panicum hay in lamb diets, particularly with Spirulina supplementation (2 mg/g), supports notable performance compared to pure alfalfa diets. While alfalfa remains superior, Panicum cultivation in saline lands is still viable. This combination is especially beneficial for lamb production in Egypt’s desert regions.

**Supplementary Information:**

The online version contains supplementary material available at 10.1038/s41598-025-28525-1.

## Introduction

 Egypt faces the prospect of severe water scarcity due to population growth and climate change^1^. More than 97% of the country’s renewable water resources come from outside its borders, while the annual rainfall is only about 1.3 billion cubic meters, accounting for just 2.3% of Egypt’s total water supply^2^. This results in a significant shortfall in the production of both green and dry animal forage, which in turn affects the production of red meat and hinders the ability to meet local consumption needs. To address this issue, it is necessary to increase the area planted with feed crops in both old and new lands^3^. To enhance livestock productivity in Egypt, a key strategy is to cultivate fodder crops that can tolerate water shortages and high salinity, particularly in desert regions. One notable option is *Panicum maximum*, commonly known as Guinea grass. This plant is native to Africa but has been introduced to nearly all tropical areas as a source of animal feed. *Panicum maximum*, a perennial herb belonging to the Poaceae family, is known for its resilience in high salinity conditions and limited water availability^4^. Umar Saleh^5^ reported that the inclusion of 15–60% in *Panicum maximum cv. Mombasa* into the diet of Red Sokoto bucks increased the diet’s digestibility from an average of 62.27 to 74.99%, and improved Nitrogen retention from 4.65 to 5.77 g/day. Similarly, Oyaniran et al.^6^ found that the West African Dwarf (WAD) rams fed legume pellets supplemented with *Panicum maximum* had improved digestibility compared to those fed solely *Panicum maximum.* In an in vitro study (preliminary study), Meteab et al.^7^ demonstrated that replacing 25% of alfalfa hay with *Panicum maximum* hay, supplemented with 2 mg/g of spirulina, resulted in the highest rates of Dry Matter (DM) and Organic Matter (OM) degradation, gas production, and fermentation parameters compared to high replacement levels. These findings highlight the importance of exploring natural feed additives in ruminant nutrition. Microalgae such as Spirulina, Chlorella, and Dunaliella can be feed additives^8^. Among these, spirulina is considered a suitable candidate due to its rich composition of micro- and macronutrients^9^. Spirulina is a valuable source of proteins, vitamins, minerals, β-carotene, and fatty acids^10^ and has been widely utilized in animal nutrition, particularly for ruminants, due to it containing a heap of biologically active substances^11^. Additionally, *Spirulina* contains a range of biologically active compounds that contribute to its antioxidant properties, hypolipidemic effects, and anti-inflammatory benefits^12^. It also helps protect cells from oxidative stress by delaying or inhibiting lipid oxidation^13^. Therefore, this study aimed to evaluate the effect of replacing 25% alfalfa with *Panicum maximum* hay with or without 2 mg/g of Spirulina supplementation on feed intake, digestibility, rumen fermentation, nitrogen balance, blood parameters, and body weight in Barki lambs.

## Materials and methods

### Study location and experimental materials

This research was conducted in Maryout Research Station (N 30°09’07.5024”, E 31°14’28.9824”), located 200 km from Cairo in the western part of Alexandria Governorate, and Animal Nutrition Department labs, Desert Research Center, Cairo (N 30°07’18.0372”, E 31°18’54.3276”) which, both are affiliated to the Desert Research Center (DRC), Cairo, Egypt.

Animals, procedures, and protocols in this experiment were inspected and accepted by the Institutional Animal Care and Use Committee of the Animal and Poultry Production Division of the Desert Research Center, Egypt (approval No. 2023–0221).

*Panicum maximum cv. Mombasa* was harvested from Al-Maghrah (N 30°15’10.9944”, E 28°55’55.0056”), Matrouh Governorate, while Alfalfa hay (*Medicago sativa*) was harvested from Maryout Research Station. Both plants (Alfalfa hay and *Panicum maximum* hay) were sun-dried (temperature more than 20 °C and humidity 33%) for six days until the hays were obtained. The hays, Alfalfa hay (*Medicago sativa*), and *Panicum maximum* were chopped, dried, and stored until used in the feeding experiment. Spirulina powder (100% purity) was obtained from the National Research Centre, Egypt.

### Animals, feeding, and experimental design

A 120-day fattening trial was conducted with thirty-two Barki lambs (3 months old; initial body weight: 21.62 ± 3.23 kg) to evaluate the effects of forage replacement and Spirulina supplementation on growth performance and nutrient utilization. The experiment followed a factorial design with 2 treatment diets (0% vs. 25% *Panicum maximum* hay, replacing alfalfa hay) and 2 levels of spirulina supplementation (0 mg/g vs. 2 mg/g of feed).

This design produced four treatment combinations as follows:

T1 (Control): 0% *Panicum maximum* (40% alfalfa hay) + no Supplementation.

T2: 0% *Panicum maximum* (40% alfalfa hay) + 2 mg/g Spirulina.

T3: 25% *Panicum maximum* (30% alfalfa hay + 10% *Panicum maximum* hay) + no Supplementation.

T4: 25% *Panicum maximum* (30% alfalfa hay + 10% *Panicum maximum* hay) + 2 mg/g Spirulina.

The lambs were weighed and randomly allocated into four equal groups (*n* = 8 per group), with each group assigned to one of the experimental diets. All animals were housed under uniform management and environmental conditions throughout the experimental period. The experimental diets were formulated to be iso-caloric and iso-nitrogenous, consisting of 40% forage and 60% concentrate feed mixture (CFM). The selected forage replacement ratio and Spirulina level were based on preliminary in vitro studies conducted by Meteab et al., ^7^ which demonstrated that replacing 25% of alfalfa hay with *Panicum maximum* hay, combined with 2 mg/g Spirulina supplementation, resulted in optimal dry matter and organic matter degradability, gas production, and ruminal fermentation. These findings provided a strong rationale for adopting these levels in the current study. The experimental design, diet formulation, and chemical compositions are detailed in Table 1.

**Table 1:** Experimental design, ingredients of the basal diet/100 kg DM, and chemical composition of the experimental dietary formulations used in the fattening trial.

		T1	T2	T3	T4	Alfalfa	Panicum	Spirulina
		Experimental design						
Factor (1): 40% forage	Alfalfa hay	100%	100%	75%	75%	-	-	-
	Panicum hay	0%	0%	25%	25%	-	-	-
Factor (2): spirulina		0 mg/g	2 mg/g	0 mg/g	2 mg/g		-	-
60% concentrate feed mixture (CFM)		CFM1	CFM1	CFM2	CFM2	-	-	-
Number of lambs		8	8	8	8	-	-	-
Duration of the experiment (days)		120	120	120	120	-	-	-
Ingredients of concentrate feed mixture (CFM)								
Alfalfa hay (A), kg/100kg		40	40	30	30	-	-	-
* Panicum maximum* (P), kg/100kg		0.00	0.00	10	10	-	-	-
Corn, kg/100kg		32.8	32.8	30.9	30.9	-	-	-
Soya meal, kg/100kg		9.5	9.5	15.3	15.3	-	-	-
Wheat bran, kg/100kg		15.6	15.6	11.7	11.7	-	-	-
Salt, kg/100kg		0.9	0.9	0.9	0.9	-	-	-
Limestone, kg/100kg		0.9	0.9	0.9	0.9	-	-	-
Premix, kg/100kg		0.3	0.3	0.3	0.3	-	-	-
Chemical composition of experimental dietary formulations used in the fattening trial (g/kg DM basis)								
Ash, g/Kg		88	88	89	89	113	139	131
Dry matter, g/Kg		886	886	888	888	884	895	914
Organic matter, g/Kg		916	916	914	921	887	907	869
Crude fiber, g/Kg		154	154	157	156	333	353	60
Crude protein, g/Kg		168	168	167	167	169	80	472
Ether extract, g/Kg		26	26	28	28	5	11	38
Nitrogen-free extract, g/Kg		568	568	563	562	380	463	299
Neutral detergent fiber, g/Kg		335	335	338	338	474	546	398
Acid detergent fiber, g/Kg		151	151	153	153	311	352	189
Non-fiber carbohydrate, g/Kg		387	386	381	380	239	270	-
Calculated energy contents								
Gross energy, kcal/kg DM		3802	3802	3802	3801	3574.25	3685.75	3675.50
Digestible energy, kcal/kg DM		2501	2501	2501	2501	2351.48	2424.84	2418.09
Metabolizable energy, kcal/kg DM		2051	2051	2051	2051	1928.21	1988.37	1982.84

*T1 (Control)* CFM1 + 0% *Panicum maximum* (40% alfalfa hay) + no Supplementation, *T2* CFM1 + 0% *Panicum maximum* (40% alfalfa hay) + 2 mg/g Spirulina, *T3* CFM2 + 25% *Panicum maximum* (30% alfalfa hay + 10% *Panicum maximum* hay) + no Supplementation and *T4* CFM2 + 25% *Panicum maximum* (30% alfalfa hay + 10% *Panicum maximum* hay) + 2 mg/g Spirulina. DE, ME, and GE are calculated according to^77^.

### Digestibility trials

The digestibility trial began 90 days after the start of the experiment, using six lambs randomly selected from each treatment group. Animals were housed individually in steel metabolic cages (1.5 m height and 70 cm width). These cages facilitate urine and feces collection. The trial consisted of a 21-day adaptation period, during which lambs were fed their respective experimental diet without data collection to ensure physiological stabilization, followed by a 7-day collection period. Animals were fed twice daily at 7:00 a.m. and 2:00 p.m., with free access to water. Body weights were recorded on days 0 and 28 after a 12-hour fast. The animals were kept under routine veterinary care throughout the digestibility trial. During the collection period, the voluntary intake of each lamb was recorded, and total feces were collected and measured daily, then mixed, and sampled (10% of total feces). Urine was collected and measured daily, and 10% of the urine sample was preserved for analysis. The feed, refusals, and feces were dried at 65 °C for constant weight, ground to pass through a 1.0 mm sieve, and kept for proximate analysis. Urine samples were stored in tight bottles containing 10% sulfuric acid to capture NH_3_ and refrigerated at 4 °C for nitrogen determination.

### Rumen liquor sampling and analysis

At the end of the 7-day collection period of the digestibility trial, samples of rumen liquor were collected at 0 (pre-feeding), 3, and 6 h post-feeding using a stomach tube. Samples were filtered through four layers of cheesecloth, and the pH value was measured immediately using Orian 680 digital pH meter. Total volatile fatty acid (TVFA) concentration was determined by the steam distillation method using the Markham micro distillation unit Warner^14^, and ammonia (NH_3_) concentration was determined by Nessler’s method modified by Szumacher-Strabel^15^.

### Blood sampling and analysis

Samples of blood were taken pre-feeding at the end of the 7-day collection period of the digestibility trial. Samples were withdrawn from the jugular vein of rams using a sterile needle into clean, dry serum tubes and centrifuged at 4000 rpm for 15 min to collect serum. The serum was stored at −20 °C in clean, dry glass vials until subsequent analyses. The serum samples were analyzed using commercial kits (SPINREACT, A. A. Ctra. Santa Coloma, Girona, Spain). Total protein (TP), albumin (Alb), urea and creatinine (creat), alanine aminotransferase (ALT) and aspartate aminotransferase (AST), Cholesterol (chol) and Triglycerides (TG). Globulin (Glo) concentration was calculated as the difference between TP and Alb.

### Chemical analysis

Representative samples of feeds, feed orts, and feces were oven-dried at 65 °C for 72 h and ground in a cutter-type mill with a 1 mm sieve. The samples were then subjected to proximate analyses, including total nitrogen, crude fiber (CF), ether extract (EE), and ash according to^16^ guidelines. Crude protein (CP) was calculated by multiplying total nitrogen × 6.25, and nitrogen-free extract (NFE) was calculated by difference. Total nitrogen in urine was determined using the micro-Kjeldahl method according to AOAC^16^. Neutral detergent fiber (NDF), acid detergent fiber (ADF), and acid detergent lignin (ADL) were determined according to sequential procedures outlined by Van Soest et al.^17^, using the Ankom200 (Ankom Technology Corp., Fairport, NY) filter bag technique. Phytochemical screening for total tannins, saponins, and total phenols as the major Anti-Nutritional Factors (ANF) in all feed ingredients was carried out using the alcoholic extracts of alfalfa hay, *Panicum maximum* hay, and Spirulina extract by^18,19^ (Table 2)

**Table 2:** Preliminary phytochemical screening and quantitative estimation of the experimental ingredients’ anti-nutritional factors (ANF).

Type	Total tanninsmg/100 g	Saponins g/100gDM	Total phenol %
Alfalfa hay	++	++	++
*Panicum maximum* hay	+++	ND	+++
*Spirulina*	+	+++	+
Concentrations of the anti-nutritional factors (ANF) in the experimental ingredients			
Alfalfa hay	2.58	2.64	0.20
* Panicum maximum* hay	3.75	ND	0.25
* Spirulina*	1.09	3.66	0.17

*+* present, *ND* Not Detected.

### Statistical analysis

The experiment followed a factorial design (2 treatment diets × 2 spirulina supplementation levels) in a completely randomized design. Data were statistically analyzed using SAS software^20^. Separation among means was carried out according to Duncan’s multiple-range test^21^ when the main factor was significant and at a confidence level of 95%. The data on feed intake, digestibility, nitrogen balance, blood parameters, and body weight were analyzed using two-way ANOVA with an interaction analysis according to the following General Linear Model.

$$Yij=m\,+\,Ti+Sj+\left( {S \times T} \right){\text{ }}ij+eij$$

Where: Yij = The observation on the I^th^ treatment, µ = Overall mean, Ti = Effect of the I^th^ treatment diet, Sj = Effect of the supplementation, (S*T) ij = Effect of the interaction between factors I and J, and eij = Random experimental error.

The data of rumen fermentation parameters were subjected to the analysis using the MIXED procedure of SAS according to the following MIXED Model.

$$Yijkl\,=\,m\,+\,Ai\,+\,Bj+\left( {A \times B} \right)ij\,+\,Tk+\left( {A \times T} \right)ik+\left( {B \times T} \right)jk+\left( {A \times B \times T} \right)ijk\,+\,Animall\left( {ij} \right)+eijkl$$

Where: Yijkl = The K ^th^ Observation on the diet subjected to factors A and B, µ = overall mean, Ai = fixed effect of substitution level (i = 0, 25%), Bj = fixed effect of supplementation (j = 0 and 2 mg/), (A×B)ij = interaction between factors A and B, Tk = fixed effect of time (k = repeated measurement occasions), Animall(ij) = random effect of animal nested within the A×B combination (subject term for repeated measures) and, εijkl = Random experimental error.

## Results

The chemical composition data in Table 1 indicate numerical differences between alfalfa hay and *Panicum maximum* hay, particularly in CP and fiber fractions, including CF, NDF, and ADF. Alfalfa hay contained approximately 169 g/kg of CP, whereas *Panicum maximum* hay had a significantly lower CP content of around 80 g/kg. Conversely, *Panicum maximum* hay exhibited higher fiber levels, with CF, NDF, and ADF values recorded at 353 vs. 333 g/kg, 546 vs. 474 g/kg, and 352 vs. 311 g/kg, respectively, compared to alfalfa hay. These variations in chemical composition, particularly in protein content, necessitated the formulation of experimental diets to be both isonitrogenous and isocaloric.

### Feed intake

**Fig. 1:**
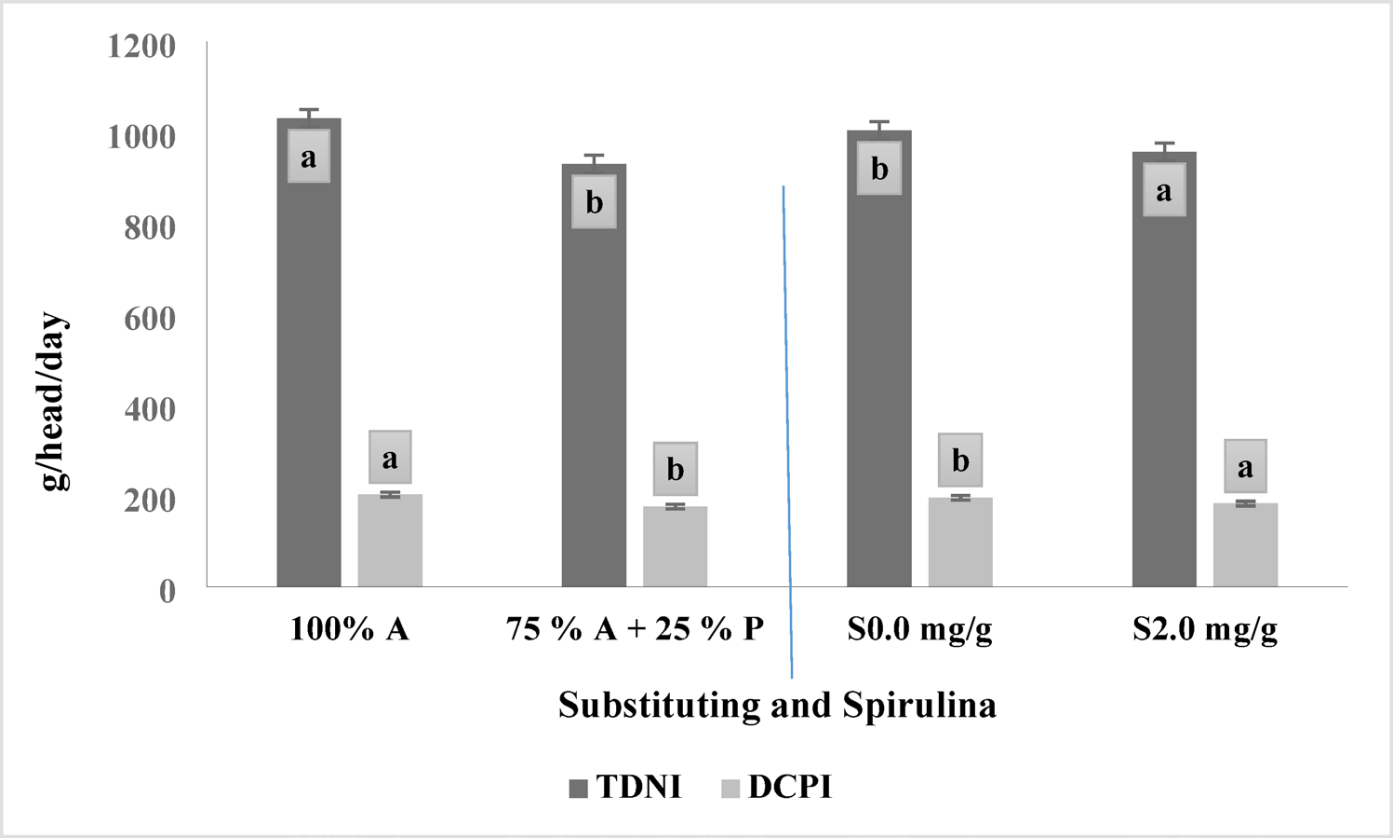
The two main effects of substituting Alfalfa hay 100% (A), Alfalfa hay 75% (A), *Panicum maximum* hay 25% (P) and additives Spirulina 2.0 mg/g (S), 0.0 mg/g on feed intake. Varied letters of columns points out that there were significant differences at 0.05 level of probability. Means were distinguished employing Duncan’s multiple range test (*p* ≤ 0.05). Digestible Crude protein intake (DCPI), DM/day, Total Digestible Nutrient Intake (TDNI), DM/day.

Table 3 shows the effect of substituting alfalfa hay with *Panicum maximum* with or without adding Spirulina (2 mg/g) and their interaction on feed intake during the fattening experiment in Barki lambs. Substituting 25% of alfalfa hay with *Panicum maximum* shows a significant (*p* < 0.005) reduction in Total Digestible Nutrients intake (TDNI) (g/h/day and g/kgBW^0.75^), and Digestible Crude protein intake (DCPI) (g/h/day and g/kgBW^0.75^), Fig. 1, regardless of Spirulina supplementation.

**Table 3:** Effect of substituting alfalfa hay with *Panicum maximum* hay with or without spirulina addition on feed intake during the fattening trial.

Substituting	100% A		75% A + 25% *P*		Standard error	*P* value		
Spirulina	0.0 mg/g	2.0 mg/g	0.0 mg/g	2.0 mg/g				
TRT	T1	T2	T3	T4		Substituting	Spirulina	Interaction
Body weight								
ABW. Kg	35.0	36.4	33.3	34.4	1.18	0.1288	0.2958	0.9182
MBW (BW^0.75)^	14.4	14.8	13.9	14.2	0.36	0.1303	0.2924	0.9056
Concentrate intake, DM/day								
g/h/day	828.9	851.9	775.0	796.2	71.44	0.4501	0.7896	0.991
g/kgBW^0.75^	58.1	57.8	56.1	56.3	1.40	0.2113	0.9859	0.8551
Forage intake, DM/day								
g/h/day	550.9	564.2	515.9	528.2	47.51	0.4607	0.7896	0.9912
g/kgBW^0.75^	38.6	38.3	37.3	37.3	0.93	0.2353	0.8749	0.8540
Total dry matter Intake (TDMI), DM/day								
g/h/day	1379.8	1416.1	1290.9	1324.3	118.95	0.4543	0.7896	0.9911
g/kgBW0.75	96.7	96.1	93.4	93.6	2.34	0.2208	0.9402	0.8551
Total digestible nutrient intake (TDNI), DM/day								
g/h/day	1012.5	1050.7	904.3	957.5	4.65	< 0.0001	< 0.0001	0.0961
g/kgBW^0.75^	71.0	71.3	65.4	67.7	1.69	0.0115	0.4542	0.5579
Crude protein intake (CPI), DM/day								
g/day	231.2	238.2	215.2	221.6	19.89	0.4194	0.7896	0.9905
g/kgBW^0.75^	16.2	16.2	15.6	15.7	0.39	0.1537	0.9382	0.8557
Digestible crude protein intake (DCPI), DM/day								
g/day	196.8	209.1	170.0	183.9	0.54	< 0.0001	< 0.0001	0.1772
g/kgBW^0.75^	13.8	14.2	12.3	13.0	0.33	0.0004	0.1109	0.6482

*A* alfalfa hay, *P Panicum maximum* hay. *ABW* Average Body weight, *MBW* Metabolic body weight W^0.75^. * Average Body weight was calculated as the average of weights over 120 days. *T1 (Control)* 0% *Panicum maximum* (40% alfalfa hay) + no Supplementation, *T2* 0% *Panicum maximum* (40% alfalfa hay) + 2 mg/g Spirulina, *T3* 25% *Panicum maximum* (30% alfalfa hay + 10% *Panicum maximum* hay) + no Supplementation and *T4* 25% *Panicum maximum* (30% alfalfa hay + 10% *Panicum maximum* hay) + 2 mg/g Spirulina.

However, across both substitution levels, the animals that received diets supplemented with 2 mg/g Spirulina had significantly higher (*p* < 0.005) TDNI (g/day) and DCPI (g/day) Fig. 1 than those that received non-supplemented diets. The interaction between forage substitution levels and Spirulina supplementation was not statistically significant for Total Dry Matter Intake (TDMI) (g/h/day and g/kgBW^0.75^), TDNI (g/h/day and g/kgBW^0.75^), and DCPI (g/h/day and g/kgBW^0.75^).

### Rumen fermentation parameters

The impact of substituting 25% of Alfalfa hay with *Panicum maximum* hay, with or without Spirulina supplementation at 2 mg/g of feed, on ruminal fermentation parameters such as NH_3_, TVFA, and pH value concentrations were presented in Table 4. All rumen parameters remained within the normal ranges for healthy animals. The data showed that sampling time (0, 3, and 6 h post-feeding) significantly affected the rumen TVFA and NH_3_ concentrations, following a normal fermentation pattern. The highest values (*p* < 0.05) were recorded at 3 h post-feeding, followed by a gradual decline, reaching the lowest concentration at 0 h (pre-feeding), reflecting normal rumen fermentation dynamics.

**Table 4:** Effect of substituting alfalfa hay with *Panicum maximum* hay with or without spirulina addition on rumen fermentation parameters during the fattening trial.

Forage contents	TRT	Spirulina, mg/g	Time	TVFA, mequ/dl	NH_3_, mg/dl	pH value
100% A	T1	0.0 mg/g	Zero Hrs.	6.11^g^	16.0	6.7
			3 h.	8.83^b^	19.4	6.2
			6 h.	7.68^d^	17.8	6.3
	T2	2.0 mg/g	Zero Hrs.	7.22^ed^	17.9	6.6
			3 h.	10.00^a^	21.4	6.2
			6 h.	9.12^b^	19.1	6.3
75% A + 25% P	T3	0.0 mg/g	Zero Hrs.	5.32^h^	14.1	6.8
			3 h.	6.80^fe^	17.4	6.3
			6 h.	6.33^fg^	15.9	6.6
	T4	2.0 mg/g	Zero Hrs.	5.60^h^	14.9	6.7
			3 h.	8.30^c^	18.4	6.3
			6 h.	7.00^e^	16.8	6.4
Standard error	Interaction			0.17	0.23	0.03
*p* value	Substituting (A)			< 0.0001	< 0.0001	< 0.0001
	Spirulina (B)			< 0.0001	< 0.0001	0.0005
	A * B			0.0266	0.0122	0.2456
	Time			< 0.0001	< 0.0001	< 0.0001
	A*time			0.0348	0.4902	0.2089
	B*Time			0.0583	0.4166	0.8380
	A * B*Time			0.0526	0.4813	0.2558

*A* alfalfa hay, *P Panicum maximum* hay, *TVFA* Total Volatile Fatty Acids, *NH*_*3*_ Ammonia. *T1 (Control)* 0% *Panicum maximum* (40% alfalfa hay) + no Supplementation, *T2* 0% *Panicum maximum* (40% alfalfa hay) + 2 mg/g Spirulina, *T3* 25% *Panicum maximum* (30% alfalfa hay + 10% *Panicum maximum* hay) + no Supplementation and *T4* 25% *Panicum maximum* (30% alfalfa hay + 10% *Panicum maximum* hay) + 2 mg/g Spirulina. ^a^ and ^b^ mean that different superscripts in the same row are significantly different.

**Fig. 2:**
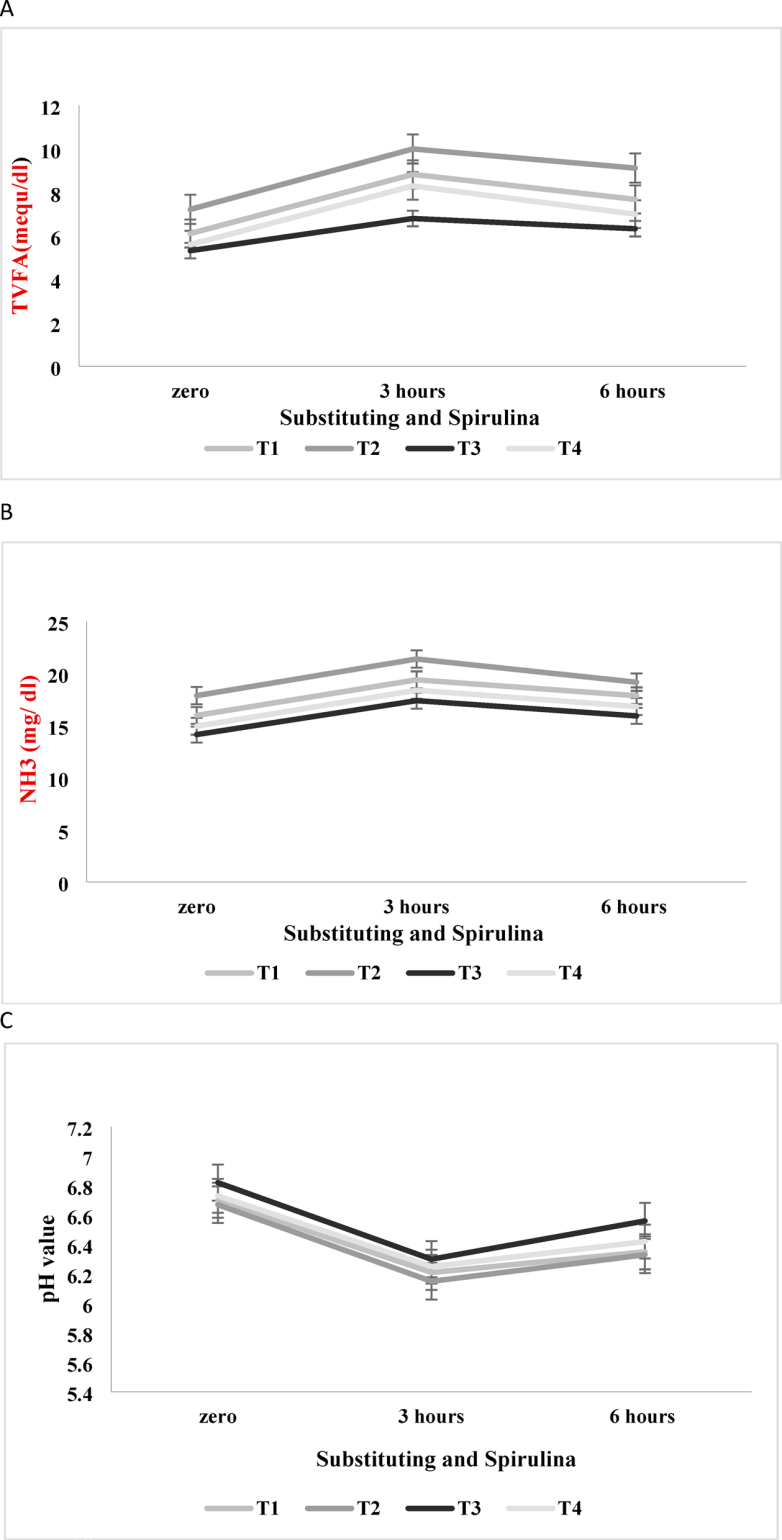
(**A**) Effect of interaction between substituting Alfalfa hay with *Panicum maximum* hay, Spirulina, and time (0–3−6 h) on TVFA. (**B**) Effect of interaction between substituting Alfalfa hay with *Panicum maximum* hay, Spirulina and time (0–3−6 h)on NH3. (**C**) Effect of interaction between substituting Alfalfa hay with *Panicum maximum* hay, Spirulina and time (0–3−6 h) on PH. T1 (Control): 0% Panicum maximum (40% alfalfa hay) + no Supplementation, T2: 0% Panicum maximum (40% alfalfa hay) + 2 mg/g Spirulina, T3: 25% Panicum maximum (30% alfalfa hay + 10% Panicum maximum hay) + no Supplementation, and T4: 25% Panicum maximum (30% alfalfa hay + 10% Panicum maximum hay) + 2 mg/g Spirulina.

The data indicated that substituting 25% of alfalfa hay with *Panicum maximum* hay significantly reduced (*p* < 0.0001) the concentrations TVFA and NH_3_ Fig. 2A,B receptivity. The highest levels of TVFA and NH_3_ were recorded for animals that fed on a diet containing 40% alfalfa hay, compared to those that received diets with 30% alfalfa hay and 10% *Panicum maximum* hay.

Supplementing the lamb’s diet with Spirulina at 2 mg/g significantly increased TVFA and NH_3_ concentrations in the rumen (*p* < 0.0001) at both substitution levels (0% and 25%), compared to animals fed on non-supplemented diets. However, the impact of Spirulina supplementation on ruminal pH was minimal.

The interaction among substituting level, Spirulina supplementation, and sampling time did not significantly affect NH_3_ concentration (*p* > 0.05). However, a significant interaction effect (*p* < 0.05) was observed for TVFA concentration. The highest TVFA was observed in lambs fed T2 (100% alfalfa hay with 2 mg/g Spirulina), followed by T1 (100% alfalfa hay without Spirulina). The lowest TVFA concentration was recorded in lambs fed T3 (25% *Panicum maximum* and 75% alfalfa hay without 2 mg/g Spirulina).

The ruminal pH value Fig. 2C was affected significantly (*p* < 0.0001) by sampling time in a non-uniform pattern where the highest pH value was at zero time(pre-feeding), followed by a decrease at three hours post-feeding, then increased again after 6 h post-feeding. Moreover, substituting 25% of alfalfa hay with *Panicum maximum* hay significantly (*p* < 0.0001) increased rumen liquor pH values. The highest pH values were recorded in animals fed 10% *Panicum maximum* hay, followed by those fed 40% alfalfa hay. Also, the data indicated that supplementing animal diets with spirulina at the rate of 2 mg/g DM decreased the pH value compared to the animals that received non-supplemented diets. However, the interaction among forage substitution, Spirulina supplementation, and sampling time did not significantly impact ruminal pH values (*p* > 0.05).

### Digestibility and nutritive value

**Fig. 3:**
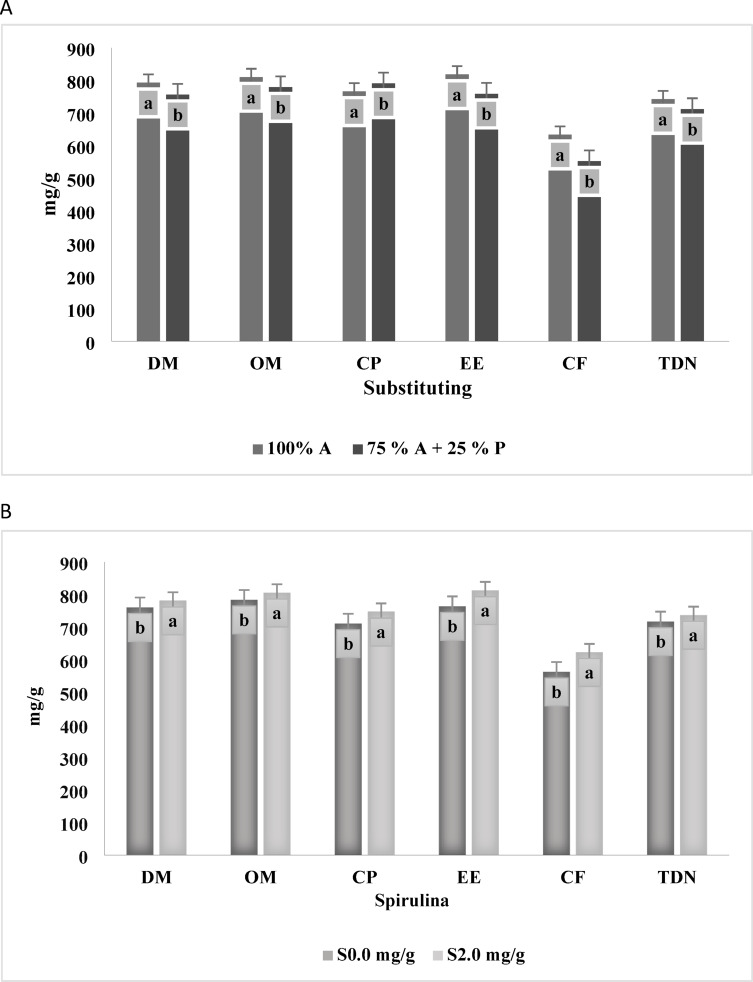
(**A**) Effect of substituting Alfalfa hay 100% (A) and Alfalfa hay 75% (A), *Panicum maximum* hay 25% (P) on digestibility. (**B**) Effect of additives Spirulina 2.0 mg/g (S) and 0.0 mg/g on digestibility. The varied letters of the columns points out that there were significant differences at a 0.05 level of probability. Means were distinguished employing Duncan’s multiple range test (*p* ≤ 0.05). *DM* Dry matter, *OM* Organic matter, *CP* Crude protein, *EE* Ether extract, *CF* Crude fiber, *TDN* Total Digestible.

The data presented in Table 5; Fig. 3A indicate that substituting 25% of alfalfa hay with *Panicum maximum* hay significantly decreases (*p* < 0.0001) in all nutrient digestibility and nutritive value parameters, including DM, OM, CP, EE, CF, and total digestible nutrients (TDN). Lambs-fed diets containing 40% alfalfa hay as forage recorded the highest nutrient digestibility and nutritive value compared to those received diets containing 10% *Panicum maximum* and 30% alfalfa hay.

**Table 5:** Effect of substituting alfalfa hay with *Panicum maximum* hay with or without spirulina addition on digestibility and nutritive value during the fattening trial.

Forage contents	TRT	Spirulina, mg/g	DM, mg/g	OM, mg/g	CP, mg/g	EE, mg/g	CF, mg/g	TDN, mg/g
100% A	T1	0.0 mg/g	781.09	800.89	765.85	789.63	609.92	733.79
	T2	2.0 mg/g	794.12	809.90	790.20	838.48	639.21	741.97
75% A + 25% P	T3	0.0 mg/g	739.86	766.46	710.61	738.34	514.91	700.52
	T4	2.0 mg/g	771.94	791.16	746.38	778.93	588.30	723.01
Standard error			5.29	5.03	2.92	7.34	10.32	4.61
*p* value		Substituting	< 0.0001	< 0.0001	< 0.0001	< 0.0001	< 0.0001	< 0.0001
		Spirulina	0.0004	0.0032	< 0.0001	< 0.0001	< 0.0001	0.0033
		Interaction	0.0867	0.1345	0.0651	0.5798	0.0451	0.1359

*A* alfalfa hay, *P Panicum maximum* hay. *T1* (Control) 0% *Panicum maximum* (40% alfalfa hay) + no Supplementation, *T2* 0% *Panicum maximum* (40% alfalfa hay) + 2 mg/g Spirulina, *T3* 25% *Panicum maximum* (30% alfalfa hay + 10% *Panicum maximum* hay) + no Supplementation, and *T4* 25% *Panicum maximum* (30% alfalfa hay + 10% *Panicum maximum* hay) + 2 mg/g Spirulina. *TRT* Treatments. *DM* Dry matter, *OM* Organic matter, *CP* Crude protein, *EE* Ether extract, *CF* Crude fiber, TDN Total Digestible.

Furthermore, the data presented in Table 5; Fig. 3B show that animals receiving diets supplemented with 2 mg/g spirulina had significantly higher (*p* < 0.0001) digestibility and nutritive value than those not receiving spirulina supplementation. However, the interaction between substituting alfalfa hay with *Panicum maximum* hay and spirulina supplementation (at 0 and 2 mg/g) did not significantly affect any digestibility parameters or nutritive values (*p* > 0.05).

### Nitrogen balance

**Fig. 4:**
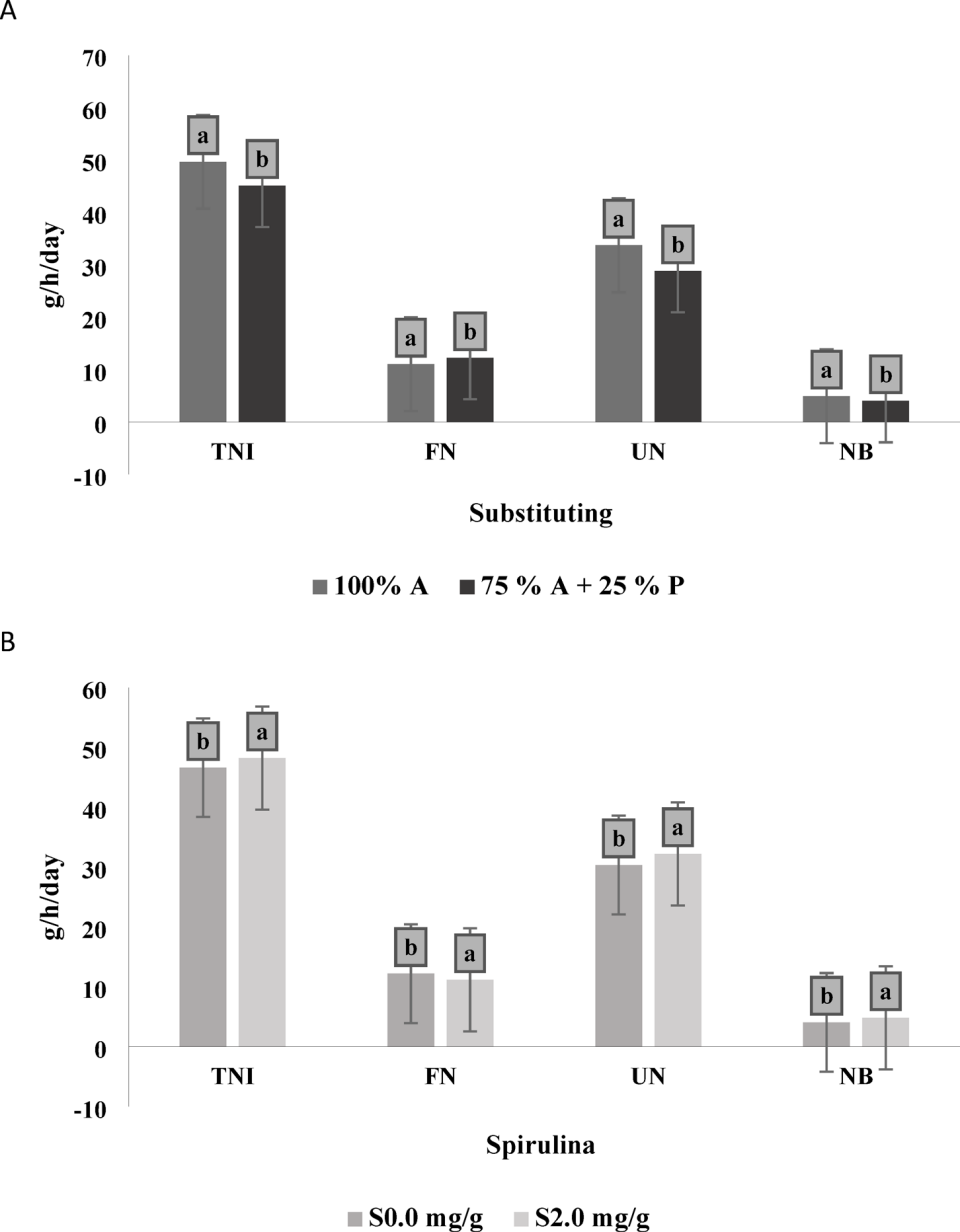
(**A**) Effect of substituting Alfalfa hay 100% (A) and Alfalfa hay 75% (A) *Panicum maximum* hay 25% (P) on nitrogen balance. (**B**) Effect of additives Spirulina 2.0 mg/g (S) and 0.0 mg/g on nitrogen balance. Varied letters of columns points out that there were significant differences at 0.05 level of probability. Means were distinguished employing Duncan’s multiple range test (*p* ≤ 0.05). Nitrogen intake (TNI), Fecal nitrogen (FN), Urinary nitrogen (UN), Total nitrogen excretion (TNE), and Nitrogen balance (NB).

The data presented in Table 6; Fig. 4A indicate that substituting 25% of alfalfa hay with *Panicum maximum* hay resulted in a significant reduction (*P* < 0.0001) in fecal nitrogen (FN) while significantly increasing (*p* < 0.0001) in urinary nitrogen (UN), total nitrogen intake (TNI), total nitrogen excretion (TNE), and nitrogen balance (NB).

**Table 6:** Effect of substituting alfalfa hay with *Panicum maximum* hay with or without spirulina addition on nitrogen balance (g/h/day) during the fattening trial.

Forage contents	TRT	Spirulina	TNI	FN	UN	TNE	NB
100% A	T1	0.0 mg/g	49.05^b^	11.49	32.77	44.26	4.79
	T2	2.0 mg/g	50.31^a^	10.56	34.69	45.25	5.06
75% A + 25% P	T3	0.0 mg/g	44.17^d^	12.78	27.93	40.71	3.46
	T4	2.0 mg/g	46.08^c^	11.69	29.76	41.45	4.63
Standard error		Interaction	0.0001	0.18	0.31	0.22	0.22
*p* value		Substituting	< 0.0001	< 0.0001	< 0.0001	< 0.0001	< 0.0001
		Spirulina	< 0.0001	< 0.0001	< 0.0001	0.0604	< 0.0001
		Interaction	< 0.0001	0.9561	0.8831	0.8799	0.1154

*A* alfalfa hay, *P Panicum maximum hay*. *T1 (Control)* 0% *Panicum maximum* (40% alfalfa hay) + no Supplementation, *T2* 0% *Panicum maximum* (40% alfalfa hay) + 2 mg/g Spirulina, *T3* 25% *Panicum maximum* (30% alfalfa hay + 10% *Panicum maximum* hay) + no Supplementation and *T4* 25% *Panicum maximum* (30% alfalfa hay + 10% *Panicum maximum* hay) + 2 mg/g Spirulina. ^a and b^ mean that different superscripts in the same row are significantly different. Nitrogen intake (TNI), Fecal nitrogen (FN), Urinary nitrogen (UN), Total nitrogen excretion (TNE), and Nitrogen balance (NB). The data in this table reflect the results from the digestion period only.

Regarding the effect of Spirulina supplementation, the findings in Table 6; Fig. 4B show that animals receiving Spirulina-supplemented diets had significantly higher (*p* < 0.0001) TNI, UN, and NB than those that received non-supplemented diets. In contrast, the animals on supplemented diets showed significantly lower (*p* = 0.9561) FN compared to those on non-supplemented diets. The interaction between forage substituting and Spirulina supplementation did not show a significant impact (*p* > 0.05) on most nitrogen utilization parameters, except for TNI, where a significant effect was observed.

### Serum blood parameters

The data presented in Table 7 indicated that animals that received diets containing 40% alfalfa hay recorded significantly higher (*p* < 0.0001) levels of TP, Alb, Gol, and urea concentrations compared to those that received diets of 10% *Panicum maximum* and 30% alfalfa hay. However, animals fed 10% *Panicum maximum* and 30% alfalfa hay recorded significantly higher (*p* < 0.0001) levels of Creat, Chol, TG, and ALT and AST enzyme activity compared to animals fed 40% alfalfa hay.

**Table 7:** Effect of substituting alfalfa hay with *Panicum maximum* hay with or without spirulina addition on blood parameters during the fattening trial.

Forage contents	TRT	Spirulina, mg/g	TP, g/dl	Alb, g/dl	Glo, g/dl	Chol, mg/dl	TG, mg/dl	Creat, mg/dl	urea, mg/dl	ALT, U/L	AST, U/L
Effect of substituting alfalfa hay with *Panicum maximum* hay											
100% A	-	-	6.44	3.55	2.89	89.80^b^	74.47	0.99	56.88	19.28	77.25
75% A + 25% P	-	-	5.97	3.27	2.70	108.96^a^	89.88	1.22	48.57	22.74	84.54
Effect of spirulina											
-	-	0.0 mg/g	6.07	3.32	2.75	104.36^a^	85.92	1.15	50.64	21.31	82.28
-	-	2.0 mg/g	6.34	3.49	2.85	94.40^b^	78.42	1.06	54.81	20.71	79.52
Effect of interaction between substituting Alfalfa hay with *Panicum maximum* hay and spirulina											
100% A	T1	0.0 mg/g	6.30	3.47	2.83	91.29^c^	77.09	1.04	53.82	19.45	78.75
	T2	2.0 mg/g	6.59	3.63	2.96	88.31^d^	71.85	0.94	59.95	19.11	75.76
75% A + 25% P	T3	0.0 mg/g	5.86	3.18	2.67	117.44^a^	94.76	1.25	47.46	23.16	85.81
	T4	2.0 mg/g	6.08	3.35	2.73	100.49^b^	85.00	1.19	49.68	22.31	83.27
Standard error			0.03	0.05	0.06	2.81	3.32	0.02	1.29	0.38	0.52
*p* value		Substituting	< 0.0001	< 0.0001	0.004	< 0.0001	0.0002	< 0.0001	< 0.0001	< 0.0001	< 0.0001
		Spirulina	< 0.0001	0.003	0.126	0.002	0.0351	0.0005	0.0041	0.1297	< 0.0001
		Interaction	0.2821	0.960	0.571	0.022	0.5037	0.3247	0.1445	0.5087	0.6649

*A* alfalfa hay, *P Panicum maximum* hay. *T1 (Control)* 0% *Panicum maximum* (40% alfalfa hay) + no Supplementation, *T2* 0% *Panicum maximum* (40% alfalfa hay) + 2 mg/g Spirulina, *T3* 25% *Panicum maximum* (30% alfalfa hay + 10% *Panicum maximum* hay) + no Supplementation and *T4* 25% *Panicum maximum* (30% alfalfa hay + 10% *Panicum maximum* hay) + 2 mg/g Spirulina. ^a and b^ mean that different superscripts in the same row are significantly different. Total protein (TP), Albumin (Alb), Globulin (Glo), Urea (urea), Creatinine (Creat), Cholesterol (Chol), Triglycerides (TG), Alanine aminotransferase (ALT), Aspartate aminotransferase (AST).

In addition, the data indicate that animals that received diets supplemented with 2 mg/g spirulina had higher TP, Alb, and urea concentrations compared to animals that did not receive the supplement, while Creat, Chol, TG, and AST activity were decreased in Spirulina-supplemented animals compared to the non-supplemented animals.

The interaction between substituting 25% of alfalfa hay with *Panicum maximum* hay and Spirulina supplementation did not have a significant effect (*p* > 0.05) on serum blood parameters, except for cholesterol concentration, where a significant interaction was observed. Cholesterol increased in T3, then decreased in T4, followed by T1, and the lowest cholesterol level was in T2.

### Growth rate and feed conversion ratio (FCR)

**Fig. 5:**
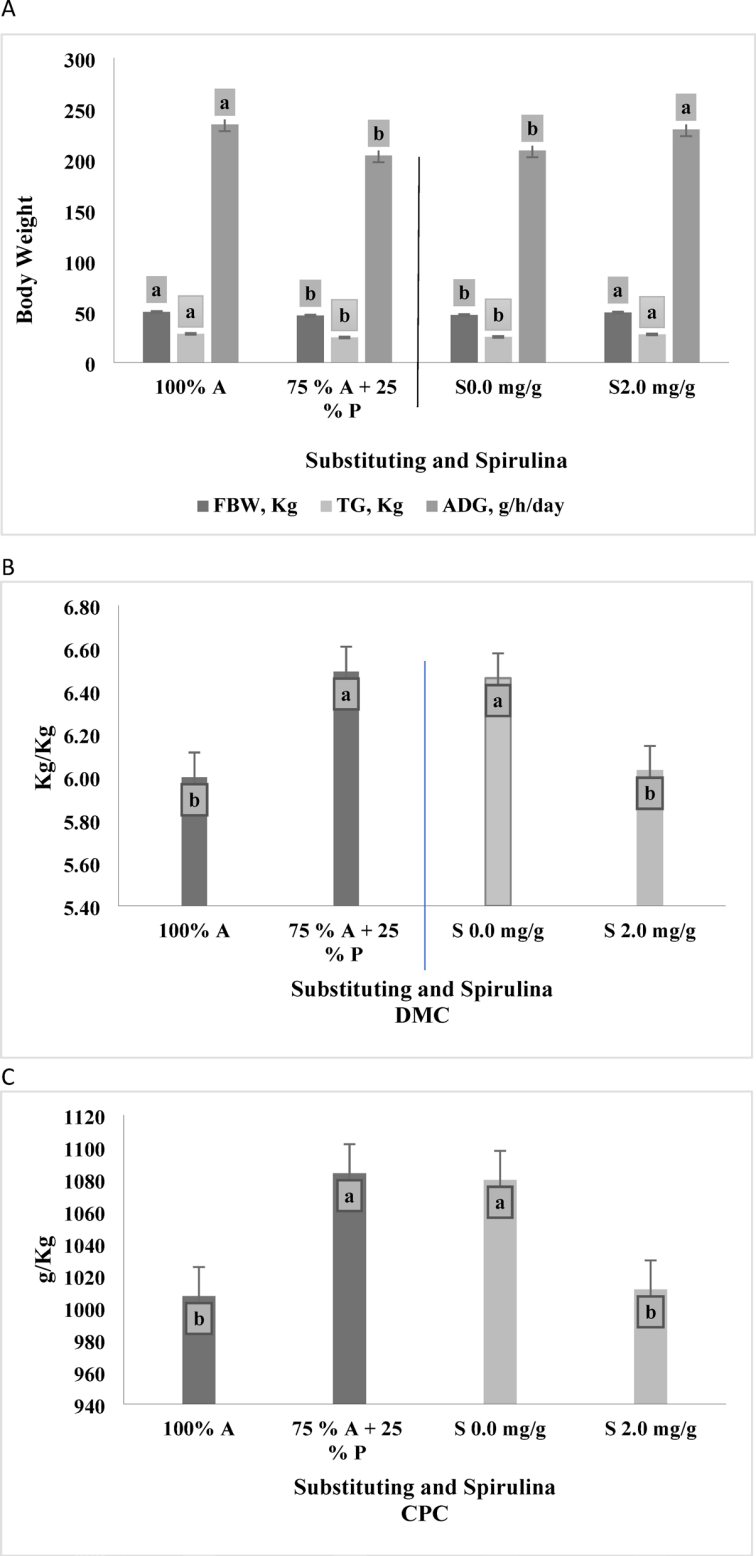
(**A**) The two main effects of substituting Alfalfa hay 100% (A), Alfalfa hay 75% (A) *Panicum maximum* hay 25% (P) and additives Spirulina 2.0 mg/g (S), 0.0 mg/g on growth rate. (**B**) The two main effects of substituting Alfalfa hay 100% (A), Alfalfa hay 75% (A) Panicum maximum hay 25% (P) and additives Spirulina 2.0 mg/g (S), 0.0 mg/g on FCR (DMC).(**C**) The two main effects of substituting Alfalfa hay 100% (A), Alfalfa hay 75% (A) Panicum maximum hay 25% (P) and additives Spirulina 2.0 mg/g (S), 0.0 mg/g on Feed Conversion Ratio (FCR) (Digestible Crude Protein Intake (DCPC)/ADG).FBW, Kg (Final body weight), TG, Kg (Total gain), ADG, g/h/day (Average daily gain). Varied letters of columns points out that there were significant differences at 0.05 level of probability. Means were distinguished employing Duncan’s multiple range test (*p* ≤ 0.05).

The data presented in Table 8; Fig. 5A indicate that animals fed diets containing 10% *Panicum maximum* and 30% alfalfa hay as forage significantly lower (*p* < 0.0001) final body weight (FBW), total gain (TG), and average daily gain (ADG) compared to those receiving diets containing 40% alfalfa hay. Additionally, the data showed that animals supplemented with 2 mg/g spirulina had significantly higher (*p* < 0.05) FBW, TG, and ADG than those that received non-supplemented diets. No significant interaction effects (*p* > 0.05) were observed between forage substituting and Spirulina supplementation for all growth performance parameters, including FBW, TG, and ADG.

**Table 8:** Effect of substituting alfalfa hay with *Panicum maximum* hay with or without spirulina addition on growth rate and feed conversion ratio (FCR) during the fattening trial.

Forage contents	TRT	Spirulina, mg/g	Growth rate				Feed conversion ratio (FCR)			
			IBW, Kg	FBW, Kg	Total gain, Kg	ADG, g/h/day	DMC, kg/kg	TDNC, kg/kg	CPC, g/kg	DCPC, g/kg
100% A	T1	0.0 mg/g	21.63	48.38	26.75	222.92	6.21	4.56	1041.60	886.35
	T2	2.0 mg/g	21.63	51.13	29.5	245.83	5.78	4.29	972.75	853.97
75% A + 25% P	T3	0.0 mg/g	21.63	44.94	23.31	194.27	6.71	4.70	1117.52	883.00
	T4	2.0 mg/g	21.63	47.2	25.58	213.12	6.28	4.54	1049.79	870.93
Standard error			1.20	1.28	0.79	6.56	0.19	0.14	32.59	26.60
*p* value		Substituting	1.000	0.0075	< 0.0001	< 0.0001	0.0174	0.1719	0.0263	0.8000
		Spirulina	1.000	0.0499	0.0036	0.0036	0.0351	0.1223	0.0453	0.4106
		Interaction	1.000	0.8501	0.7592	0.7591	0.9975	0.6852	0.9863	0.7057

*A* alfalfa hay, *P Panicum maximum* hay. *T1 (Control)*: 0% *Panicum maximum* (40% alfalfa hay) + no Supplementation, *T2* 0% *Panicum maximum* (40% alfalfa hay) + 2 mg/g Spirulina, *T3* 25% *Panicum maximum* (30% alfalfa hay + 10% *Panicum maximum* hay) + no Supplementation, and *T4* 25% *Panicum maximum* (30% alfalfa hay + 10% *Panicum maximum* hay) + 2 mg/g Spirulina. IBW, Kg (Initial body weight), FBW, Kg (Final body weight), TG, Kg (Total gain), ADG, g/h/day (Average daily gain), Feed conversion ratio: Total Dry Matter Intake (DMC)/ADG, Total Digestible Nutrients intake (TDNC)/ADG, and Digestible Crude protein intake (DCPC)/ADG.

Furthermore, the data presented in Table 8, as well as Fig. 5B,C, show that animals fed diets containing 10% *Panicum maximum* hay and 30% alfalfa hay had a significantly higher dry matter conversion (DMC) and crude protein conversion (CPC) ratios, measured as kg of intake per kg of growth, compared to those on diets with 40% alfalfa hay with *p* = 0.0174 and 0.0263, respectively. While there were no significant differences in FCR between animals fed 40% alfalfa hay and those fed 10% *Panicum maximum* hay and 30% alfalfa hay in terms of total digestible nutrients, and digestible crude protein per kilogram of growth (TDNC, and DCPC, respectively) (*p* = 0.1719 and 0.8000). Spirulina supplementation significantly improved FCR, as indicated by reductions in dry matter conversion (DMC Fig. 5B) (*p* = 0.0351) and crude protein conversion (CPC Fig. 5C) (*p* = 0.0453) compared to non-supplemented animals. However, no significant interaction effects (*p* > 0.05) were observed between forage substitution and Spirulina supplementation (0 and 2 mg/g) on FCR parameters, including DMC, TDNC, CPC, and DCPC.

## Discussion

Although the diets were formulated as isocaloric and isonitrogenous, replacing 25% of alfalfa hay with *Panicum maximum* hay significantly decreased TDNI (g/h/day and g/kgBW^0.75^) and DCPI (g/h/day and g/kgBW^0.75^). This reduction in feed intake may be attributed to the higher fiber content of *Panicum maximum*, including CF, NDF, and ADF, compared to alfalfa hay. Another reason was the low crude protein in *Panicum maximum*. The increased fiber content likely contributes to greater rumen filling, leading to earlier satiety and reduced voluntary intake. Similar findings were reported by Ajayi et al.^22^, who observed that diets high in NDF and Acid Detergent Lignin (ADL) generally lead to lower feed intake. Mupangwa et al.^23^ emphasized that the NDF content is a key indicator of rumen fill, which further explains the observed decrease in feed consumption. Similarly, Rokomatu et al.^24^ found that feeding sheep a basal diet of *Panicum maximum* significantly decreased their total dry matter intake. These findings are consistent with those reported by Ojo et al.^25^, who observed a significant increase in feed intake and growth performance among West African dwarf rams supplemented with herbaceous legume pellets (*Lablab purpureus*,* Calopogonium mucunoides*,* and Mucuna pruriens*) compared to those fed solely on *Panicum maximum*. Their results demonstrated that legume supplementation improved the nutritional quality and palatability of the diet, thereby enhancing voluntary feed consumption. Adebisi et al.^26^ reported that DMI significantly increased (*p* < 0.05) with higher inclusion levels of the legume (*Gmelina arborea leaves*) in the diet of West African Dwarf goats. The highest intake was recorded in goats fed 100% *Gmelina arborea leaves* and 0% *Panicum maximum*, while the lowest intake occurred in the 50:50 mixture group.

Conversely, Spirulina supplementation significantly increased feed intake as TDNI (g/h/day and g/kg BW^0.75^), and DCPI (g/h/day and g/kg BW^0.75^), likely due to its positive effects on rumen fermentation (NH₃ and TVFA) and nutrient digestibility. Spirulina is a rich source of crude protein (65%–70% DM), vitamins, minerals, carotenoids, γ-linolenic acid, phenolic acids, and β-carotene^27,28^. These bioactive compounds enhance microbial activity and modulate gut microbiota composition in the rumen^29,30^. Similar findings were reported by EL-Sabagh et al.^31^, who observed a significant increase in feed intake in fattening lambs supplemented with Spirulina platensis. This improvement may be attributed to the enhanced palatability of spirulina, as well as its rich nutrient profile, which could stimulate feed intake. The authors noted that even a low supplementation level (1 g/10 kg BW/day) was sufficient to elicit a measurable increase in feed consumption. Also, Peiretti & Meineri^32^ investigated the effects of increasing dietary levels of Spirulina platensis (5%, 10%, and 15%) on feed intake in growing rabbits. Although the diets were formulated as isocaloric and isonitrogenous, the rabbits receiving 10% spirulina exhibited the highest feed intake. This suggests that spirulina may enhance palatability, potentially stimulating feed intake. However, Heidarpour et al.^33^ reported that Spirulina supplementation had no significant impact on daily feed intake or feed efficiency in Holstein calves. Similarly, Yadav & Kumar^34^ observed no significant changes in DMI in Barbary goats supplemented with Spirulina at 0.25% and 0.50% of dry matter.

The differences between our results and the nonsignificant findings suggest that Spirulina supplementation can increase feed intake in certain ruminant species. However, its effects may vary based on the animal’s type, the composition of its diet, the specific species, the levels of inclusion, and the experimental conditions.

The observed increases in NH₃ concentration in the ruminal liquor of animals fed diets containing 40% alfalfa hay as forage, compared to those receiving diets with 10% *Panicum maximum* hay and 30% alfalfa hay, can be attributed to the enhanced degradability of nutrients, particularly crude protein, in the animals consuming the 40% alfalfa hay diets. Furthermore, the higher degradability of diets containing alfalfa hay, in contrast to those with varying levels of *Panicum maximum*, supports this observation^7^. These findings are consistent with the research by Xia et al.^35^, who demonstrated that increasing dietary crude protein levels in Holstein bulls led to a linear rise in ruminal NH₃ concentration (*p* < 0.01), especially in diets with higher rumen-degradable protein (RDP). The study emphasized that NH₃ production is tightly linked to both the quantity and degradability of dietary protein, as well as microbial utilization efficiency. Additionally, NH₃ production in ruminants is influenced by the concentration of rumen-degradable protein and its utilization efficiency^36^.

The observed increase in TVFA among groups consuming diets with 40% alfalfa hay (T1 and T2) compared to those consuming 10% *Panicum maximum* hay and 30% alfalfa hay may be attributed to the lower fiber content of alfalfa hay, particularly its NDF and ADF levels, in contrast to *Panicum maximum* hay. Miller et al.^37^ highlighted that the fiber content of forage affects ruminal fermentation, nutrient digestibility, and turnover due to its physical and chemical properties. Furthermore, the presence of non-fiber carbohydrates (NFC) in alfalfa hay could have contributed to the increased TVFA levels. This is supported by Wang et al.^38^, who showed that rumen bacteria ferment carbohydrates into TVFA through enzymatic processes.

The inclusion of Spirulina in the diet significantly increases the concentrations of TVFA and NH₃. This increase may result from enhanced feed intake and improved nutrient digestibility, particularly the digestibility of crude protein. Spirulina is a rich source of crude protein and contains various bioactive compounds, including polysaccharides, proteins, peptides, amino acids, lipids, polyphenols, and minerals^39^. It has been shown to stimulate the activity of rumen microflora. Panjaitan et al.^40^ Similarly reported an increase in ammonia levels was reported with Spirulina supplementation. Additionally, the high nutritional density of Spirulina may promote the secretion of fiber-digesting enzymes by rumen microbes, which enhances fiber and carbohydrate digestion and, consequently, increases TVFA concentration^41^. Costa et al.^42^; Gaafar et al.^43^ also observed a positive correlation between Spirulina supplementation and elevated levels of ruminal TVFA. Importantly, Meteab et al.^7^ demonstrated that supplementing diets with up to 2 mg/g of Spirulina significantly (*p* < 0.0001) increased both TVFA and NH₃ concentrations in vitro. Their study showed that Spirulina improved gas production and fermentation parameters, especially when combined with partial substitution of alfalfa hay by *Panicum maximum*. The optimal combination was achieved at 25% *Panicum maximum* replacement and 2 mg/g Spirulina, which maximized ruminal fermentation efficiency and nutrient degradability.

Ruminal pH was significantly influenced by sampling time and showed a normal pattern. The highest pH values were recorded at zero hours pre-feeding, followed by a decline in three hours post-feeding, and a subsequent increase at six hours post-feeding. This trend may be attributed to fluctuations in TVFA levels, which were lowest at zero hours, peaked at three hours of post-feeding, and then declined by six hours. These findings are consistent with Comtet-Marre et al.^44^, who reported that ruminal pH typically decreases within 2 to 8 h post-feeding, depending on the diet composition, before returning to baseline levels. The pH recovery is primarily due to the absorption of TVFA, salivary buffering, and the passage of digesta to the omasum^45^. Similar results were reported by El-Kholany et al.^46^, who attributed the decline in rumen pH to increased TVFA production. Additionally, Ahmed et al.^47^ found that TVFA concentrations peaked at four hours post-feeding, corresponding to a decrease in pH at that time. In the present study, the lowest pH values coincided with the highest NH₃ and TVFA concentrations at three hours post-feeding.

Rumen pH tended to be lower in animals fed 40% alfalfa hay (T1 and T2), which may be due to increased NH₃ and TVFA production. These findings agree with Osman et al.^48^ who stated that rumen pH was affected by several variables, including water intake, NH3 levels, and TVFA production. In contrast, animals receiving 10% *Panicum maximum* hay and 30% alfalfa hay without Spirulina supplementation (T3) exhibited the highest pH values, consistent with the findings of Binuomote et al.^49^, who reported that sheep fed *Panicum maximum* supplemented with *Gmelina arborea* leaves had significantly higher rumen pH compared to other diets.

Spirulina supplementation significantly lowered rumen pH compared to non-supplemented diets, likely due to the increase in NH₃ and TVFA production. These findings align with Riad et al.^50^, observed a significant pH reduction in the ruminal liquor of animals fed Spirulina-supplemented diets. Furthermore, Christodoulou et al.^51^ reported that increasing Spirulina concentration in the drinking water of cattle resulted in decreased rumen PH. However, Christodoulou et al.^52^ found no significant effect of Spirulina supplementation on rumen pH in lambs, indicating that its impact may vary based on dietary composition and animal species.

Replacing 25% of alfalfa hay with *Panicum maximum* resulted in a decline in nutrient digestibility and overall nutritive value. This reduction is likely due to the higher fiber content, including CF, NDF, ADF, and ash in *Panicum maximum*, compared to alfalfa hay, which has higher CP and OM content. This aligns with findings from^5^, who reported that *Panicum maximum cv. Mombasa* has higher fiber fractions (CF, NDF, ADF) and ash content, which negatively affect digestibility in Red Sokoto bucks. The lower CP and OM content compared to alfalfa further explains the reduced microbial activity and fermentation efficiency. As confirmed by Ali et al.^53^, who reported that dietary protein supplementation enhances CP availability for rumen microbes, thereby improving digestion. Another contributing factor to the decrease in digestibility is the impact of tannins in *Panicum maximum*. Tannins interact with proteins by forming hydrogen bonds between their phenolic groups and the carboxyl groups of proteins, altering their digestibility. These tannin-protein complexes can hinder post-ruminal digestion and protein absorption^54^. Additionally, tannins inhibit proteolytic enzymes directly and influence metabolite concentrations that regulate bacterial proteolysis^55,56^. Previous studies^57,58^ have also reported that diets rich in tannins reduce protein degradation in the rumen and lower ammonia concentrations.

On the other hand, supplementing diets with Spirulina has been shown to enhance digestibility, likely due to its rich profile of highly bioavailable nutrients^9^. Spirulina is considered an excellent feed additive for ruminants because it contains essential macro and micronutrients, including proteins, fatty acids, minerals, β-carotene, and vitamins^10^. The results of our study were aligned with those of Hassanien et al.^59^, who reported that supplementing Damascus goat diets with spirulina (0.2% spirulina (on a DM basis)) significantly improved nutrient digestibility coefficients for fiber fractions (NDF and ADF) and CP. These outcomes further support the role of Spirulina in stimulating rumen microbial activity and enhancing fermentation dynamics. Spirulina has a complete amino acid profile that significantly contributes to nutrient utilization^60,61^. Additionally, Spirulina supplementation enhances rumen microbial activity by providing essential minerals, vitamins, and fatty acids, particularly Omega-3, which improves fiber digestion and increases TDN.

Animals that received a diet containing 30% alfalfa hay and 10% *Panicum maximum* hay recorded the lowest nitrogen retention, indicated by a low NB. This reduced retention may be attributed to a significant decline in crude protein intake compared to the animals that consumed 40% alfalfa hay, which also correlated with an increase in FN. This finding aligns with the report by Mupangwa et al.^23^ who demonstrated that goats supplemented with high-protein legume hays (*Cassia rotundifolia*,* Lablab purpureus*,* and Macroptilium atropurpureum*) exhibited significantly higher nitrogen intake and retention compared to those fed a basal diet of *Chloris gayana grass* hay. Their results confirm that nitrogen retention is positively correlated with dietary protein intake and microbial activity in the rumen. Moreover, the increase in FN observed in animals fed lower CP diets may reflect inefficient nitrogen utilization, where excess dietary fiber and limited fermentable nitrogen lead to increased nitrogen losses via feces. Taken together, these observations underscore the importance of achieving a balanced supply of fermentable energy and nitrogen sources to optimize ruminal microbial efficiency and nitrogen retention. The increased urinary nitrogen and nitrogen balance observed in animals receiving diets containing 40% alfalfa hay may be due to the rapid digestion of alfalfa in the rumen, which promotes ammonia formation and thus increases urine nitrogen secretion and nitrogen balance. This is consistent with findings by Spek et al.^62^, who reported that higher crude protein intake enhances rumen fermentation, resulting in increased urinary nitrogen excretion and nitrogen balance. Conversely, the high tannin contents in the *Panicum maximum* reduced ammonia production, thereby lowering nitrogen retention. Alasa et al. Alasa et al.^63^ reported that the lowest value of nitrogen intake and nitrogen balance was observed in rams-fed *Panicum maximum* of 100%. Fecal nitrogen (FN) serves as a key indicator of diet quality and correlates with both dietary nitrogen and fiber content in herbivores^64^. Its concentration largely depends on digestibility, which can be significantly reduced by anti-nutritional factors such as tannins^55^. Accordingly, the observed improvement in nitrogen balance may be attributed to a reduction in fecal nitrogen alongside an increase in nitrogen intake.

Supplementation with spirulina has a positive effect on nitrogen balance for several reasons, including its high nutrient density and its ability to stimulate the secretion of extracellular enzymes by rumen microflora^41^. Spirulina also contains carbohydrates, which promote increased microbial protein synthesis. Previous studies have shown that both microbial protein synthesis and rumen ammonia nitrogen levels increase in a quadratic manner with higher inclusion rates of spirulina in the diet^40^.

Blood parameters are considered key indicators of an animal’s physiological and metabolic status as well as their diet quality^65^. The present results showed that animals fed a diet containing 40% alfalfa hay showed significantly higher TP, Alb, Gol, and urea concentration in their blood serum compared to those fed 10% *Panicum maximum* hay and 30% alfalfa hay. This increase may be attributed to several factors, including significant increases in CPI, DCPI, and protein digestibility for animals that received diets containing 40% alfalfa hay. Additionally, the higher microbial protein formation in the rumen, which subsequently reached the true stomach, likely contributed to increased amino acid absorption, leading to elevated total protein levels in the blood^53^. The total protein concentration in blood serum reflects the crude protein content of the diet, aligning with the findings of^6^. This increase in urea concentration may be associated with increased CP digestibility and DMI. The level of urea concentration can serve as an indicator of protein status in animals. This could assist in formulating nitrogen-balanced diets or identifying issues within a feeding program^67^. In contrast, the inclusion of *Panicum maximum* in the diet led to a decline in blood parameters (TP, Alb, Gol, and urea). This decrease may be attributed to the tannin content of *Panicum maximum*, which has been reported to lower TP concentrations when tannins are included in the diet^68^. Ojo et al.^25^ reported that West African dwarf rams fed *Panicum maximum* supplemented with herbaceous forage legume pellets (*Lablab purpureus*,* Calopogonium mucunoides*,* and Mucuna pruriens*) showed superior blood parameters (TP, Alb, Gol, and glucose) compared to rams fed only *Panicum maximum*. This may conflict with El-Kholany et al.^46^ indicated that all measured blood parameters (TP, Alb, Gol, urea, and creat) of lactating Zaraibi goats fed on different ensiled mixtures of *Panicum maximum* with berseem silage were not significantly affected by different experimental diets. The cholesterol-lowering effect of alfalfa and Spirulina is attributed to their high content of saponins, which saponins are plant compounds known to lower cholesterol levels by reducing intestinal cholesterol absorption and increasing the excretion of compounds required for cholesterol synthesis^69^.

Lambs-fed *Panicum maximum* hay showed higher levels of creat, Chol, TG, ALT, and AST, which may be attributed to the lower fiber digestibility of this forage. Experimental studies have shown that improved fiber digestibility lowers serum ALT and AST levels while increasing serum albumin and total protein concentrations^70^.

Spirulina supplementation positively influenced blood protein levels and urea concentration. The increase in TP, Alb, Gol, and urea levels observed in Spirulina-supplemented animals may be attributed to its high crude protein intake and protein digestibility. Current results were consistent with Mariey et al.^71^, who reported that increasing the TP of blood may be due to the high protein content in Spirulina. Likewise, Hafez et al.^72^ found that TP and Alb of blood were significantly higher with 0.2% Spirulina of growing lambs compared to a control group. Additionally, the results showing reduced serum cholesterol, triglycerides, creatinine, ALT, and AST levels due to dietary Spirulina were consistent with those of Khalifa et al.^73^, who reported a similar effect when adding 0.5 g per head per day for dairy goats. Moreover, EL-Sabagh et al.^31^ found that Spirulina supplementation at 1 g/10 kg/BW/day significantly increased plasma globulin while reducing the AST, ALT, Chol, and glucose concentrations. Similarly, Khalifa et al.^73^ reported that adding 500 mg/head/day of Spirulina to dairy goats significantly increased TP and glucose concentrations while reducing Chol, TG, AST, and ALT, with no significant effect on urea concentration. These findings may be attributed to the increased saponin content of Spirulina, which can be explained by Matsuura^74^, who reported that saponin and bile acids can form large mixed micelles, which increase the excretion of bile acids, thus accelerating Chol metabolism in the liver and reducing serum Chol.

The decline in average daily gain, total gain, and average daily gain associated with *Panicum maximum* hay inclusion may be attributed to reduced feed intake, altered ruminal fermentation parameters (TVFA and NH_3_), and lower nutrient digestibility, which consequently affects body weight. Furthermore, the significant increase in (FBW), total gain (TG), and (ADG) associated with Spirulina supplementation can be attributed to its positive effects on (TDNI) and (DCPI) intake, as well as its improvement of nutrient digestibility. These findings are consistent with research conducted by Bezerra et al.^75^, which showed that lambs receiving Spirulina supplementation had higher daily weight gains compared to a control group. Additionally, studies by EL-Sabagh et al.^31^ supplemented fattening lambs with Spirulina at 10% and 20% of the concentrate mixture over six weeks, resulting in significantly higher (ADG) compared to the control group. This enhancement was attributed to Spirulina’s rich content of digestible protein, essential amino acids, and bioactive compounds that collectively improve nutrient absorption and metabolic efficiency, thereby promoting superior growth. Similarly, Riad et al.^50^ reported that Friesian calves supplemented with 2 g of dried Spirulina per head per day exhibited significant increases in live body weight, body condition score, and body length, further confirming Spirulina’s growth-promoting effects across different ruminant species. These findings reinforce the current results and highlight Spirulina’s potential as a biologically active feed additive capable of enhancing growth performance through improved nutrient utilization.

Feed Conversion Ratio (FCR) of the lambs fed rations containing 30% alfalfa hay plus 10% *Panicum maximum* hay recorded the highest values as DMC and CPC compared to the lambs fed 100% of alfalfa hay, this result may be attributed to the lower feed intake, digestion, and growth rate of the groups that received *Panicum maximum* hay compared to those received alfalfa hay only. This agreed with the finding of^76^, who found that there was a relationship between growth and feed conversion of growing lambs fed varying levels of tree leaves and concentrates. The improvements in (FCR) as DMC and CPC when using Spirulina as a supplement may be due to better nutrient digestibility, increased feed intake, and enhanced average body weight gain. These results align with the research conducted by EL-Sabagh et al.^31^, which demonstrated that incorporating Spirulina into the diets of fattened lambs led to an improved average daily gain and feed conversion ratio compared to a control group. Similarly, Riad et al.^50^ reported that Friesian calves supplemented with 2 g/day of dried Spirulina exhibited improved feed efficiency, reflected in greater body weight and condition. This suggests that Spirulina enhances nutrient utilization and promotes more efficient conversion of feed into body mass, reinforcing its role as a functional additive for improving FCR in ruminants.

## Conclusion

Substituting 25% of alfalfa hay with *Panicum maximum* cv. Mombasa led to reduced feed intake, digestibility, and growth performance. However, spirulina supplementation at 2 mg/g significantly improved feed intake, rumen fermentation, nitrogen balance, and feed conversion ratios, regardless of forage type. These findings suggest that while *Panicum maximum* hay may serve as a partial forage alternative, its limitations can be mitigated through strategic supplementation, such as spirulina. This feeding strategy offers a viable option for partially replacing conventional forages, supporting sustainable livestock production under conditions of limited availability of alfalfa.

## Supplementary Information

Below is the link to the electronic supplementary material.

Supplementary Material 1

Supplementary Material 2

## Data Availability

All data and materials are owned by the authors and/or no permissions are required.

## Declarations

### Use of AI tool

We confirm that AI-assisted tools (Grammarly and Microsoft Copilot) were used solely to enhance the language and phrasing of the manuscript. These tools were not involved in generating scientific content, data analysis, or interpretation.
