# Supplementary material for: Influence of substituting 25% alfalfa hay with Panicum maximum cv. Mombasa with or without spirulina supplementation on the productive performance of fattening Barki lambs
Source: Sci Rep. 2026 Jan 10;16:1347. doi: 10.1038/s41598-025-28525-1 (PMC12796356; doi:10.1038/s41598-025-28525-1)
Supplement: Supplementary file 1 — Supplementary Material 1 [file 41598_2025_28525_MOESM1_ESM.zip › Meteab_Supplementary/Raw Data/blood fattening two ways in data.sas.pdf]

```
Data Blood fattening two;
Input P$ S$ TP   Alb   Glo   cho   TG    Creat       urea  ALT   AST ;
Cards;
P00   S00   6.25  3.45  2.80  82.09 80.11 1.02  54.95 18.44 79.74
P00   S00   6.31  3.47  2.84  79.10 74.33 1.05  56.08 20.12 79.46
P00   S00   6.37  3.40  2.97  101.49      78.89 1.09  51.40 19.18 80.21
P00   S00   6.36  3.51  2.85  102.99      76.67 1.05  54.52 19.61 79.65
P00   S00   6.30  3.44  2.86  89.55 75.89 1.02  50.42 19.75 76.09
P00   S00   6.21  3.55  2.67  92.54 76.67 1.05  55.55 19.61 77.37
P00   S20   6.68  3.59  3.09  80.60 61.11 0.90  55.07 20.21 75.07
P00   S20   6.56  3.57  2.99  83.58 58.89 0.96  55.41 19.61 76.00
P00   S20   6.56  3.61  2.96  95.52 70.00 0.85  65.65 18.08 74.54
P00   S20   6.51  3.67  2.85  92.54 66.67 0.98  66.78 18.79 76.46
P00   S20   6.54  3.68  2.86  89.55 88.89 0.96  58.36 19.07 75.75
P00   S20   6.71  3.66  3.05  88.06 85.56 0.99  58.47 18.90 76.77
P25   S00   5.74  3.33  2.42  121.94      94.44 1.23  46.65 24.56 85.23
P25   S00   5.85  3.34  2.51  114.93      92.22 1.25  48.77 23.24 84.79
P25   S00   5.85  2.90  2.95  116.42      88.89 1.21  45.38 22.07 86.93
P25   S00   5.94  2.91  3.03  114.93      95.56 1.36  47.50 24.85 87.67
P25   S00   5.83  3.27  2.56  118.96      98.89 1.25  48.17 21.56 85.79
P25   S00   5.94  3.38  2.55  117.46      98.56 1.23  48.30 22.71 84.44
P25   S20   6.09  3.47  2.61  107.46      97.78 1.15  52.70 21.66 83.51
P25   S20   6.18  3.30  2.88  108.96      95.56 1.12  52.93 23.39 84.23
P25   S20   6.00  3.34  2.66  102.99      80.00 1.20  46.51 21.20 82.58
P25   S20   6.06  3.30  2.77  100.00      82.22 1.24  47.18 22.78 82.39
P25   S20   6.15  3.37  2.78  91.04 75.56 1.15  49.21 23.07 85.12
P25   S20   6.04  3.34  2.70  92.54 78.89 1.28  49.55 21.80 81.84
;
Proc GLM;
Class P S;
Model   TP  Alb   Glo   cho   TG    Creat       urea  ALT   AST = P S
P*S ;
MEANS P S / duncan;
LSMEANS P S P*S  / STDERR;
PROC MEANS STD; VAR  TP      Alb   Glo   cho   TG    Creat       urea  ALT
     AST;
RUN;
```
