# Supplementary material for: Influence of substituting 25% alfalfa hay with Panicum maximum cv. Mombasa with or without spirulina supplementation on the productive performance of fattening Barki lambs
Source: Sci Rep. 2026 Jan 10;16:1347. doi: 10.1038/s41598-025-28525-1 (PMC12796356; doi:10.1038/s41598-025-28525-1)
Supplement: Supplementary file 1 — Supplementary Material 1 [file 41598_2025_28525_MOESM1_ESM.zip › Meteab_Supplementary/Raw Data/blood fattening two ways.pdf]

The SAS System 17:53 Friday, October 4, 2002 42

The GLM Procedure

Class Level Information

Class	Levels	Values
P	2	P00 P25
S	2	S00 S20

Number of observations 24

The SAS System 17:53 Friday, October 4, 2002 43

The GLM Procedure

Dependent Variable: TP

Source	DF	Sum of Squares	Mean Square	F Value	Pr > F
Model	3	1.76354583	0.58784861	113.32	<.0001
Error	20	0.10375000	0.00518750		
Corrected Total	23	1.86729583			

R-Square	Coeff Var	Root MSE	TP Mean
0.944438	1.159889	0.072024	6.209583

Source	DF	Type I SS	Mean Square	F Value	Pr > F
P	1	1.34900417	1.34900417	260.05	<.0001
S	1	0.40820417	0.40820417	78.69	<.0001
P*S	1	0.00633750	0.00633750	1.22	0.2821

Source	DF	Type III SS	Mean Square	F Value	Pr > F
P	1	1.34900417	1.34900417	260.05	<.0001
S	1	0.40820417	0.40820417	78.69	<.0001

P*S	1	0.00633750	0.00633750	1.22	0.2821

The GLM Procedure

Dependent Variable: Alb

Source	DF	Sum of Squares	Mean Square	F Value	Pr > F
Model	3	0.62607917	0.20869306	14.30	<.0001
Error	20	0.29181667	0.01459083		
Corrected Total	23	0.91789583			

R-Square	Coeff Var	Root MSE	Alb Mean
0.682081	3.541870	0.120793	3.410417

Source	DF	Type I SS	Mean Square	F Value	Pr > F
P	1	0.46760417	0.46760417	32.05	<.0001
S	1	0.15843750	0.15843750	10.86	0.0036
P*S	1	0.00003750	0.00003750	0.00	0.9601

Source	DF	Type III SS	Mean Square	F Value	Pr > F
P	1	0.46760417	0.46760417	32.05	<.0001
S	1	0.15843750	0.15843750	10.86	0.0036
P*S	1	0.00003750	0.00003750	0.00	0.9601

The GLM Procedure

Dependent Variable: Glo

Source	DF	Sum of Squares	Mean Square	F Value	Pr > F
Model	3	0.30074583	0.10024861	4.32	0.0168
Error	20	0.46455000	0.02322750		

Corrected Total	23	0.76529583

R-Square	Coeff Var	Root MSE	Glo Mean
0.392980	5.442251	0.152406	2.800417

Source	DF	Type I SS	Mean Square	F Value	Pr > F
P	1	0.23403750	0.23403750	10.08	0.0048
S	1	0.05900417	0.05900417	2.54	0.1267
P*S	1	0.00770417	0.00770417	0.33	0.5711

Source	DF	Type III SS	Mean Square	F Value	Pr > F
P	1	0.23403750	0.23403750	10.08	0.0048
S	1	0.05900417	0.05900417	2.54	0.1267
P*S	1	0.00770417	0.00770417	0.33	0.5711

The GLM Procedure

Dependent Variable: cho

Source	DF	Sum of Squares	Mean Square	F Value	Pr > F
Model	3	3092.340900	1030.780300	21.76	<.0001
Error	20	947.568100	47.378405		
Corrected Total	23	4039.909000			

R-Square	Coeff Var	Root MSE	cho Mean
0.765448	6.925791	6.883197	99.38500

Source	DF	Type I SS	Mean Square	F Value	Pr > F
P	1	2204.550017	2204.550017	46.53	<.0001
S	1	595.608067	595.608067	12.57	0.0020

P*S	1	292.182817	292.182817	6.17	0.0220

Source	DF	Type III SS	Mean Square	F Value	Pr > F
P	1	2204.550017	2204.550017	46.53	<.0001
S	1	595.608067	595.608067	12.57	0.0020
P*S	1	292.182817	292.182817	6.17	0.0220

The GLM Procedure

Dependent Variable: TG

Source	DF	Sum of Squares	Mean Square	F Value	Pr > F
Model	3	1792.394346	597.464782	9.05	0.0005
Error	20	1320.824350	66.041218		
Corrected Total	23	3113.218696			

R-Square	Coeff Var	Root MSE	TG Mean
0.575737	9.889101	8.126575	82.17708

Source	DF	Type I SS	Mean Square	F Value	Pr > F
P	1	1424.346338	1424.346338	21.57	0.0002
S	1	337.425004	337.425004	5.11	0.0351
P*S	1	30.623004	30.623004	0.46	0.5037

Source	DF	Type III SS	Mean Square	F Value	Pr > F
P	1	1424.346338	1424.346338	21.57	0.0002
S	1	337.425004	337.425004	5.11	0.0351
P*S	1	30.623004	30.623004	0.46	0.5037

The GLM Procedure

Dependent Variable: Creat

Source	DF	Sum of Squares	Mean Square	F Value	Pr > F
Model	3	0.36191250	0.12063750	47.23	<.0001
Error	20	0.05108333	0.00255417		
Corrected Total	23	0.41299583			

R-Square	Coeff Var	Root MSE	Creat Mean
0.876310	4.561603	0.050539	1.107917

Source	DF	Type I SS	Mean Square	F Value	Pr > F
P	1	0.31510417	0.31510417	123.37	<.0001
S	1	0.04420417	0.04420417	17.31	0.0005
P*S	1	0.00260417	0.00260417	1.02	0.3247

Source	DF	Type III SS	Mean Square	F Value	Pr > F
P	1	0.31510417	0.31510417	123.37	<.0001
S	1	0.04420417	0.04420417	17.31	0.0005
P*S	1	0.00260417	0.00260417	1.02	0.3247

The GLM Procedure

Dependent Variable: urea

Source	DF	Sum of Squares	Mean Square	F Value	Pr > F
Model	3	542.8238792	180.9412931	18.12	<.0001
Error	20	199.6964167	9.9848208		
Corrected Total	23	742.5202958			

R-Square Coeff Var Root MSE urea Mean

0.731056	5.992607	3.159877	52.72958

Source	DF	Type I SS	Mean Square	F Value	Pr > F
P	1	415.0848375	415.0848375	41.57	<.0001
S	1	104.7090375	104.7090375	10.49	0.0041
P*S	1	23.0300042	23.0300042	2.31	0.1445

Source	DF	Type III SS	Mean Square	F Value	Pr > F
P	1	415.0848375	415.0848375	41.57	<.0001
S	1	104.7090375	104.7090375	10.49	0.0041
P*S	1	23.0300042	23.0300042	2.31	0.1445

The GLM Procedure

Dependent Variable: ALT

Source	DF	Sum of Squares	Mean Square	F Value	Pr > F
Model	3	74.33881667	24.77960556	29.14	<.0001
Error	20	17.00436667	0.85021833		
Corrected Total	23	91.34318333			

R-Square	Coeff Var	Root MSE	ALT Mean
0.813841	4.388559	0.922073	21.01083

Source	DF	Type I SS	Mean Square	F Value	Pr > F
P	1	71.82960000	71.82960000	84.48	<.0001
S	1	2.12415000	2.12415000	2.50	0.1297
P*S	1	0.38506667	0.38506667	0.45	0.5087

Source	DF	Type III SS	Mean Square	F Value	Pr > F
P	1	71.82960000	71.82960000	84.48	<.0001

S	1	2.12415000	2.12415000	2.50	0.1297
P*S	1	0.38506667	0.38506667	0.45	0.5087

The GLM Procedure

Dependent Variable: AST

Source	DF	Sum of Squares	Mean Square	F Value	Pr > F
Model	3	364.3476125	121.4492042	74.50	<.0001
Error	20	32.6026500	1.6301325		
Corrected Total	23	396.9502625			

R-Square	Coeff Var	Root MSE	AST Mean
0.917867	1.578179	1.276766	80.90125

Source	DF	Type I SS	Mean Square	F Value	Pr > F
P	1	318.3545042	318.3545042	195.29	<.0001
S	1	45.6780042	45.6780042	28.02	<.0001
P*S	1	0.3151042	0.3151042	0.19	0.6649

Source	DF	Type III SS	Mean Square	F Value	Pr > F
P	1	318.3545042	318.3545042	195.29	<.0001
S	1	45.6780042	45.6780042	28.02	<.0001
P*S	1	0.3151042	0.3151042	0.19	0.6649

The GLM Procedure

Duncan's Multiple Range Test for TP

NOTE: This test controls the Type I comparisonwise error rate, not the experimentwise error
rate.

Alpha	0.05
Error Degrees of Freedom	20
Error Mean Square	0.005188

Number of Means	2
Critical Range	.06134

Means with the same letter are not significantly different.

Duncan Grouping	Mean	N	P
A	6.44[a]	12	P00
B	5.972[b]	12	P25

The GLM Procedure

Duncan's Multiple Range Test for Alb

NOTE: This test controls the Type I comparisonwise error rate, not the experimentwise error
rate.

Alpha	0.05
Error Degrees of Freedom	20
Error Mean Square	0.014591

Number of Means	2
Critical Range	.1029

Means with the same letter are not significantly different.

Duncan Grouping	Mean	N	P
A	3.55[a]	12	P00
B	3.27[b]	12	P25

The GLM Procedure

Duncan's Multiple Range Test for Glo

NOTE: This test controls the Type I comparisonwise error rate, not the experimentwise error rate.

Alpha	0.05
Error Degrees of Freedom	20
Error Mean Square	0.023228

Number of Means	2
Critical Range	.1298

Means with the same letter are not significantly different.

Duncan Grouping	Mean	N	P
A	2.89[a]	12	P00
B	2.70[b]	12	P25

The GLM Procedure

Duncan's Multiple Range Test for cho

NOTE: This test controls the Type I comparisonwise error rate, not the experimentwise error rate.

Alpha	0.05
Error Degrees of Freedom	20
Error Mean Square	47.37841

Number of Means	2
Critical Range	5.862

Means with the same letter are not significantly different.

Duncan Grouping	Mean	N	P
A	108.96[a]	12	P25
B	89.80[b]	12	P00

The GLM Procedure

Duncan's Multiple Range Test for TG

NOTE: This test controls the Type I comparisonwise error rate, not the experimentwise error rate.

Alpha	0.05
Error Degrees of Freedom	20
Error Mean Square	66.04122

Number of Means	2
Critical Range	6.921

Means with the same letter are not significantly different.

Duncan Grouping	Mean	N	P
A	89.88[a]	12	P25
B	74.47[b]	12	P00

The GLM Procedure

Duncan's Multiple Range Test for Creat

NOTE: This test controls the Type I comparisonwise error rate, not the experimentwise error rate.

Alpha	0.05
Error Degrees of Freedom	20
Error Mean Square	0.002554

Number of Means	2
Critical Range	.04304

Means with the same letter are not significantly different.

Duncan Grouping	Mean	N	P
A	1.22[a]	12	P25
B	0.99[b]	12	P00

The GLM Procedure

Duncan's Multiple Range Test for urea

NOTE: This test controls the Type I comparisonwise error rate, not the experimentwise error rate.

Alpha	0.05
Error Degrees of Freedom	20
Error Mean Square	9.984821

Number of Means	2
Critical Range	2.691

Means with the same letter are not significantly different.

Duncan Grouping	Mean	N	P
A	56.88[a]	12	P00

B	48.57[b]	12	P25

The GLM Procedure

Duncan's Multiple Range Test for ALT

NOTE: This test controls the Type I comparisonwise error rate, not the experimentwise error rate.

Alpha	0.05
Error Degrees of Freedom	20
Error Mean Square	0.850218

Number of Means	2
Critical Range	.7852

Means with the same letter are not significantly different.

Duncan Grouping	Mean	N	P
A	22.74[a]	12	P25
B	19.28[b]	12	P00

The GLM Procedure

Duncan's Multiple Range Test for AST

NOTE: This test controls the Type I comparisonwise error rate, not the experimentwise error rate.

Alpha	0.05
Error Degrees of Freedom	20
Error Mean Square	1.630132

Number of Means	2

Critical Range 1.087

Means with the same letter are not significantly different.

Duncan Grouping	Mean	N	P
A	84.54[a]	12	P25
B	77.25[b]	12	P00

The GLM Procedure

Duncan's Multiple Range Test for TP

NOTE: This test controls the Type I comparisonwise error rate, not the experimentwise error rate.

**Alpha**	**0.05**
**Error Degrees of Freedom**	**20**
**Error Mean Square**	**0.005188**

**Number of Means**	**2**
**Critical Range**	**.06134**

Means with the same letter are not significantly different.

Duncan Grouping	Mean	N	S
A	6.34[a]	12	S20
B	6.07[b]	12	S00

The GLM Procedure

Duncan's Multiple Range Test for Alb

NOTE: This test controls the Type I comparisonwise error rate, not the experimentwise error rate.

**Alpha**	**0.05**
**Error Degrees of Freedom**	**20**
**Error Mean Square**	**0.014591**

**Number of Means**	**2**
**Critical Range**	**.1029**

Means with the same letter are not significantly different.

Duncan Grouping	Mean	N	S
A	3.49[a]	12	S20
B	3.32[b]	12	S00

The GLM Procedure

Duncan's Multiple Range Test for Glo

NOTE: This test controls the Type I comparisonwise error rate, not the experimentwise error rate.

**Alpha**	**0.05**
**Error Degrees of Freedom**	**20**
**Error Mean Square**	**0.023228**

**Number of Means**	**2**
**Critical Range**	**.1298**

Means with the same letter are not significantly different.

Duncan Grouping Mean N S

Duncan Grouping	Mean	N	S
A	2.85	12	S20
A			
A	2.75	12	S00

The GLM Procedure

Duncan's Multiple Range Test for cho

NOTE: This test controls the Type I comparisonwise error rate, not the experimentwise error rate.

Alpha	0.05
Error Degrees of Freedom	20
Error Mean Square	47.37841

Number of Means	2
Critical Range	5.862

Means with the same letter are not significantly different.

Duncan Grouping	Mean	N	S
A	104.36[a]	12	S00
B	94.40[b]	12	S20

The GLM Procedure

Duncan's Multiple Range Test for TG

NOTE: This test controls the Type I comparisonwise error rate, not the experimentwise error rate.

Alpha	0.05
Error Degrees of Freedom	20
Error Mean Square	66.04122

Number of Means 2
Critical Range 6.921

Means with the same letter are not significantly different.

Duncan Grouping	Mean	N	S
A	85.92[a]	12	S00
B	78.42[b]	12	S20

The GLM Procedure

Duncan's Multiple Range Test for Creat

NOTE: This test controls the Type I comparisonwise error rate, not the experimentwise error rate.

Alpha 0.05
Error Degrees of Freedom 20
Error Mean Square 0.002554

Number of Means 2
Critical Range .04304

Means with the same letter are not significantly different.

Duncan Grouping	Mean	N	S
A	1.15[a]	12	S00
B	1.06[b]	12	S20

The GLM Procedure

Duncan's Multiple Range Test for urea

NOTE: This test controls the Type I comparisonwise error rate, not the experimentwise error rate.

**Alpha**	**0.05**
**Error Degrees of Freedom**	**20**
**Error Mean Square**	**9.984821**

**Number of Means**	**2**
**Critical Range**	**2.691**

Means with the same letter are not significantly different.

Duncan Grouping	Mean	N	S
A	54.81[a]	12	S20
B	50.64[b]	12	S00

The GLM Procedure

Duncan's Multiple Range Test for ALT

NOTE: This test controls the Type I comparisonwise error rate, not the experimentwise error rate.

**Alpha**	**0.05**
**Error Degrees of Freedom**	**20**
**Error Mean Square**	**0.850218**

**Number of Means**	**2**
**Critical Range**	**.7852**

Means with the same letter are not significantly different.

Duncan Grouping	Mean	N	S
A	21.31	12	S00
A			
A	20.71	12	S20

The GLM Procedure

Duncan's Multiple Range Test for AST

NOTE: This test controls the Type I comparisonwise error rate, not the experimentwise error rate.

Alpha	0.05
Error Degrees of Freedom	20
Error Mean Square	1.630132

Number of Means	2
Critical Range	1.087

Means with the same letter are not significantly different.

Duncan Grouping	Mean	N	S
A	82.28[a]	12	S00
B	79.52[b]	12	S20

The GLM Procedure
Least Squares Means

P	TP LSMEAN	Standard Error	Pr > |t|
P00	6.44666667	0.02079162	<.0001
P25	5.97250000	0.02079162	<.0001

P	Alb LSMEAN	Standard Error	Pr > |t|
P00	3.55000000	0.03486980	<.0001
P25	3.27083333	0.03486980	<.0001

P	Glo LSMEAN	Standard Error	Pr > |t|
P00	2.89916667	0.04399574	<.0001
P25	2.70166667	0.04399574	<.0001

P	cho LSMEAN	Standard Error	Pr > |t|
P00	89.800833	1.987008	<.0001
P25	108.969167	1.987008	<.0001

P	TG LSMEAN	Standard Error	Pr > |t|
P00	74.4733333	2.3459401	<.0001
P25	89.8808333	2.3459401	<.0001

P	Creat LSMEAN	Standard Error	Pr > |t|
P00	0.99333333	0.01458928	<.0001
P25	1.22250000	0.01458928	<.0001

P	urea LSMEAN	Standard Error	Pr > |t|
P00	56.8883333	0.9121778	<.0001
P25	48.5708333	0.9121778	<.0001

The GLM Procedure
Least Squares Means

Standard

P	ALT LSMEAN	Error	Pr > |t|
P00	19.2808333	0.2661795	<.0001
P25	22.7408333	0.2661795	<.0001

P	AST LSMEAN	Standard Error	Pr > |t|
P00	77.2591667	0.3685707	<.0001
P25	84.5433333	0.3685707	<.0001

S	TP LSMEAN	Standard Error	Pr > |t|
S00	6.07916667	0.02079162	<.0001
S20	6.34000000	0.02079162	<.0001

S	Alb LSMEAN	Standard Error	Pr > |t|
S00	3.32916667	0.03486980	<.0001
S20	3.49166667	0.03486980	<.0001

S	Glo LSMEAN	Standard Error	Pr > |t|
S00	2.75083333	0.04399574	<.0001
S20	2.85000000	0.04399574	<.0001

S	cho LSMEAN	Standard Error	Pr > |t|
S00	104.366667	1.987008	<.0001
S20	94.403333	1.987008	<.0001

S	TG LSMEAN	Standard Error	Pr > |t|
S00	85.9266667	2.3459401	<.0001
S20	78.4275000	2.3459401	<.0001

The GLM Procedure
Least Squares Means

S	Creat LSMEAN	Standard Error	Pr > |t|
S00	1.15083333	0.01458928	<.0001
S20	1.06500000	0.01458928	<.0001

S	urea LSMEAN	Standard Error	Pr > |t|
S00	50.6408333	0.9121778	<.0001
S20	54.8183333	0.9121778	<.0001

S	ALT LSMEAN	Standard Error	Pr > |t|
S00	21.3083333	0.2661795	<.0001
S20	20.7133333	0.2661795	<.0001

S	AST LSMEAN	Standard Error	Pr > |t|
S00	82.2808333	0.3685707	<.0001
S20	79.5216667	0.3685707	<.0001

P	S	TP LSMEAN	Standard Error	Pr > |t|
P00	S00	6.30000000	0.02940380	<.0001
P00	S20	6.59333333	0.02940380	<.0001
P25	S00	5.85833333	0.02940380	<.0001
P25	S20	6.08666667	0.02940380	<.0001

P	S	Alb LSMEAN	Standard Error	Pr > |t|
P00	S00	3.47000000	0.04931334	<.0001

P00	S20	3.63000000	0.04931334	<.0001
P25	S00	3.18833333	0.04931334	<.0001
P25	S20	3.35333333	0.04931334	<.0001

The GLM Procedure
Least Squares Means

P	S	Glo LSMEAN	Standard Error	Pr > |t|
P00	S00	2.83166667	0.06221937	<.0001
P00	S20	2.96666667	0.06221937	<.0001
P25	S00	2.67000000	0.06221937	<.0001
P25	S20	2.73333333	0.06221937	<.0001

P	S	cho LSMEAN	Standard Error	Pr > |t|
P00	S00	91.293333	2.810054	<.0001
P00	S20	88.308333	2.810054	<.0001
P25	S00	117.440000	2.810054	<.0001
P25	S20	100.498333	2.810054	<.0001

P	S	TG LSMEAN	Standard Error	Pr > |t|
P00	S00	77.0933333	3.3176603	<.0001
P00	S20	71.8533333	3.3176603	<.0001
P25	S00	94.7600000	3.3176603	<.0001
P25	S20	85.0016667	3.3176603	<.0001

P	S	Creat LSMEAN	Standard Error	Pr > |t|
P00	S00	1.04666667	0.02063236	<.0001
P00	S20	0.94000000	0.02063236	<.0001
P25	S00	1.25500000	0.02063236	<.0001
P25	S20	1.19000000	0.02063236	<.0001

P	S	urea LSMEAN	Standard Error	Pr > |t|

P00	S00	53.8200000	1.2900143	<.0001
P00	S20	59.9566667	1.2900143	<.0001
P25	S00	47.4616667	1.2900143	<.0001
P25	S20	49.6800000	1.2900143	<.0001

The GLM Procedure
Least Squares Means

P	S	ALT LSMEAN	Standard Error	Pr > |t|
P00	S00	19.4516667	0.3764347	<.0001
P00	S20	19.1100000	0.3764347	<.0001
P25	S00	23.1650000	0.3764347	<.0001
P25	S20	22.3166667	0.3764347	<.0001

P	S	AST LSMEAN	Standard Error	Pr > |t|
P00	S00	78.7533333	0.5212377	<.0001
P00	S20	75.7650000	0.5212377	<.0001
P25	S00	85.8083333	0.5212377	<.0001
P25	S20	83.2783333	0.5212377	<.0001

The MEANS Procedure

Variable	Std Dev
TP	0.2849329
Alb	0.1997712
Glo	0.1824109
cho	13.2532342
TG	11.6343171
Creat	0.1340013
urea	5.6818563
ALT	1.9928479
AST	4.1543600
