# Supplementary material for: Influence of substituting 25% alfalfa hay with Panicum maximum cv. Mombasa with or without spirulina supplementation on the productive performance of fattening Barki lambs
Source: Sci Rep. 2026 Jan 10;16:1347. doi: 10.1038/s41598-025-28525-1 (PMC12796356; doi:10.1038/s41598-025-28525-1)
Supplement: Supplementary file 1 — Supplementary Material 1 [file 41598_2025_28525_MOESM1_ESM.zip › Meteab_Supplementary/Raw Data/Body weight fattening two data.sas.pdf]

```
Data BW Fattening Two;
Input P$ S$ Initial    Final       BWC   ADG ;
Cards;
P00   S00   18.00 47.00 29.00 241.67
P00   S00   19.00 47.00 28.00 233.33
P00   S00   19.00 44.00 25.00 208.33
P00   S00   25.00 54.50 29.50 245.83
P00   S00   29.00 55.50 26.50 220.83
P00   S00   24.00 50.00 26.00 216.67
P00   S00   17.00 42.00 25.00 208.33
P00   S00   22.00 47.00 25.00 208.33
P00   S20   23.00 54.00 31.00 258.33
P00   S20   21.50 47.00 25.50 212.50
P00   S20   17.50 47.00 29.50 245.83
P00   S20   26.00 56.00 30.00 250.00
P00   S20   23.00 55.00 32.00 266.67
P00   S20   25.00 54.00 29.00 241.67
P00   S20   18.50 48.00 29.50 245.83
P00   S20   18.50 48.00 29.50 245.83
P25   S00   21.00 43.00 22.00 183.33
P25   S00   18.50 45.00 26.50 220.83
P25   S00   22.50 44.00 21.50 179.17
P25   S00   23.50 47.00 23.50 195.83
P25   S00   20.00 43.00 23.00 191.67
P25   S00   23.00 44.00 21.00 175.00
P25   S00   18.00 45.50 27.50 229.17
P25   S00   26.50 48.00 21.50 179.17
P25   S20   22.00 50.50 28.50 237.50
P25   S20   25.00 46.00 21.00 175.00
P25   S20   18.50 47.70 29.20 243.33
P25   S20   20.00 46.40 26.40 220.00
```

```
P25   S20   20.50 46.50 26.00 216.67
P25   S20   20.50 45.50 25.00 208.33
P25   S20   28.00 53.00 25.00 208.33
P25   S20   18.50 42.00 23.50 195.83
;
Proc GLM;
Class P S;
Model   Initial  Final      BWC   ADG = P S P*S ;
MEANS P S / duncan;
LSMEANS P S P*S  / STDERR;
PROC MEANS STD; VAR  Initial      Final      BWC   ADG;
RUN;
```
