# Supplementary material for: Influence of substituting 25% alfalfa hay with Panicum maximum cv. Mombasa with or without spirulina supplementation on the productive performance of fattening Barki lambs
Source: Sci Rep. 2026 Jan 10;16:1347. doi: 10.1038/s41598-025-28525-1 (PMC12796356; doi:10.1038/s41598-025-28525-1)
Supplement: Supplementary file 1 — Supplementary Material 1 [file 41598_2025_28525_MOESM1_ESM.zip › Meteab_Supplementary/Raw Data/BW fattening two ways.pdf]

The GLM Procedure

Class Level Information

Class	Levels	Values
P	2	P00 P25
S	2	S00 S20

Number of observations 32

The GLM Procedure

Dependent Variable: Initial

Source	DF	Sum of Squares	Mean Square	F Value	Pr > F
Model	3	0.0000000	0.0000000	0.00	1.0000
Error	28	323.5000000	11.5535714		
Corrected Total	31	323.5000000			

R-Square	Coeff Var	Root MSE	Initial Mean
0.000000	15.71817	3.399054	21.62500

Source	DF	Type I SS	Mean Square	F Value	Pr > F
P	1	0	0	0.00	1.0000
S	1	0	0	0.00	1.0000
P*S	1	0	0	0.00	1.0000

Source	DF	Type III SS	Mean Square	F Value	Pr > F
P	1	0	0	0.00	1.0000
S	1	0	0	0.00	1.0000

P*S	1	0	0	0.00	1.0000

The GLM Procedure

Dependent Variable: Final

Source	DF	Sum of Squares	Mean Square	F Value	Pr > F
Model	3	159.1384375	53.0461458	4.06	0.0163
Error	28	365.7487500	13.0624554		
Corrected Total	31	524.8871875			

R-Square	Coeff Var	Root MSE	Final Mean
0.303186	7.543830	3.614202	47.90938

Source	DF	Type I SS	Mean Square	F Value	Pr > F
P	1	108.4128125	108.4128125	8.30	0.0075
S	1	50.2503125	50.2503125	3.85	0.0599
P*S	1	0.4753125	0.4753125	0.04	0.8501

Source	DF	Type III SS	Mean Square	F Value	Pr > F
P	1	108.4128125	108.4128125	8.30	0.0075
S	1	50.2503125	50.2503125	3.85	0.0599
P*S	1	0.4753125	0.4753125	0.04	0.8501

The GLM Procedure

Dependent Variable: BWC

Source	DF	Sum of Squares	Mean Square	F Value	Pr > F
Model	3	159.1384375	53.0461458	10.69	<.0001
Error	28	138.9237500	4.9615625		

Corrected Total	31	298.0621875

R-Square	Coeff Var	Root MSE	BWC Mean
0.533910	8.474451	2.227457	26.28438

Source	DF	Type I SS	Mean Square	F Value	Pr > F
P	1	108.4128125	108.4128125	21.85	<.0001
S	1	50.2503125	50.2503125	10.13	0.0036
P*S	1	0.4753125	0.4753125	0.10	0.7592

Source	DF	Type III SS	Mean Square	F Value	Pr > F
P	1	108.4128125	108.4128125	21.85	<.0001
S	1	50.2503125	50.2503125	10.13	0.0036
P*S	1	0.4753125	0.4753125	0.10	0.7592

The GLM Procedure

Dependent Variable: ADG

Source	DF	Sum of Squares	Mean Square	F Value	Pr > F
Model	3	11050.77276	3683.59092	10.69	<.0001
Error	28	9647.57803	344.55636		
Corrected Total	31	20698.35079			

R-Square	Coeff Var	Root MSE	ADG Mean
0.533896	8.474525	18.56223	219.0356

Source	DF	Type I SS	Mean Square	F Value	Pr > F
P	1	7528.258512	7528.258512	21.85	<.0001
S	1	3489.465800	3489.465800	10.13	0.0036

P*S	1	33.048450	33.048450	0.10	0.7591

Source	DF	Type III SS	Mean Square	F Value	Pr > F
P	1	7528.258512	7528.258512	21.85	<.0001
S	1	3489.465800	3489.465800	10.13	0.0036
P*S	1	33.048450	33.048450	0.10	0.7591

The GLM Procedure

Duncan's Multiple Range Test for Initial

NOTE: This test controls the Type I comparisonwise error rate, not the experimentwise error rate.

Alpha	0.05
Error Degrees of Freedom	28
Error Mean Square	11.55357

Number of Means	2
Critical Range	2.462

Means with the same letter are not significantly different.

Duncan Grouping	Mean	N	P
A	21.62	16	P00
A			
A	21.62	16	P25

The GLM Procedure

Duncan's Multiple Range Test for Final

NOTE: This test controls the Type I comparisonwise error rate, not the experimentwise error rate.

Alpha 0.05
Error Degrees of Freedom 28
Error Mean Square 13.06246

Number of Means 2
Critical Range 2.617

Means with the same letter are not significantly different.

Duncan Grouping	Mean	N	P
A	49.75[a]	16	P00
B	46.06[b]	16	P25

The GLM Procedure

Duncan's Multiple Range Test for BWC

NOTE: This test controls the Type I comparisonwise error rate, not the experimentwise error rate.

Alpha 0.05
Error Degrees of Freedom 28
Error Mean Square 4.961562

Number of Means 2
Critical Range 1.613

Means with the same letter are not significantly different.

Duncan Grouping	Mean	N	P
A	28.12[a]	16	P00
B	24.44[b]	16	P25

The GLM Procedure

Duncan's Multiple Range Test for ADG

NOTE: This test controls the Type I comparisonwise error rate, not the experimentwise error rate.

Alpha	0.05
Error Degrees of Freedom	28
Error Mean Square	344.5564

Number of Means	2
Critical Range	13.44

Means with the same letter are not significantly different.

Duncan Grouping	Mean	N	P
A	234.37[a]	16	P00
B	203.69[b]	16	P25

The GLM Procedure

Duncan's Multiple Range Test for Initial

NOTE: This test controls the Type I comparisonwise error rate, not the experimentwise error rate.

Alpha	0.05
Error Degrees of Freedom	28
Error Mean Square	11.55357

Number of Means	2
Critical Range	2.462

Means with the same letter are not significantly different.

Duncan Grouping	Mean	N	S
A	21.62	16	S00
A			
A	21.62	16	S20

The GLM Procedure

Duncan's Multiple Range Test for Final

NOTE: This test controls the Type I comparisonwise error rate, not the experimentwise error
rate.

Alpha	0.05
Error Degrees of Freedom	28
Error Mean Square	13.06246

Number of Means	2
Critical Range	2.617

Means with the same letter are not significantly different.

Duncan Grouping	Mean	N	S
A	49.16	16	S20
A			
A	46.65	16	S00

The GLM Procedure

Duncan's Multiple Range Test for BWC

NOTE: This test controls the Type I comparisonwise error rate, not the experimentwise error

rate.

Alpha	0.05
Error Degrees of Freedom	28
Error Mean Square	4.961562

Number of Means	2
Critical Range	1.613

Means with the same letter are not significantly different.

Duncan Grouping	Mean	N	S
A	27.53[a]	16	S20
B	25.03[b]	16	S00

The GLM Procedure

Duncan's Multiple Range Test for ADG

NOTE: This test controls the Type I comparisonwise error rate, not the experimentwise error rate.

Alpha	0.05
Error Degrees of Freedom	28
Error Mean Square	344.5564

Number of Means	2
Critical Range	13.44

Means with the same letter are not significantly different.

Duncan Grouping	Mean	N	S
A	229.47[a]	16	S20

B 208.59^b 16 S00

The GLM Procedure
Least Squares Means

P	Initial LSMEAN	Standard Error	Pr > |t|
P00	21.6250000	0.8497636	<.0001
P25	21.6250000	0.8497636	<.0001

P	Final LSMEAN	Standard Error	Pr > |t|
P00	49.7500000	0.9035505	<.0001
P25	46.0687500	0.9035505	<.0001

P	BWC LSMEAN	Standard Error	Pr > |t|
P00	28.1250000	0.5568641	<.0001
P25	24.4437500	0.5568641	<.0001

P	ADG LSMEAN	Standard Error	Pr > |t|
P00	234.373750	4.640557	<.0001
P25	203.697500	4.640557	<.0001

S	Initial LSMEAN	Standard Error	Pr > |t|
S00	21.6250000	0.8497636	<.0001
S20	21.6250000	0.8497636	<.0001

S	Final LSMEAN	Standard Error	Pr > |t|
S00	46.6562500	0.9035505	<.0001

S20	49.1625000	0.9035505	<.0001

S	BWC LSMEAN	Standard Error	Pr > |t|
S00	25.0312500	0.5568641	<.0001
S20	27.5375000	0.5568641	<.0001

The GLM Procedure
Least Squares Means

S	ADG LSMEAN	Standard Error	Pr > |t|
S00	208.593125	4.640557	<.0001
S20	229.478125	4.640557	<.0001

P	S	Initial LSMEAN	Standard Error	Pr > |t|
P00	S00	21.6250000	1.2017472	<.0001
P00	S20	21.6250000	1.2017472	<.0001
P25	S00	21.6250000	1.2017472	<.0001
P25	S20	21.6250000	1.2017472	<.0001

P	S	Final LSMEAN	Standard Error	Pr > |t|
P00	S00	48.3750000	1.2778133	<.0001
P00	S20	51.1250000	1.2778133	<.0001
P25	S00	44.9375000	1.2778133	<.0001
P25	S20	47.2000000	1.2778133	<.0001

P	S	BWC LSMEAN	Standard Error	Pr > |t|
P00	S00	26.7500000	0.7875248	<.0001
P00	S20	29.5000000	0.7875248	<.0001
P25	S00	23.3125000	0.7875248	<.0001
P25	S20	25.5750000	0.7875248	<.0001

P	S	ADG LSMEAN	Standard Error	Pr > |t|
P00	S00	222.915000	6.562739	<.0001
P00	S20	245.832500	6.562739	<.0001
P25	S00	194.271250	6.562739	<.0001
P25	S20	213.123750	6.562739	<.0001

The MEANS Procedure

ƒƒƒƒƒƒƒƒƒƒƒƒƒƒƒƒƒƒƒƒƒƒƒƒƒ

Variable	Std Dev
Initial	3.2304000
Final	4.1148323
BWC	3.1007917
ADG	25.8396737

ƒƒƒƒƒƒƒƒƒƒƒƒƒƒƒƒƒƒƒƒƒƒƒƒƒ
