# Supplementary material for: Influence of substituting 25% alfalfa hay with Panicum maximum cv. Mombasa with or without spirulina supplementation on the productive performance of fattening Barki lambs
Source: Sci Rep. 2026 Jan 10;16:1347. doi: 10.1038/s41598-025-28525-1 (PMC12796356; doi:10.1038/s41598-025-28525-1)
Supplement: Supplementary file 1 — Supplementary Material 1 [file 41598_2025_28525_MOESM1_ESM.zip › Meteab_Supplementary/Raw Data/diegstibility data 11.sas.pdf]

```
Data Didestibility fattening two;
Input P$ S$ DM    OM    CP    EE    CF    NDF   ADF   NFE
      NFC   DCP   TDN ;
Cards;
P00   S00   797.58      816.71      774.18      798.17
      641.71      689.63      621.76      852.17      909.14
      144.16      748.28
P00   S00   777.38      796.94      767.63      756.42
      599.92      653.04      585.08      835.71      900.02
      142.94      730.18
P00   S00   781.66      801.03      763.21      769.44
      609.60      658.73      587.19      840.52      906.02
      142.12      733.92
P00   S00   772.76      793.05      756.89      807.44
      599.76      644.33      559.14      830.65      900.08
      140.94      726.61
P00   S00   784.53      804.51      767.25      809.33
      610.30      666.16      601.04      842.77      903.17
      142.87      737.10
P00   S00   772.62      793.09      765.95      796.97
      598.21      646.72      572.60      828.65      894.45
      142.63      726.65
P00   S20   775.41      791.40      782.87      826.29
      606.08      664.30      587.56      816.77      865.22
      146.31      725.02
P00   S20   781.73      796.56      787.02      821.91
      613.23      676.70      596.46      822.01      864.99
      147.08      729.75
P00   S20   779.44      796.09      783.35      804.65
      620.47      670.70      590.18      821.28      872.02
      146.40      729.32
```

P00 S20 809.77 825.67 795.28 857.33
663.66 713.51 653.12 850.93 895.64
148.63 756.41
P00 S20 798.53 814.99 791.04 853.30
651.01 690.49 630.95 838.73 892.64
147.83 746.64
P00 S20 819.81 834.69 801.65 867.38
680.80 728.87 672.96 858.29 899.95
149.82 764.68
P25 S00 742.31 768.28 711.84 737.92
521.97 581.07 483.62 831.59 926.71
131.94 702.19
P25 S00 737.80 764.83 710.63 740.02
504.28 581.63 477.49 831.20 918.38
131.72 699.03
P25 S00 739.81 765.09 709.68 738.37
505.15 577.13 489.89 831.78 923.58
131.54 699.27
P25 S00 744.80 771.07 710.07 738.75
524.96 588.64 482.04 835.83 927.48
131.61 704.74
P25 S00 738.25 765.26 713.87 728.57
528.98 577.74 479.13 824.52 922.11
132.32 699.42
P25 S00 736.17 764.24 707.57 746.43
504.13 577.03 458.57 830.98 922.07
131.15 698.49
P25 S20 769.51 788.84 745.72 778.60
598.20 635.17 513.14 830.36 908.84
138.73 720.81

```
P25   S20   764.09      783.52      740.71      776.78
      568.44      626.49      502.14      831.91      906.34
      137.80      716.04
P25   S20   788.58      806.55      755.85      789.16
      598.51      657.50      532.22      855.36      925.79
      140.61      737.09
P25   S20   746.39      768.50      728.19      754.44
      527.55      599.02      472.91      824.11      902.44
      135.47      702.31
P25   S20   786.17      804.04      755.80      795.21
      623.17      664.61      567.74      844.14      913.02
      140.60      734.80
P25   S20   776.92      795.53      751.99      779.41
      613.93      649.00      531.57      834.92      909.49
      139.89      727.02
;
Proc GLM;
Class P S;
Model    DM  OM    CP    EE    CF    NDF   ADF   NFE   NFC
      DCP   TDN = P S P*S /SS3;
MEANS P S / duncan;
LSMEANS P S P*S  / STDERR;
PROC MEANS STD; VAR  DM      OM    CP    EE    CF    NDF
      ADF   NFE   NFC   DCP   TDN;
RUN;
```
