# Supplementary material for: Influence of substituting 25% alfalfa hay with Panicum maximum cv. Mombasa with or without spirulina supplementation on the productive performance of fattening Barki lambs
Source: Sci Rep. 2026 Jan 10;16:1347. doi: 10.1038/s41598-025-28525-1 (PMC12796356; doi:10.1038/s41598-025-28525-1)
Supplement: Supplementary file 1 — Supplementary Material 1 [file 41598_2025_28525_MOESM1_ESM.zip › Meteab_Supplementary/Raw Data/diegstive result two 11.lst.pdf]

The GLM Procedure

Class Level Information

Class	Levels	Values
P	2	P00 P25
S	2	S00 S20

Number of observations 24

The GLM Procedure

Dependent Variable: DM

Source	DF	Sum of Squares	Mean Square	F Value	Pr > F
Model	3	9627.71868	3209.23956	19.12	<.0001
Error	20	3357.77070	167.88853		
Corrected Total	23	12985.48938			

R-Square	Coeff Var	Root MSE	DM Mean
0.741421	1.678933	12.95718	771.7508

Source	DF	Type III SS	Mean Square	F Value	Pr > F
P	1	6029.974017	6029.974017	35.92	<.0001
S	1	3052.819267	3052.819267	18.18	0.0004
P*S	1	544.925400	544.925400	3.25	0.0867

The GLM Procedure

Dependent Variable: OM

Source	DF	Sum of Squares	Mean Square	F Value	Pr > F
Model	3	6313.657433	2104.552478	13.86	<.0001
Error	20	3035.908900	151.795445		
Corrected Total	23	9349.566333			

R-Square	Coeff Var	Root MSE	OM Mean
0.675289	1.555419	12.32053	792.1033

Source	DF	Type III SS	Mean Square	F Value	Pr > F
P	1	4239.510017	4239.510017	27.93	<.0001
S	1	1704.883267	1704.883267	11.23	0.0032
P*S	1	369.264150	369.264150	2.43	0.1345

The GLM Procedure

Dependent Variable: CP

Source	DF	Sum of Squares	Mean Square	F Value	Pr > F
Model	3	20337.83750	6779.27917	132.14	<.0001
Error	20	1026.04570	51.30229		
Corrected Total	23	21363.88320			

R-Square	Coeff Var	Root MSE	CP Mean
0.951973	0.950875	7.162561	753.2600

Source	DF	Type III SS	Mean Square	F Value	Pr > F
P	1	14721.30667	14721.30667	286.95	<.0001
S	1	5421.02042	5421.02042	105.67	<.0001
P*S	1	195.51042	195.51042	3.81	0.0651

The GLM Procedure

Dependent Variable: EE

Source	DF	Sum of Squares	Mean Square	F Value	Pr > F
Model	3	30525.50251	10175.16750	31.51	<.0001
Error	20	6458.91008	322.94550		
Corrected Total	23	36984.41260			

R-Square	Coeff Var	Root MSE	EE Mean
0.825361	2.285342	17.97068	786.3454

Source	DF	Type III SS	Mean Square	F Value	Pr > F
P	1	18424.37920	18424.37920	57.05	<.0001
S	1	11998.82320	11998.82320	37.15	<.0001
P*S	1	102.30010	102.30010	0.32	0.5798

The GLM Procedure

Dependent Variable: CF

Source	DF	Sum of Squares	Mean Square	F Value	Pr > F
Model	3	50667.59888	16889.19963	26.44	<.0001
Error	20	12775.03990	638.75200		
Corrected Total	23	63442.63878			

R-Square	Coeff Var	Root MSE	CF Mean
0.798636	4.297607	25.27354	588.0842

Source	DF	Type III SS	Mean Square	F Value	Pr > F
P	1	31936.05127	31936.05127	50.00	<.0001
S	1	15814.77360	15814.77360	24.76	<.0001
P*S	1	2916.77402	2916.77402	4.57	0.0451

The GLM Procedure

Dependent Variable: NDF

Source	DF	Sum of Squares	Mean Square	F Value	Pr > F
Model	3	38888.20295	12962.73432	34.01	<.0001
Error	20	7623.49325	381.17466		
Corrected Total	23	46511.69620			

R-Square	Coeff Var	Root MSE	NDF Mean
0.836095	3.039060	19.52369	642.4254

Source	DF	Type III SS	Mean Square	F Value	Pr > F
P	1	25882.51760	25882.51760	67.90	<.0001
S	1	11904.20584	11904.20584	31.23	<.0001
P*S	1	1101.47950	1101.47950	2.89	0.1046

The GLM Procedure

Dependent Variable: ADF

Source	DF	Sum of Squares	Mean Square	F Value	Pr > F
Model	3	75596.50875	25198.83625	34.52	<.0001

Source	DF	Sum of Squares	Mean Square	F Value	Pr > F
Error	20	14597.96643	729.89832		
Corrected Total	23	90194.47518			

R-Square	Coeff Var	Root MSE	ADF Mean
0.838150	4.894132	27.01663	552.0208

Source	DF	Type III SS	Mean Square	F Value	Pr > F
P	1	66948.29402	66948.29402	91.72	<.0001
S	1	8565.48167	8565.48167	11.74	0.0027
P*S	1	82.73307	82.73307	0.11	0.7399

The GLM Procedure

Dependent Variable: NFE

Source	DF	Sum of Squares	Mean Square	F Value	Pr > F
Model	3	185.620483	61.873494	0.48	0.6987
Error	20	2568.937700	128.446885		
Corrected Total	23	2754.558183			

R-Square	Coeff Var	Root MSE	NFE Mean
0.067387	1.356948	11.33344	835.2158

Source	DF	Type III SS	Mean Square	F Value	Pr > F
P	1	42.0820167	42.0820167	0.33	0.5734
S	1	6.4480667	6.4480667	0.05	0.8250
P*S	1	137.0904000	137.0904000	1.07	0.3139

The GLM Procedure

Dependent Variable: NFC

Source	DF	Sum of Squares	Mean Square	F Value	Pr > F
Model	3	5533.394879	1844.464960	20.43	<.0001
Error	20	1805.590283	90.279514		
Corrected Total	23	7338.985163			

R-Square Coeff Var Root MSE NFC Mean

0.753973	1.050399	9.501553	904.5663

Source	DF	Type III SS	Mean Square	F Value	Pr > F
P	1	3823.102838	3823.102838	42.35	<.0001
S	1	1614.252038	1614.252038	17.88	0.0004
P*S	1	96.040004	96.040004	1.06	0.3147

The GLM Procedure

Dependent Variable: DCP

Source	DF	Sum of Squares	Mean Square	F Value	Pr > F
Model	3	813.4734792	271.1578264	152.56	<.0001
Error	20	35.5488167	1.7774408		
Corrected Total	23	849.0222958			

R-Square	Coeff Var	Root MSE	DCP Mean
0.958130	0.950845	1.333207	140.2129

Source	DF	Type III SS	Mean Square	F Value	Pr > F
P	1	583.6134375	583.6134375	328.34	<.0001
S	1	223.4430375	223.4430375	125.71	<.0001
P*S	1	6.4170042	6.4170042	3.61	0.0719

The GLM Procedure

Dependent Variable: TDN

Source	DF	Sum of Squares	Mean Square	F Value	Pr > F
Model	3	5809.088546	1936.362849	15.22	<.0001
Error	20	2544.169417	127.208471		
Corrected Total	23	8353.257962			

R-Square	Coeff Var	Root MSE	TDN Mean
0.695428	1.556057	11.27867	724.8238

Source	DF	Type III SS	Mean Square	F Value	Pr > F
P	1	4091.175938	4091.175938	32.16	<.0001
S	1	1410.820004	1410.820004	11.09	0.0033
P*S	1	307.092604	307.092604	2.41	0.1359

The GLM Procedure

Duncan's Multiple Range Test for DM

NOTE: This test controls the Type I comparisonwise error rate, not the experimentwise error rate.

Alpha	0.05
Error Degrees of Freedom	20
Error Mean Square	167.8885

Number of Means	2
Critical Range	11.03

Means with the same letter are not significantly different.

Duncan Grouping	Mean	N	P
A	787.602	12	P00
B	755.900	12	P25

The GLM Procedure

Duncan's Multiple Range Test for OM

NOTE: This test controls the Type I comparisonwise error rate, not the experimentwise error rate.

Alpha	0.05
Error Degrees of Freedom	20
Error Mean Square	151.7954

Number of Means	2
Critical Range	10.49

Means with the same letter are not significantly different.

Duncan Grouping	Mean	N	P
A	805.394	12	P00
B	778.813	12	P25

The GLM Procedure

Duncan's Multiple Range Test for CP

NOTE: This test controls the Type I comparisonwise error rate, not the experimentwise error rate.

Alpha	0.05
Error Degrees of Freedom	20
Error Mean Square	51.30229

Number of Means	2
Critical Range	6.100

Means with the same letter are not significantly different.

Duncan Grouping	Mean	N	P
A	778.027	12	P00
B	728.493	12	P25

The GLM Procedure

Duncan's Multiple Range Test for EE

NOTE: This test controls the Type I comparisonwise error rate, not the experimentwise error rate.

Alpha	0.05
Error Degrees of Freedom	20
Error Mean Square	322.9455

Number of Means	2
Critical Range	15.30

Means with the same letter are not significantly different.

Duncan Grouping	Mean	N	P
A	814.052	12	P00
B	758.638	12	P25

The GLM Procedure

Duncan's Multiple Range Test for CF

NOTE: This test controls the Type I comparisonwise error rate, not the experimentwise error rate.

Alpha	0.05
Error Degrees of Freedom	20

Error Mean Square	638.752

Number of Means	2
Critical Range	21.52

Means with the same letter are not significantly different.

Duncan Grouping	Mean	N	P
A	624.56	12	P00
B	551.61	12	P25

The GLM Procedure

Duncan's Multiple Range Test for NDF

NOTE: This test controls the Type I comparisonwise error rate, not the experimentwise error rate.

Alpha	0.05
Error Degrees of Freedom	20
Error Mean Square	381.1747

Number of Means	2
Critical Range	16.63

Means with the same letter are not significantly different.

Duncan Grouping	Mean	N	P
A	675.265	12	P00
B	609.586	12	P25

The GLM Procedure

Duncan's Multiple Range Test for ADF

NOTE: This test controls the Type I comparisonwise error rate, not the experimentwise error rate.

Alpha	0.05
Error Degrees of Freedom	20
Error Mean Square	729.8983

Number of Means	2
Critical Range	23.01

Means with the same letter are not significantly different.

Duncan Grouping	Mean	N	P
A	604.84	12	P00
B	499.21	12	P25

The GLM Procedure

Duncan's Multiple Range Test for NFE

NOTE: This test controls the Type I comparisonwise error rate, not the experimentwise error rate.

Alpha	0.05
Error Degrees of Freedom	20
Error Mean Square	128.4469

Number of Means	2
Critical Range	9.651

Means with the same letter are not significantly different.

Duncan Grouping	Mean	N	P
A	836.540	12	P00
A			
A	833.892	12	P25

The GLM Procedure

Duncan's Multiple Range Test for NFC

NOTE: This test controls the Type I comparisonwise error rate, not the experimentwise error rate.

Alpha	0.05
Error Degrees of Freedom	20
Error Mean Square	90.27951

Number of Means	2
Critical Range	8.091

Means with the same letter are not significantly different.

Duncan Grouping	Mean	N	P

A	917.188	12	P25
B	891.945	12	P00

The GLM Procedure

Duncan's Multiple Range Test for DCP

NOTE: This test controls the Type I comparisonwise error rate, not the experimentwise error rate.

Alpha	0.05
Error Degrees of Freedom	20
Error Mean Square	1.777441

Number of Means	2
Critical Range	1.135

Means with the same letter are not significantly different.

Duncan Grouping	Mean	N	P
A	145.1442	12	P00
B	135.2817	12	P25

The GLM Procedure

Duncan's Multiple Range Test for TDN

NOTE: This test controls the Type I comparisonwise error rate, not the experimentwise error rate.

Alpha	0.05
Error Degrees of Freedom	20
Error Mean Square	127.2085

Number of Means	2
Critical Range	9.605

Means with the same letter are not significantly different.

Duncan Grouping	Mean	N	P
A	737.880	12	P00
B	711.768	12	P25

The GLM Procedure

Duncan's Multiple Range Test for DM

NOTE: This test controls the Type I comparisonwise error rate, not the experimentwise error rate.

Alpha	0.05
Error Degrees of Freedom	20
Error Mean Square	167.8885

Number of Means	2
Critical Range	11.03

Means with the same letter are not significantly different.

Duncan Grouping	Mean	N	S
A	783.029	12	S20
B	760.473	12	S00

Duncan's Multiple Range Test for OM

NOTE: This test controls the Type I comparisonwise error rate, not the experimentwise error rate.

Alpha	0.05
Error Degrees of Freedom	20
Error Mean Square	151.7954

Number of Means	2
Critical Range	10.49

Means with the same letter are not significantly different.

Duncan Grouping	Mean	N	S
A	800.532	12	S20
B	783.675	12	S00

Duncan's Multiple Range Test for CP

NOTE: This test controls the Type I comparisonwise error rate, not the experimentwise error rate.

Alpha	0.05
Error Degrees of Freedom	20
Error Mean Square	51.30229

Number of Means	2
Critical Range	6.100

Means with the same letter are not significantly different.

Duncan Grouping	Mean	N	S
A	768.289	12	S20
B	738.231	12	S00

The GLM Procedure

Duncan's Multiple Range Test for EE

NOTE: This test controls the Type I comparisonwise error rate, not the experimentwise error rate.

Alpha	0.05
Error Degrees of Freedom	20
Error Mean Square	322.9455

Number of Means	2
Critical Range	15.30

Means with the same letter are not significantly different.

Duncan Grouping	Mean	N	S
A	808.705	12	S20
B	763.986	12	S00

The GLM Procedure

Duncan's Multiple Range Test for CF

NOTE: This test controls the Type I comparisonwise error rate, not the experimentwise error rate.

Alpha	0.05
Error Degrees of Freedom	20
Error Mean Square	638.752

Number of Means	2
Critical Range	21.52

Means with the same letter are not significantly different.

Duncan Grouping	Mean	N	S
A	613.75	12	S20
B	562.41	12	S00

The GLM Procedure

Duncan's Multiple Range Test for NDF

NOTE: This test controls the Type I comparisonwise error rate, not the experimentwise error rate.

Alpha	0.05
Error Degrees of Freedom	20
Error Mean Square	381.1747

Number of Means	2
Critical Range	16.63

Means with the same letter are not significantly different.

Duncan Grouping	Mean	N	S
A	664.697	12	S20
B	620.154	12	S00

The GLM Procedure

Duncan's Multiple Range Test for ADF

NOTE: This test controls the Type I comparisonwise error rate, not the experimentwise error rate.

Alpha	0.05
Error Degrees of Freedom	20
Error Mean Square	729.8983

Number of Means	2
Critical Range	23.01

Means with the same letter are not significantly different.

Duncan Grouping	Mean	N	S
A	570.91	12	S20
B	533.13	12	S00

The GLM Procedure

Duncan's Multiple Range Test for NFE

NOTE: This test controls the Type I comparisonwise error rate, not the experimentwise error rate.

Alpha	0.05
Error Degrees of Freedom	20
Error Mean Square	128.4469

Number of Means	2
Critical Range	9.651

Means with the same letter are not significantly different.

Duncan Grouping		Mean	N	S
A		835.734	12	S20
	A			
A		834.698	12	S00

The GLM Procedure

Duncan's Multiple Range Test for NFC

NOTE: This test controls the Type I comparisonwise error rate, not the experimentwise error rate.

Alpha	0.05
Error Degrees of Freedom	20
Error Mean Square	90.27951

Number of Means	2
Critical Range	8.091

Means with the same letter are not significantly different.

Duncan Grouping	Mean	N	S
A	912.768	12	S00
B	896.365	12	S20

The GLM Procedure

Duncan's Multiple Range Test for DCP

NOTE: This test controls the Type I comparisonwise error rate, not the experimentwise error rate.

Alpha	0.05
Error Degrees of Freedom	20
Error Mean Square	1.777441

Number of Means	2
Critical Range	1.135

Means with the same letter are not significantly different.

Duncan Grouping	Mean	N	S
A	143.2642	12	S20
B	137.1617	12	S00

The GLM Procedure

Duncan's Multiple Range Test for TDN

NOTE: This test controls the Type I comparisonwise error rate, not the experimentwise error rate.

Alpha	0.05
Error Degrees of Freedom	20
Error Mean Square	127.2085

Number of Means	2
Critical Range	9.605

Means with the same letter are not significantly different.

Duncan Grouping	Mean	N	S
A	732.491	12	S20
B	717.157	12	S00

The GLM Procedure
Least Squares Means

Standard

P	DM LSMEAN	Standard Error	Pr > |t|
P00	787.601667	3.740416	<.0001
P25	755.900000	3.740416	<.0001

P	OM LSMEAN	Standard Error	Pr > |t|
P00	805.394167	3.556630	<.0001
P25	778.812500	3.556630	<.0001

P	CP LSMEAN	Standard Error	Pr > |t|
P00	778.026667	2.067653	<.0001
P25	728.493333	2.067653	<.0001

P	EE LSMEAN	Standard Error	Pr > |t|
P00	814.052500	5.187690	<.0001
P25	758.638333	5.187690	<.0001

P	CF LSMEAN	Standard Error	Pr > |t|
P00	624.562500	7.295844	<.0001
P25	551.605833	7.295844	<.0001

P	NDF LSMEAN	Standard Error	Pr > |t|
P00	675.265000	5.636005	<.0001
P25	609.585833	5.636005	<.0001

P	ADF LSMEAN	Standard Error	Pr > |t|
P00	604.836667	7.799029	<.0001
P25	499.205000	7.799029	<.0001

The GLM Procedure
Least Squares Means

P	NFE LSMEAN	Standard Error	Pr > |t|
P00	836.540000	3.271683	<.0001
P25	833.891667	3.271683	<.0001

P	NFC LSMEAN	Standard Error	Pr > |t|

P00	891.945000	2.742862	<.0001
P25	917.187500	2.742862	<.0001

P	DCP LSMEAN	Standard Error	Pr > |t|
P00	145.144167	0.384864	<.0001
P25	135.281667	0.384864	<.0001

P	TDN LSMEAN	Standard Error	Pr > |t|
P00	737.880000	3.255873	<.0001
P25	711.767500	3.255873	<.0001

S	DM LSMEAN	Standard Error	Pr > |t|
S00	760.472500	3.740416	<.0001
S20	783.029167	3.740416	<.0001

S	OM LSMEAN	Standard Error	Pr > |t|
S00	783.675000	3.556630	<.0001
S20	800.531667	3.556630	<.0001

S	CP LSMEAN	Standard Error	Pr > |t|
S00	738.230833	2.067653	<.0001
S20	768.289167	2.067653	<.0001

The GLM Procedure
Least Squares Means

S	EE LSMEAN	Standard Error	Pr > |t|
S00	763.985833	5.187690	<.0001
S20	808.705000	5.187690	<.0001

S	CF LSMEAN	Standard Error	Pr > |t|
S00	562.414167	7.295844	<.0001
S20	613.754167	7.295844	<.0001

S	NDF LSMEAN	Standard Error	Pr > |t|

S00	620.154167	5.636005	<.0001
S20	664.696667	5.636005	<.0001

S	ADF LSMEAN	Standard Error	Pr > |t|
S00	533.129167	7.799029	<.0001
S20	570.912500	7.799029	<.0001

S	NFE LSMEAN	Standard Error	Pr > |t|
S00	834.697500	3.271683	<.0001
S20	835.734167	3.271683	<.0001

S	NFC LSMEAN	Standard Error	Pr > |t|
S00	912.767500	2.742862	<.0001
S20	896.365000	2.742862	<.0001

S	DCP LSMEAN	Standard Error	Pr > |t|
S00	137.161667	0.384864	<.0001
S20	143.264167	0.384864	<.0001

The GLM Procedure
Least Squares Means

S	TDN LSMEAN	Standard Error	Pr > |t|
S00	717.156667	3.255873	<.0001
S20	732.490833	3.255873	<.0001

P	S	DM LSMEAN	Standard Error	Pr > |t|
P00	S00	781.088333	5.289747	<.0001
P00	S20	794.115000	5.289747	<.0001
P25	S00	739.856667	5.289747	<.0001
P25	S20	771.943333	5.289747	<.0001

P	S	OM LSMEAN	Standard Error	Pr > |t|
P00	S00	800.888333	5.029835	<.0001
P00	S20	809.900000	5.029835	<.0001
P25	S00	766.461667	5.029835	<.0001
P25	S20	791.163333	5.029835	<.0001

P	S	CP LSMEAN	Standard Error	Pr > |t|
P00	S00	765.851667	2.924103	<.0001
P00	S20	790.201667	2.924103	<.0001
P25	S00	710.610000	2.924103	<.0001
P25	S20	746.376667	2.924103	<.0001

P	S	EE LSMEAN	Standard Error	Pr > |t|
P00	S00	789.628333	7.336501	<.0001
P00	S20	838.476667	7.336501	<.0001
P25	S00	738.343333	7.336501	<.0001
P25	S20	778.933333	7.336501	<.0001

P	S	CF LSMEAN	Standard Error	Pr > |t|
P00	S00	609.916667	10.317881	<.0001
P00	S20	639.208333	10.317881	<.0001
P25	S00	514.911667	10.317881	<.0001
P25	S20	588.300000	10.317881	<.0001

The GLM Procedure
Least Squares Means

P	S	NDF LSMEAN	Standard Error	Pr > |t|
P00	S00	659.768333	7.970515	<.0001
P00	S20	690.761667	7.970515	<.0001
P25	S00	580.540000	7.970515	<.0001
P25	S20	638.631667	7.970515	<.0001

P	S	ADF LSMEAN	Standard Error	Pr > |t|
P00	S00	587.801667	11.029493	<.0001
P00	S20	621.871667	11.029493	<.0001
P25	S00	478.456667	11.029493	<.0001
P25	S20	519.953333	11.029493	<.0001

P	S	NFE LSMEAN	Standard Error	Pr > |t|
P00	S00	838.411667	4.626858	<.0001
P00	S20	834.668333	4.626858	<.0001
P25	S00	830.983333	4.626858	<.0001
P25	S20	836.800000	4.626858	<.0001

P	S	NFC LSMEAN	Standard Error	Pr > |t|

P00	S00	902.146667	3.878993	<.0001
P00	S20	881.743333	3.878993	<.0001
P25	S00	923.388333	3.878993	<.0001
P25	S20	910.986667	3.878993	<.0001

P	S	DCP LSMEAN	Standard Error	Pr > |t|
P00	S00	142.610000	0.544279	<.0001
P00	S20	147.678333	0.544279	<.0001
P25	S00	131.713333	0.544279	<.0001
P25	S20	138.850000	0.544279	<.0001

P	S	TDN LSMEAN	Standard Error	Pr > |t|
P00	S00	733.790000	4.604499	<.0001
P00	S20	741.970000	4.604499	<.0001
P25	S00	700.523333	4.604499	<.0001

The GLM Procedure
Least Squares Means

P	S	TDN LSMEAN	Standard Error	Pr > |t|
P25	S20	723.011667	4.604499	<.0001

The MEANS Procedure

Variable	Std Dev
DM	23.7610289
OM	20.1619167
CP	30.4772782
EE	40.1000990
CF	52.5202399
NDF	44.9694081
ADF	62.6218725
NFE	10.9436465
NFC	17.8629872
DCP	6.0756903
TDN	19.0574166
