# Supplementary material for: Influence of substituting 25% alfalfa hay with Panicum maximum cv. Mombasa with or without spirulina supplementation on the productive performance of fattening Barki lambs
Source: Sci Rep. 2026 Jan 10;16:1347. doi: 10.1038/s41598-025-28525-1 (PMC12796356; doi:10.1038/s41598-025-28525-1)
Supplement: Supplementary file 1 — Supplementary Material 1 [file 41598_2025_28525_MOESM1_ESM.zip › Meteab_Supplementary/Raw Data/feed intake.pdf]

```
Data feed intake;
Input P$    S$    AW    BW    FoDM Fow   FoCP  FoCPw       TconDM
      Tconw TconCP      TconCPw    TDM   TDMwTCP     TCPw  TDN   TDNw
      DCP   DCPw;
Cards;
P00   S00   32.50 13.61 550.88 40.47 93.21 6.85  828.94 60.90 138.03 10.14
      1379.82     101.37 231.24 16.99 1032.50     75.85 198.92 14.61
P00   S00   33.00 13.77 550.88 40.01 93.21 6.77  828.94 60.21 138.03 10.03
      1379.82     100.22 231.24 16.79 1007.51     73.18 197.23 14.32
P00   S00   31.50 13.30 550.88 41.43 93.21 7.01  828.94 62.34 138.03 10.38
      1379.82     103.77 231.24 17.39 1012.68     76.16 196.10 14.75
P00   S00   39.75 15.83 550.88 34.80 93.21 5.89  828.94 52.36 138.03 8.72
      1379.82     87.16 231.24 14.61 1002.59      63.33 194.47 12.28
P00   S00   42.25 16.57 550.88 33.24 93.21 5.62  828.94 50.02 138.03 8.33
      1379.82     83.26 231.24 13.95 1017.07      61.37 197.14 11.90
P00   S00   37.00 15.00 550.88 36.72 93.21 6.21  828.94 55.26 138.03 9.20
      1379.82     91.98 231.24 15.41 1002.64      66.83 196.80 13.12
P00   S00   29.50 12.66 550.88 43.52 93.21 7.36  828.94 65.49 138.03 10.90
      1379.82     109.01 231.24 18.27 1012.50     79.99 196.78 15.55
P00   S00   34.50 14.24 550.88 38.70 93.21 6.55  828.94 58.23 138.03 9.70
      1379.82     96.93 231.24 16.24 1012.50      71.13 196.78 13.82
P00   S20   38.50 15.46 564.21 36.50 95.46 6.18  851.91 55.12 142.75 9.24
      1416.13     91.62 238.21 15.41 1026.72      66.43 207.19 13.41
P00   S20   34.25 14.16 564.21 39.85 95.46 6.74  851.91 60.17 142.75 10.08
      1416.13     100.02 238.21 16.83 1033.42     72.99 208.29 14.71
P00   S20   32.25 13.53 564.21 41.69 95.46 7.05  851.91 62.95 142.75 10.55
      1416.13     104.64 238.21 17.60 1032.81     76.32 207.32 15.32
P00   S20   41.00 16.20 564.21 34.82 95.46 5.89  851.91 52.58 142.75 8.81
      1416.13     87.40 238.21 14.70 1071.18      66.11 210.47 12.99
P00   S20   39.00 15.61 564.21 36.15 95.46 6.12  851.91 54.59 142.75 9.15
      1416.13     90.74 238.21 15.26 1057.33      67.75 209.35 13.41
P00   S20   39.50 15.76 564.21 35.81 95.46 6.06  851.91 54.07 142.75 9.06
      1416.13     89.88 238.21 15.12 1082.89      68.73 212.16 13.47
P00   S20   33.25 13.85 564.21 40.75 95.46 6.89  851.91 61.53 142.75 10.31
      1416.13     102.27 238.21 17.20 1050.72     75.88 209.13 15.10
P00   S20   33.25 13.85 564.21 40.75 95.46 6.89  851.91 61.53 142.75 10.31
      1416.13     102.27 238.21 17.20 1050.72     75.88 209.13 15.10
P25   S00   32.00 13.45 515.88 38.34 75.67 5.62  775.03 57.60 139.51 10.37
      1290.91     95.95 215.18 15.99 906.46 67.37 170.33 12.66
P25   S00   31.75 13.38 515.88 38.57 75.67 5.66  775.03 57.94 139.51 10.43
      1290.91     96.51 215.18 16.09 902.39 67.47 170.04 12.71
P25   S00   33.25 13.85 515.88 37.26 75.67 5.47  775.03 55.97 139.51 10.08
      1290.91     93.23 215.18 15.54 902.69 65.19 169.81 12.26
P25   S00   35.25 14.47 515.88 35.66 75.67 5.23  775.03 53.57 139.51 9.64
      1290.91     89.23 215.18 14.87 909.75 62.89 169.90 11.74
P25   S00   31.50 13.30 515.88 38.80 75.67 5.69  775.03 58.29 139.51 10.49
      1290.91     97.09 215.18 16.18 902.89 67.91 170.81 12.85
P25   S00   33.50 13.92 515.88 37.05 75.67 5.43  775.03 55.66 139.51 10.02
      1290.91     92.71 215.18 15.45 901.69 64.76 169.31 12.16
P25   S00   31.75 13.38 515.88 38.57 75.67 5.66  775.03 57.94 139.51 10.43
      1290.91     96.51 215.18 16.09 904.31 67.61 170.03 12.71
P25   S00   37.25 15.08 515.88 34.21 75.67 5.02  775.03 51.40 139.51 9.25
      1290.91     85.62 215.18 14.27 904.31 59.98 170.03 11.28
P25   S20   36.25 14.77 528.15 35.75 77.47 5.24  796.19 53.89 144.11 9.75
      1324.34     89.64 221.58 15.00 954.59 64.62 183.72 12.44
P25   S20   35.50 14.54 528.15 36.31 77.47 5.33  796.19 54.75 144.11 9.91
      1324.34     91.06 221.58 15.24 948.28 65.20 182.49 12.55
P25   S20   33.10 13.80 528.15 38.27 77.47 5.61  796.19 57.70 144.11 10.44
      1324.34     95.97 221.58 16.06 976.16 70.74 186.22 13.49
```

```
P25	S20	33.20	13.83	528.15 38.19	77.47	5.60	796.19 57.57	144.11 10.42
	1324.34		95.75	221.58 16.02	930.10 67.25	179.40 12.97
P25	S20	33.50	13.92	528.15 37.93	77.47	5.56	796.19 57.18	144.11 10.35
	1324.34		95.11	221.58 15.91	973.12 69.89	186.21 13.37
P25	S20	33.00	13.77	528.15 38.36	77.47	5.63	796.19 57.83	144.11 10.47
	1324.34		96.19	221.58 16.09	962.83 69.93	185.27 13.46
P25	S20	40.50	16.05	528.15 32.90	77.47	4.83	796.19 49.59	144.11 8.98
	1324.34		82.49	221.58 13.80	957.51 59.64	183.88 11.45
P25	S20	30.25	12.90	528.15 40.95	77.47	6.01	796.19 61.73	144.11 11.17
	1324.34		102.67 221.58 17.18	957.51 74.23	183.88 14.26
;
Proc GLM;
Class P S;
Model  AW	BW	FoDM Fow	FoCP	FoCPw		TconDM		Tconw TconCP
	TconCPw		TDM	TDMwTCP	TCPw  TDN	TDNw DCP	DCPw = P S P*S ;
MEANS P S / duncan;
LSMEANS P S P*S  / STDERR;
PROC MEANS STD; VAR  AW		BW	FoDM Fow	FoCP	FoCPw		TconDM
	Tconw TconCP		TconCPw		TDM	TDMwTCP		TCPw  TDN	TDNw
	DCP	DCPw;
RUN;
```
