# Supplementary material for: Influence of substituting 25% alfalfa hay with Panicum maximum cv. Mombasa with or without spirulina supplementation on the productive performance of fattening Barki lambs
Source: Sci Rep. 2026 Jan 10;16:1347. doi: 10.1038/s41598-025-28525-1 (PMC12796356; doi:10.1038/s41598-025-28525-1)
Supplement: Supplementary file 1 — Supplementary Material 1 [file 41598_2025_28525_MOESM1_ESM.zip › Meteab_Supplementary/Raw Data/Feedintake.pdf]

The GLM Procedure

Class Level Information

Class	Levels	Values
P	2	P00 P25
S	2	S00 S20

Number of observations 32

The GLM Procedure

Dependent Variable: AW

Source	DF	Sum of Squares	Mean Square	F Value	Pr > F
Model	3	39.7846094	13.2615365	1.20	0.3285
Error	28	309.8934375	11.0676228		
Corrected Total	31	349.6780469			

R-Square	Coeff Var	Root MSE	AW Mean
0.113775	9.568803	3.326804	34.76719

Source	DF	Type I SS	Mean Square	F Value	Pr > F
P	1	27.10320313	27.10320313	2.45	0.1288
S	1	12.56257813	12.56257813	1.14	0.2958
P*S	1	0.11882812	0.11882812	0.01	0.9182

Source	DF	Type III SS	Mean Square	F Value	Pr > F
P	1	27.10320313	27.10320313	2.45	0.1288
S	1	12.56257812	12.56257812	1.14	0.2958
P*S	1	0.11882813	0.11882813	0.01	0.9182

The GLM Procedure

Dependent Variable: BW

Source	DF	Sum of Squares	Mean Square	F Value	Pr > F
Model	3	3.73788437	1.24596146	1.20	0.3285
Error	28	29.11223750	1.03972277		
Corrected Total	31	32.85012188			

R-Square	Coeff Var	Root MSE	BW Mean
0.113786	7.127274	1.019668	14.30656

Source	DF	Type I SS	Mean Square	F Value	Pr > F
P	1	2.52562813	2.52562813	2.43	0.1303
S	1	1.19737812	1.19737812	1.15	0.2924
P*S	1	0.01487812	0.01487812	0.01	0.9056

Source	DF	Type III SS	Mean Square	F Value	Pr > F
P	1	2.52562813	2.52562813	2.43	0.1303
S	1	1.19737812	1.19737812	1.15	0.2924
P*S	1	0.01487813	0.01487813	0.01	0.9056

The GLM Procedure

Dependent Variable: FoDM

Source	DF	Sum of Squares	Mean Square	F Value	Pr > F
Model	3	11412.01440	3804.00480	Infty	<.0001
Error	28	0.00000	0.00000		
Corrected Total	31	11412.01440			

R-Square	Coeff Var	Root MSE	FoDM Mean
1.000000	0	0	539.7800

Source	DF	Type I SS	Mean Square	F Value	Pr > F
P	1	10099.04720	10099.04720	Infty	<.0001
S	1	1310.72000	1310.72000	Infty	<.0001
P*S	1	2.24720	2.24720	Infty	<.0001

Source	DF	Type III SS	Mean Square	F Value	Pr > F
P	1	10099.04720	10099.04720	Infty	<.0001
S	1	1310.72000	1310.72000	Infty	<.0001
P*S	1	2.24720	2.24720	Infty	<.0001

The GLM Procedure

Dependent Variable: Fow

Source	DF	Sum of Squares	Mean Square	F Value	Pr > F
Model	3	10.6418094	3.5472698	0.51	0.6785
Error	28	194.6539875	6.9519281		
Corrected Total	31	205.2957969			

R-Square	Coeff Var	Root MSE	Fow Mean
0.051836	6.959560	2.636651	37.88531

Source	DF	Type I SS	Mean Square	F Value	Pr > F
P	1	10.22650312	10.22650312	1.47	
S	1	0.17552812	0.17552812	0.03	
P*S	1	0.23977813	0.23977813	0.03	

Source	DF	Type III SS	Mean Square	F Value	Pr > F
P	1	10.22650313	10.22650313	1.47	0.2353
S	1	0.17552812	0.17552812	0.03	0.8749
P*S	1	0.23977813	0.23977813	0.03	0.8540

The GLM Procedure

Dependent Variable: FoCP

Source	DF	Sum of Squares	Mean Square	F Value	Pr > F
Model	3	2557.971800	852.657267	Infty	<.0001
Error	28	0.000000	0.000000		
Corrected Total	31	2557.971800			

R-Square	Coeff Var	Root MSE	FoCP Mean
1.000000	0	0	85.45250

Source	DF	Type I SS	Mean Square	F Value	Pr > F
P	1	2524.761800	2524.761800	Infty	<.0001
S	1	32.805000	32.805000	Infty	<.0001
P*S	1	0.405000	0.405000	Infty	<.0001

Source	DF	Type III SS	Mean Square	F Value	Pr > F
P	1	2524.761800	2524.761800	Infty	<.0001
S	1	32.805000	32.805000	Infty	<.0001
P*S	1	0.405000	0.405000	Infty	<.0001

The GLM Procedure

Dependent Variable: FoCPw

Source	DF	Sum of Squares	Mean Square	F Value	Pr > F
Model	3	8.50965937	2.83655312	15.45	<.0001
Error	28	5.14083750	0.18360134		
Corrected Total	31	13.65049688			

R-Square	Coeff Var	Root MSE	FoCPw Mean
0.623396	7.153750	0.428487	5.989688

Source	DF	Type I SS	Mean Square	F Value	Pr > F
P	1	8.49750312	8.49750312	46.28	<.0001
S	1	0.00525313	0.00525313	0.03	0.8669
P*S	1	0.00690313	0.00690313	0.04	0.8477

Source	DF	Type III SS	Mean Square	F Value	Pr > F
P	1	8.49750312	8.49750312	46.28	<.0001
S	1	0.00525312	0.00525312	0.03	0.8669
P*S	1	0.00690313	0.00690313	0.04	0.8477

The GLM Procedure

Dependent Variable: TconDM

Source	DF	Sum of Squares	Mean Square	F Value	Pr > F
Model	3	27938.93980	9312.97993	Infty	<.0001
Error	28	0.00000	0.00000		
Corrected Total	31	27938.93980			

R-Square	Coeff Var	Root MSE	TconDM Mean
1.000000	0	0	813.0175

Source	DF	Type I SS	Mean Square	F Value	Pr > F
P	1	24037.47380	24037.47380	Infty	<.0001
S	1	3894.91380	3894.91380	Infty	<.0001
P*S	1	6.55220	6.55220	Infty	<.0001

Source	DF	Type III SS	Mean Square	F Value	Pr > F
P	1	24037.47380	24037.47380	Infty	<.0001
S	1	3894.91380	3894.91380	Infty	<.0001
P*S	1	6.55220	6.55220	Infty	<.0001

The GLM Procedure

Dependent Variable: Tconw

Source	DF	Sum of Squares	Mean Square	F Value	Pr > F
Model	3	26.3527250	8.7842417	0.56	0.6479
Error	28	441.7090250	15.7753223		
Corrected Total	31	468.0617500			

R-Square	Coeff Var	Root MSE	Tconw Mean
0.056302	6.960619	3.971816	57.06125

Source	DF	Type I SS	Mean Square	F Value	Pr > F
P	1	25.81211250	25.81211250	1.64	0.2113
S	1	0.00500000	0.00500000	0.00	0.9859
P*S	1	0.53561250	0.53561250	0.03	0.8551

Source	DF	Type III SS	Mean Square	F Value	Pr > F
P	1	25.81211250	25.81211250	1.64	0.2113
S	1	0.00500000	0.00500000	0.00	0.9859
P*S	1	0.53561250	0.53561250	0.03	0.8551

The GLM Procedure

Dependent Variable: TconCP

Source	DF	Sum of Squares	Mean Square	F Value	Pr > F
Model	3	189.8848000	63.2949333	Infty	<.0001
Error	28	0.0000000	0.0000000		
Corrected Total	31	189.8848000			

R-Square	Coeff Var	Root MSE	TconCP Mean
1.000000	0	0	141.1000

Source	DF	Type I SS	Mean Square	F Value	Pr > F
P	1	16.1312000	16.1312000	Infty	<.0001
S	1	173.7248000	173.7248000	Infty	<.0001
P*S	1	0.0288000	0.0288000	Infty	<.0001

Source	DF	Type III SS	Mean Square	F Value	Pr > F
P	1	16.1312000	16.1312000	Infty	<.0001
S	1	173.7248000	173.7248000	Infty	<.0001
P*S	1	0.0288000	0.0288000	Infty	<.0001

The GLM Procedure

Dependent Variable: TconCPw

Source	DF	Sum of Squares	Mean Square	F Value	Pr > F
Model	3	1.69953437	0.56651146	1.23	0.3178
Error	28	12.91156250	0.46112723		
Corrected Total	31	14.61109688			

R-Square	Coeff Var	Root MSE	TconCPw Mean
0.116318	6.852522	0.679063	9.909688

Source	DF	Type I SS	Mean Square	F Value	Pr > F
P	1	1.66075312	1.66075312	3.60	0.0681
S	1	0.02475313	0.02475313	0.05	0.8185
P*S	1	0.01402812	0.01402812	0.03	0.8628

Source	DF	Type III SS	Mean Square	F Value	Pr > F
P	1	1.66075312	1.66075312	3.60	0.0681
S	1	0.02475313	0.02475313	0.05	0.8185
P*S	1	0.01402812	0.01402812	0.03	0.8628

The GLM Procedure

Dependent Variable: TDM

Source	DF	Sum of Squares	Mean Square	F Value	Pr > F
Model	3	0.08220000	0.02740000	Infty	<.0001
Error	28	0.00000000	0.00000000		
Corrected Total	31	0.08220000			

R-Square	Coeff Var	Root MSE	TDM Mean
1.000000	0	0	1.352500

Source	DF	Type I SS	Mean Square	F Value	Pr > F
P	1	0.07220000	0.07220000	Infty	<.0001
S	1	0.00980000	0.00980000	Infty	<.0001
P*S	1	0.00020000	0.00020000	Infty	<.0001

Source	DF	Type III SS	Mean Square	F Value	Pr > F
P	1	0.07220000	0.07220000	Infty	<.0001
S	1	0.00980000	0.00980000	Infty	<.0001
P*S	1	0.00020000	0.00020000	Infty	<.0001

The GLM Procedure

Dependent Variable: TDMw

Source	DF	Sum of Squares	Mean Square	F Value	Pr > F
Model	3	70.208034	23.402678	0.54	0.6614
Error	28	1222.236338	43.651298		
Corrected Total	31	1292.444372			

R-Square	Coeff Var	Root MSE	TDMw Mean
0.054322	6.958605	6.606913	94.94594

Source	DF	Type I SS	Mean Square	F Value	Pr > F
P	1	68.47425312	68.47425312	1.57	0.2208
S	1	0.25027813	0.25027813	0.01	0.9402
P*S	1	1.48350312	1.48350312	0.03	0.8551

Source	DF	Type III SS	Mean Square	F Value	Pr > F
P	1	68.47425312	68.47425312	1.57	0.2208
S	1	0.25027813	0.25027813	0.01	0.9402
P*S	1	1.48350313	1.48350313	0.03	0.8551

The GLM Procedure

Dependent Variable: TCP

Source	DF	Sum of Squares	Mean Square	F Value	Pr > F
Model	3	2495.435800	831.811933	Infty	<.0001
Error	28	0.000000	0.000000		
Corrected Total	31	2495.435800			

R-Square	Coeff Var	Root MSE	TCP Mean
1.000000	0	0	226.5525

Source	DF	Type I SS	Mean Square	F Value	Pr > F
P	1	2137.272200	2137.272200	Infty	<.0001
S	1	357.513800	357.513800	Infty	<.0001
P*S	1	0.649800	0.649800	Infty	<.0001

Source	DF	Type III SS	Mean Square	F Value	Pr > F
P	1	2137.272200	2137.272200	Infty	<.0001
S	1	357.513800	357.513800	Infty	<.0001
P*S	1	0.649800	0.649800	Infty	<.0001

The GLM Procedure

Dependent Variable: TCPw

Source	DF	Sum of Squares	Mean Square	F Value	Pr > F
Model	3	2.68808437	0.89602812	0.73	0.5427
Error	28	34.36313750	1.22725491		
Corrected Total	31	37.05122188			

R-Square	Coeff Var	Root MSE	TCPw Mean
0.072550	6.968077	1.107815	15.89844

Source	DF	Type I SS	Mean Square	F Value	Pr > F
P	1	2.63925312	2.63925312	2.15	0.1537
S	1	0.00750312	0.00750312	0.01	0.9382
P*S	1	0.04132812	0.04132812	0.03	0.8557

Source	DF	Type III SS	Mean Square	F Value	Pr > F
P	1	2.63925312	2.63925312	2.15	0.1537
S	1	0.00750313	0.00750313	0.01	0.9382
P*S	1	0.04132812	0.04132812	0.03	0.8557

The GLM Procedure

Dependent Variable: TDN

Source	DF	Sum of Squares	Mean Square	F Value	Pr > F
Model	3	0.09910938	0.03303646	185.47	<.0001
Error	28	0.00498750	0.00017813		
Corrected Total	31	0.10409688			

R-Square	Coeff Var	Root MSE	TDN Mean
0.952088	1.361438	0.013346	0.980313

Source	DF	Type I SS	Mean Square	F Value	Pr > F
P	1	0.08100313	0.08100313	454.75	<.0001
S	1	0.01757812	0.01757812	98.68	<.0001
P*S	1	0.00052812	0.00052812	2.96	0.0961

Source	DF	Type III SS	Mean Square	F Value	Pr > F
P	1	0.08100313	0.08100313	454.75	<.0001
S	1	0.01757812	0.01757812	98.68	<.0001
P*S	1	0.00052812	0.00052812	2.96	0.0961

The GLM Procedure

Dependent Variable: TDNw

Source	DF	Sum of Squares	Mean Square	F Value	Pr > F
Model	3	188.9666344	62.9888781	2.74	0.0617
Error	28	642.5795875	22.9492710		
Corrected Total	31	831.5462219			

R-Square	Coeff Var	Root MSE	TDNw Mean
0.227247	6.959801	4.790540	68.83156

Source	DF	Type I SS	Mean Square	F Value	Pr > F
P	1	167.6738281	167.6738281	7.31	0.0115
S	1	13.2226531	13.2226531	0.58	0.4542
P*S	1	8.0701531	8.0701531	0.35	0.5579

Source	DF	Type III SS	Mean Square	F Value	Pr > F
P	1	167.6738281	167.6738281	7.31	0.0115
S	1	13.2226531	13.2226531	0.58	0.4542
P*S	1	8.0701531	8.0701531	0.35	0.5579

The GLM Procedure

Dependent Variable: DCP

Source	DF	Sum of Squares	Mean Square	F Value	Pr > F
Model	3	6783.945684	2261.315228	964.79	<.0001
Error	28	65.627488	2.343839		
Corrected Total	31	6849.573172			

R-Square	Coeff Var	Root MSE	DCP Mean
0.990419	0.805955	1.530960	189.9559

Source	DF	Type I SS	Mean Square	F Value	Pr > F
P	1	5406.180153	5406.180153	2306.55	<.0001
S	1	1373.273028	1373.273028	585.91	<.0001
P*S	1	4.492503	4.492503	1.92	0.1772

Source	DF	Type III SS	Mean Square	F Value	Pr > F
P	1	5406.180153	5406.180153	2306.55	<.0001
S	1	1373.273028	1373.273028	585.91	<.0001
P*S	1	4.492503	4.492503	1.92	0.1772

The GLM Procedure

Dependent Variable: DCPw

Source	DF	Sum of Squares	Mean Square	F Value	Pr > F
Model	3	17.04343750	5.68114583	6.39	0.0019
Error	28	24.89215000	0.88900536		
Corrected Total	31	41.93558750			

R-Square	Coeff Var	Root MSE	DCPw Mean
0.406419	7.078942	0.942871	13.31938

Source	DF	Type I SS	Mean Square	F Value	Pr > F
P	1	14.44531250	14.44531250	16.25	0.0004
S	1	2.40901250	2.40901250	2.71	0.1109
P*S	1	0.18911250	0.18911250	0.21	0.6482

Source	DF	Type III SS	Mean Square	F Value	Pr > F
P	1	14.44531250	14.44531250	16.25	0.0004
S	1	2.40901250	2.40901250	2.71	0.1109
P*S	1	0.18911250	0.18911250	0.21	0.6482

The GLM Procedure

Dependent Variable: Initial

Source	DF	Sum of Squares	Mean Square	F Value	Pr > F
Model	3	0.0000000	0.0000000	0.00	1.0000
Error	28	323.5000000	11.5535714		
Corrected Total	31	323.5000000			

R-Square	Coeff Var	Root MSE	Initial Mean
0.000000	15.71817	3.399054	21.62500

Source	DF	Type I SS	Mean Square	F Value	Pr > F
P	1	0	0	0.00	1.0000
S	1	0	0	0.00	1.0000
P*S	1	0	0	0.00	1.0000

Source	DF	Type III SS	Mean Square	F Value	Pr > F
P	1	0	0	0.00	1.0000
S	1	0	0	0.00	1.0000
P*S	1	0	0	0.00	1.0000

The GLM Procedure

Dependent Variable: Final

Source	DF	Sum of Squares	Mean Square	F Value	Pr > F
Model	3	159.1384375	53.0461458	4.06	0.0163
Error	28	365.7487500	13.0624554		
Corrected Total	31	524.8871875			

R-Square	Coeff Var	Root MSE	Final Mean
0.303186	7.543830	3.614202	47.90938

Source	DF	Type I SS	Mean Square	F Value	Pr > F
P	1	108.4128125	108.4128125	8.30	0.0075
S	1	50.2503125	50.2503125	3.85	0.0499
P*S	1	0.4753125	0.4753125	0.04	0.8501

Source	DF	Type III SS	Mean Square	F Value	Pr > F
P	1	108.4128125	108.4128125	8.30	0.0075
S	1	50.2503125	50.2503125	3.85	0.0499
P*S	1	0.4753125	0.4753125	0.04	0.8501

The GLM Procedure

Dependent Variable: TGA

Source	DF	Sum of Squares	Mean Square	F Value	Pr > F
Model	3	159.1384375	53.0461458	10.69	<.0001
Error	28	138.9237500	4.9615625		
Corrected Total	31	298.0621875			

R-Square	Coeff Var	Root MSE	TGA Mean
0.533910	8.474451	2.227457	26.28438

Source	DF	Type I SS	Mean Square	F Value	Pr > F
P	1	108.4128125	108.4128125	21.85	<.0001
S	1	50.2503125	50.2503125	10.13	0.0036
P*S	1	0.4753125	0.4753125	0.10	0.7592

Source	DF	Type III SS	Mean Square	F Value	Pr > F
P	1	108.4128125	108.4128125	21.85	<.0001
S	1	50.2503125	50.2503125	10.13	0.0036
P*S	1	0.4753125	0.4753125	0.10	0.7592

The GLM Procedure

Dependent Variable: ADG

Source	DF	Sum of Squares	Mean Square	F Value	Pr > F
Model	3	0.01135938	0.00378646	11.35	<.0001
Error	28	0.00933750	0.00033348		
Corrected Total	31	0.02069688			

R-Square	Coeff Var	Root MSE	ADG Mean
0.548845	8.288905	0.018261	0.220313

Source	DF	Type I SS	Mean Square	F Value	Pr > F
P	1	0.00750313	0.00750313	22.50	<.0001
S	1	0.00382812	0.00382812	11.48	0.0021
P*S	1	0.00002813	0.00002813	0.08	0.7736

Source	DF	Type III SS	Mean Square	F Value	Pr > F
P	1	0.00750313	0.00750313	22.50	<.0001
S	1	0.00382812	0.00382812	11.48	0.0021
P*S	1	0.00002813	0.00002813	0.08	0.7736

The GLM Procedure

Dependent Variable: ADG

Source	DF	Sum of Squares	Mean Square	F Value	Pr > F
Model	3	0.01135938	0.00378646	11.35	<.0001
Error	28	0.00933750	0.00033348		
Corrected Total	31	0.02069688			

R-Square	Coeff Var	Root MSE	ADG Mean
0.548845	8.288905	0.018261	0.220313

Source	DF	Type I SS	Mean Square	F Value	Pr > F
P	1	0.00750313	0.00750313	22.50	<.0001
S	1	0.00382812	0.00382812	11.48	0.0021
P*S	1	0.00002813	0.00002813	0.08	0.7736

Source	DF	Type III SS	Mean Square	F Value	Pr > F
P	1	0.00750313	0.00750313	22.50	<.0001
S	1	0.00382812	0.00382812	11.48	0.0021
P*S	1	0.00002813	0.00002813	0.08	0.7736

The GLM Procedure

Dependent Variable: TDM

Source	DF	Sum of Squares	Mean Square	F Value	Pr > F
Model	3	0.08220000	0.02740000	Infty	<.0001
Error	28	0.00000000	0.00000000		
Corrected Total	31	0.08220000			

R-Square	Coeff Var	Root MSE	TDM Mean
1.000000	0	0	1.352500

Source	DF	Type I SS	Mean Square	F Value	Pr > F
P	1	0.07220000	0.07220000	Infty	<.0001
S	1	0.00980000	0.00980000	Infty	<.0001
P*S	1	0.00020000	0.00020000	Infty	<.0001

Source	DF	Type III SS	Mean Square	F Value	Pr > F
P	1	0.07220000	0.07220000	Infty	<.0001
S	1	0.00980000	0.00980000	Infty	<.0001
P*S	1	0.00020000	0.00020000	Infty	<.0001

The GLM Procedure

Dependent Variable: DMC

Source	DF	Sum of Squares	Mean Square	F Value	Pr > F
Model	3	3.43750937	1.14583646	3.77	0.0217
Error	28	8.51686250	0.30417366		
Corrected Total	31	11.95437188			

R-Square	Coeff Var	Root MSE	DMC Mean
0.287552	8.832702	0.551519	6.244063

Source	DF	Type I SS	Mean Square	F Value	Pr > F
P	1	1.94537812	1.94537812	6.40	0.0174
S	1	1.49212813	1.49212813	4.91	0.0351
P*S	1	0.00000312	0.00000312	0.00	0.9975

Source	DF	Type III SS	Mean Square	F Value	Pr > F
P	1	1.94537812	1.94537812	6.40	0.0174
S	1	1.49212813	1.49212813	4.91	0.0351
P*S	1	0.00000313	0.00000313	0.00	0.9975

The GLM Procedure

Dependent Variable: TDN

Source	DF	Sum of Squares	Mean Square	F Value	Pr > F
Model	3	0.09910938	0.03303646	185.47	<.0001
Error	28	0.00498750	0.00017813		
Corrected Total	31	0.10409688			

R-Square	Coeff Var	Root MSE	TDN Mean
0.952088	1.361438	0.013346	0.980313

Source	DF	Type I SS	Mean Square	F Value	Pr > F
P	1	0.08100313	0.08100313	454.75	<.0001
S	1	0.01757812	0.01757812	98.68	<.0001
P*S	1	0.00052812	0.00052812	2.96	0.0961

Source	DF	Type III SS	Mean Square	F Value	Pr > F
P	1	0.08100313	0.08100313	454.75	<.0001
S	1	0.01757812	0.01757812	98.68	<.0001
P*S	1	0.00052812	0.00052812	2.96	0.0961

The GLM Procedure

Dependent Variable: TDNC

Source	DF	Sum of Squares	Mean Square	F Value	Pr > F
Model	3	0.70457500	0.23485833	1.56	0.2218
Error	28	4.22262500	0.15080804		
Corrected Total	31	4.92720000			

R-Square	Coeff Var	Root MSE	TDNC Mean
0.142997	8.591595	0.388340	4.520000

Source	DF	Type I SS	Mean Square	F Value	Pr > F
P	1	0.29645000	0.29645000	1.97	0.1719
S	1	0.38281250	0.38281250	2.54	0.1223
P*S	1	0.02531250	0.02531250	0.17	0.6852

Source	DF	Type III SS	Mean Square	F Value	Pr > F
P	1	0.29645000	0.29645000	1.97	0.1719
S	1	0.38281250	0.38281250	2.54	0.1223
P*S	1	0.02531250	0.02531250	0.17	0.6852

The GLM Procedure

Dependent Variable: DCPC

Source	DF	Sum of Squares	Mean Square	F Value	Pr > F
Model	3	5146.5576	1715.5192	0.30	0.8230
Error	28	158566.9773	5663.1063		
Corrected Total	31	163713.5349			

R-Square	Coeff Var	Root MSE	DCPC Mean
0.031436	8.614553	75.25361	873.5638

Source	DF	Type I SS	Mean Square	F Value	Pr > F
P	1	370.328112	370.328112	0.07	0.8000
S	1	3952.049513	3952.049513	0.70	0.4106
P*S	1	824.180000	824.180000	0.15	0.7057

Source	DF	Type III SS	Mean Square	F Value	Pr > F
P	1	370.328112	370.328112	0.07	0.8000
S	1	3952.049513	3952.049513	0.70	0.4106
P*S	1	824.180000	824.180000	0.15	0.7057

The GLM Procedure

Dependent Variable: CPC

Source	DF	Sum of Squares	Mean Square	F Value	Pr > F
Model	3	84105.7994	28035.2665	3.30	0.0348
Error	28	237950.8228	8498.2437		
Corrected Total	31	322056.6222			

R-Square	Coeff Var	Root MSE	CPC Mean
0.261152	8.818122	92.18592	1045.414

Source	DF	Type I SS	Mean Square	F Value	Pr > F
P	1	46795.05281	46795.05281	5.51	0.0263
S	1	37308.19280	37308.19280	4.39	0.0453
P*S	1	2.55380	2.55380	0.00	0.9863

Source	DF	Type III SS	Mean Square	F Value	Pr > F
P	1	46795.05281	46795.05281	5.51	0.0263
S	1	37308.19280	37308.19280	4.39	0.0453
P*S	1	2.55380	2.55380	0.00	0.9863

The GLM Procedure

Duncan's Multiple Range Test for AW

NOTE: This test controls the Type I comparisonwise error rate, not the experimentwise error rate.

Alpha	0.05
Error Degrees of Freedom	28
Error Mean Square	11.06762

Number of Means	2
Critical Range	2.409

Means with the same letter are not significantly different.

Duncan Grouping	Mean	N	P
A	35.688	16	P00
A			
A	33.847	16	P25

The GLM Procedure

Duncan's Multiple Range Test for BW

NOTE: This test controls the Type I comparisonwise error rate, not the experimentwise error rate.

Alpha	0.05
Error Degrees of Freedom	28
Error Mean Square	1.039723

Number of Means	2
Critical Range	.7385

Means with the same letter are not significantly different.

Duncan Grouping	Mean	N	P
A	14.5875	16	P00
A			
A	14.0256	16	P25

The GLM Procedure

Duncan's Multiple Range Test for FoDM

NOTE: This test controls the Type I comparisonwise error rate, not the experimentwise error rate.

Alpha	0.05
Error Degrees of Freedom	28
Error Mean Square	0

Number of Means	2
Critical Range	0

Means with the same letter are not significantly different.

Duncan Grouping	Mean	N	P
A	557.5	16	P00
B	522.0	16	P25

The GLM Procedure

Duncan's Multiple Range Test for Fow

NOTE: This test controls the Type I comparisonwise error rate, not the experimentwise error rate.

Alpha	0.05
Error Degrees of Freedom	28
Error Mean Square	6.951928

Number of Means	2
Critical Range	1.910

Means with the same letter are not significantly different.

Duncan Grouping	Mean	N	P
A	38.4506	16	P00
A			
A	37.3200	16	P25

The GLM Procedure

Duncan's Multiple Range Test for FoCP

NOTE: This test controls the Type I comparisonwise error rate, not the experimentwise error rate.

Alpha	0.05
Error Degrees of Freedom	28
Error Mean Square	0

Number of Means	2
Critical Range	0

Means with the same letter are not significantly different.

Duncan Grouping	Mean	N	P
A	94.34	16	P00
B	76.57	16	P25

The GLM Procedure

Duncan's Multiple Range Test for FoCPw

NOTE: This test controls the Type I comparisonwise error rate, not the experimentwise error rate.

Alpha	0.05
Error Degrees of Freedom	28
Error Mean Square	0.183601

Number of Means	2
Critical Range	.3103

Means with the same letter are not significantly different.

Duncan Grouping	Mean	N	P
A	6.5050	16	P00
B	5.4744	16	P25

The GLM Procedure

Duncan's Multiple Range Test for TconDM

NOTE: This test controls the Type I comparisonwise error rate, not the experimentwise error rate.

Alpha	0.05
Error Degrees of Freedom	28
Error Mean Square	0

Number of Means	2
Critical Range	0

Means with the same letter are not significantly different.

Duncan Grouping	Mean	N	P
A	840.4	16	P00
B	785.6	16	P25

The GLM Procedure

Duncan's Multiple Range Test for Tconw

NOTE: This test controls the Type I comparisonwise error rate, not the experimentwise error rate.

Alpha	0.05
Error Degrees of Freedom	28
Error Mean Square	15.77532

Number of Means	2
Critical Range	2.876

Means with the same letter are not significantly different.

Duncan Grouping	Mean	N	P
A	57.959	16	P00
A			
A	56.163	16	P25

The GLM Procedure

Duncan's Multiple Range Test for TconCP

NOTE: This test controls the Type I comparisonwise error rate, not the experimentwise error rate.

Alpha	0.05
Error Degrees of Freedom	28
Error Mean Square	0

Number of Means	2
Critical Range	0

Means with the same letter are not significantly different.

Duncan Grouping	Mean	N	P
A	141.8	16	P25
B	140.4	16	P00

The GLM Procedure

Duncan's Multiple Range Test for TconCPw

NOTE: This test controls the Type I comparisonwise error rate, not the experimentwise error rate.

Alpha	0.05
Error Degrees of Freedom	28
Error Mean Square	0.461127

Number of Means	2
Critical Range	.4918

Means with the same letter are not significantly different.

Duncan Grouping	Mean	N	P
A	10.1375	16	P25
A			
A	9.6819	16	P00

The GLM Procedure

Duncan's Multiple Range Test for TDM

NOTE: This test controls the Type I comparisonwise error rate, not the experimentwise error rate.

Alpha	0.05
Error Degrees of Freedom	28
Error Mean Square	0

Number of Means	2
Critical Range	0

Means with the same letter are not significantly different.

Duncan Grouping	Mean	N	P
A	1.400	16	P00
B	1.305	16	P25

The GLM Procedure

Duncan's Multiple Range Test for TDMw

NOTE: This test controls the Type I comparisonwise error rate, not the experimentwise error rate.

Alpha	0.05
Error Degrees of Freedom	28
Error Mean Square	43.6513

Number of Means	2
Critical Range	4.785

Means with the same letter are not significantly different.

Duncan Grouping	Mean	N	P
A	96.409	16	P00
A			
A	93.483	16	P25

The GLM Procedure

Duncan's Multiple Range Test for TCP

NOTE: This test controls the Type I comparisonwise error rate, not the experimentwise error rate.

Alpha	0.05
Error Degrees of Freedom	28
Error Mean Square	0

Number of Means	2
Critical Range	0

Means with the same letter are not significantly different.

Duncan Grouping	Mean	N	P
A	234.7	16	P00
B	218.4	16	P25

The GLM Procedure

Duncan's Multiple Range Test for TCPw

NOTE: This test controls the Type I comparisonwise error rate, not the experimentwise error rate.

Alpha	0.05
Error Degrees of Freedom	28
Error Mean Square	1.227255

Number of Means	2
Critical Range	.8023

Means with the same letter are not significantly different.

Duncan Grouping	Mean	N	P
A	16.1856	16	P00
A			
A	15.6113	16	P25

The GLM Procedure

Duncan's Multiple Range Test for TDN

NOTE: This test controls the Type I comparisonwise error rate, not the experimentwise error rate.

Alpha	0.05
Error Degrees of Freedom	28
Error Mean Square	0.000178

Number of Means	2
Critical Range	.009666

Means with the same letter are not significantly different.

Duncan Grouping	Mean	N	P
A	1.030625	16	P00
B	0.930000	16	P25

The GLM Procedure

Duncan's Multiple Range Test for TDNw

NOTE: This test controls the Type I comparisonwise error rate, not the experimentwise error rate.

Alpha	0.05
Error Degrees of Freedom	28
Error Mean Square	22.94927

Number of Means	2
Critical Range	3.469

Means with the same letter are not significantly different.

Duncan Grouping	Mean	N	P
A	71.121	16	P00
B	66.543	16	P25

The GLM Procedure

Duncan's Multiple Range Test for DCP

NOTE: This test controls the Type I comparisonwise error rate, not the experimentwise error rate.

Alpha	0.05
Error Degrees of Freedom	28
Error Mean Square	2.343839

Number of Means	2
Critical Range	1.109

Means with the same letter are not significantly different.

Duncan Grouping	Mean	N	P
A	202.9538	16	P00
B	176.9581	16	P25

The GLM Procedure

Duncan's Multiple Range Test for DCPw

NOTE: This test controls the Type I comparisonwise error rate, not the experimentwise error rate.

Alpha	0.05
Error Degrees of Freedom	28
Error Mean Square	0.889005

Number of Means	2
Critical Range	.6828

Means with the same letter are not significantly different.

Duncan Grouping	Mean	N	P
A	13.9913	16	P00
B	12.6475	16	P25

The GLM Procedure

Duncan's Multiple Range Test for Initial

NOTE: This test controls the Type I comparisonwise error rate, not the experimentwise error rate.

Alpha	0.05
Error Degrees of Freedom	28
Error Mean Square	11.55357

Number of Means	2
Critical Range	2.462

Means with the same letter are not significantly different.

Duncan Grouping	Mean	N	P
A	21.625	16	P00
A			
A	21.625	16	P25

The GLM Procedure

Duncan's Multiple Range Test for Final

NOTE: This test controls the Type I comparisonwise error rate, not the experimentwise error rate.

Alpha	0.05
Error Degrees of Freedom	28
Error Mean Square	13.06246

Number of Means	2
Critical Range	2.617

Means with the same letter are not significantly different.

Duncan Grouping	Mean	N	P
A	49.750	16	P00
B	46.069	16	P25

The GLM Procedure

Duncan's Multiple Range Test for TGA

NOTE: This test controls the Type I comparisonwise error rate, not the experimentwise error rate.

Alpha	0.05
Error Degrees of Freedom	28
Error Mean Square	4.961562

Number of Means	2
Critical Range	1.613

Means with the same letter are not significantly different.

Duncan Grouping	Mean	N	P
A	28.1250	16	P00
B	24.4438	16	P25

The GLM Procedure

Duncan's Multiple Range Test for ADG

NOTE: This test controls the Type I comparisonwise error rate, not the experimentwise error rate.

Alpha	0.05
Error Degrees of Freedom	28
Error Mean Square	0.000333

Number of Means	2
Critical Range	.01323

Means with the same letter are not significantly different.

Duncan Grouping	Mean	N	P
A	0.235625	16	P00
B	0.205000	16	P25

The GLM Procedure

Duncan's Multiple Range Test for ADG

NOTE: This test controls the Type I comparisonwise error rate, not the experimentwise error rate.

Alpha	0.05
Error Degrees of Freedom	28
Error Mean Square	0.000333

Number of Means	2
Critical Range	.01323

Means with the same letter are not significantly different.

Duncan Grouping	Mean	N	P
A	0.235625	16	P00
B	0.205000	16	P25

The GLM Procedure

Duncan's Multiple Range Test for TDM

NOTE: This test controls the Type I comparisonwise error rate, not the experimentwise error rate.

Alpha	0.05
Error Degrees of Freedom	28
Error Mean Square	0

Number of Means	2
Critical Range	0

Means with the same letter are not significantly different.

Duncan Grouping	Mean	N	P
A	1.400	16	P00
B	1.305	16	P25

The GLM Procedure

Duncan's Multiple Range Test for DMC

NOTE: This test controls the Type I comparisonwise error rate, not the experimentwise error rate.

Alpha	0.05
Error Degrees of Freedom	28
Error Mean Square	0.304174

Number of Means	2
Critical Range	.3994

Means with the same letter are not significantly different.

Duncan Grouping	Mean	N	P
A	6.4906	16	P25
B	5.9975	16	P00

The GLM Procedure

Duncan's Multiple Range Test for TDN

NOTE: This test controls the Type I comparisonwise error rate, not the experimentwise error rate.

Alpha	0.05
Error Degrees of Freedom	28
Error Mean Square	0.000178

Number of Means	2
Critical Range	.009666

Means with the same letter are not significantly different.

Duncan Grouping	Mean	N	P
A	1.030625	16	P00
B	0.930000	16	P25

The GLM Procedure

Duncan's Multiple Range Test for TDNC

NOTE: This test controls the Type I comparisonwise error rate, not the experimentwise error rate.

Alpha	0.05
Error Degrees of Freedom	28
Error Mean Square	0.150808

Number of Means	2
Critical Range	.2812

Means with the same letter are not significantly different.

Duncan Grouping	Mean	N	P
A	4.6162	16	P25
A			
A	4.4238	16	P00

The GLM Procedure

Duncan's Multiple Range Test for DCPC

NOTE: This test controls the Type I comparisonwise error rate, not the experimentwise error rate.

Alpha	0.05
Error Degrees of Freedom	28
Error Mean Square	5663.106

Number of Means	2
Critical Range	54.50

Means with the same letter are not significantly different.

Duncan Grouping	Mean	N	P
A	876.97	16	P25
A			
A	870.16	16	P00

The GLM Procedure

Duncan's Multiple Range Test for CPC

NOTE: This test controls the Type I comparisonwise error rate, not the experimentwise error rate.

Alpha	0.05
Error Degrees of Freedom	28
Error Mean Square	8498.244

Number of Means	2
Critical Range	66.76

Means with the same letter are not significantly different.

Duncan Grouping	Mean	N	P
A	1083.66	16	P25
B	1007.17	16	P00

The GLM Procedure

Duncan's Multiple Range Test for AW

NOTE: This test controls the Type I comparisonwise error rate, not the experimentwise error rate.

Alpha	0.05
Error Degrees of Freedom	28
Error Mean Square	11.06762

Number of Means	2
Critical Range	2.409

Means with the same letter are not significantly different.

Duncan Grouping	Mean	N	S
A	35.394	16	S20
A			
A	34.141	16	S00

The GLM Procedure

Duncan's Multiple Range Test for BW

NOTE: This test controls the Type I comparisonwise error rate, not the experimentwise error rate.

Alpha	0.05
Error Degrees of Freedom	28
Error Mean Square	1.039723

Number of Means	2
Critical Range	.7385

Means with the same letter are not significantly different.

Duncan Grouping	Mean	N	S
A	14.5000	16	S20
A			
A	14.1131	16	S00

The GLM Procedure

Duncan's Multiple Range Test for FoDM

NOTE: This test controls the Type I comparisonwise error rate, not the experimentwise error rate.

Alpha	0.05
Error Degrees of Freedom	28
Error Mean Square	0

Number of Means	2
Critical Range	0

Means with the same letter are not significantly different.

Duncan Grouping	Mean	N	S
A	546.2	16	S20
B	533.4	16	S00

The GLM Procedure

Duncan's Multiple Range Test for Fow

NOTE: This test controls the Type I comparisonwise error rate, not the experimentwise error rate.

Alpha	0.05
Error Degrees of Freedom	28
Error Mean Square	6.951928

Number of Means	2
Critical Range	1.910

Means with the same letter are not significantly different.

Duncan Grouping	Mean	N	S
A	37.9594	16	S00
A			
A	37.8113	16	S20

The GLM Procedure

Duncan's Multiple Range Test for FoCP

NOTE: This test controls the Type I comparisonwise error rate, not the experimentwise error rate.

Alpha	0.05
Error Degrees of Freedom	28
Error Mean Square	0

Number of Means	2
Critical Range	0

Means with the same letter are not significantly different.

Duncan Grouping	Mean	N	S
A	86.47	16	S20
B	84.44	16	S00

The GLM Procedure

Duncan's Multiple Range Test for FoCPw

NOTE: This test controls the Type I comparisonwise error rate, not the experimentwise error rate.

Alpha	0.05
Error Degrees of Freedom	28
Error Mean Square	0.183601

Number of Means	2
Critical Range	.3103

Means with the same letter are not significantly different.

Duncan Grouping	Mean	N	S
A	6.0025	16	S00
A			
A	5.9769	16	S20

The GLM Procedure

Duncan's Multiple Range Test for TconDM

NOTE: This test controls the Type I comparisonwise error rate, not the experimentwise error rate.

Alpha	0.05
Error Degrees of Freedom	28
Error Mean Square	0

Number of Means	2
Critical Range	0

Means with the same letter are not significantly different.

Duncan Grouping	Mean	N	S
A	824.1	16	S20
B	802.0	16	S00

The GLM Procedure

Duncan's Multiple Range Test for Tconw

NOTE: This test controls the Type I comparisonwise error rate, not the experimentwise error rate.

Alpha	0.05
Error Degrees of Freedom	28
Error Mean Square	15.77532

Number of Means	2
Critical Range	2.876

Means with the same letter are not significantly different.

Duncan Grouping	Mean	N	S
A	57.074	16	S00
A			
A	57.049	16	S20

The GLM Procedure

Duncan's Multiple Range Test for TconCP

NOTE: This test controls the Type I comparisonwise error rate, not the experimentwise error rate.

Alpha	0.05
Error Degrees of Freedom	28
Error Mean Square	0

Number of Means	2
Critical Range	0

Means with the same letter are not significantly different.

Duncan Grouping	Mean	N	S
A	143.4	16	S20
B	138.8	16	S00

The GLM Procedure

Duncan's Multiple Range Test for TconCPw

NOTE: This test controls the Type I comparisonwise error rate, not the experimentwise error rate.

Alpha	0.05
Error Degrees of Freedom	28
Error Mean Square	0.461127

Number of Means	2
Critical Range	.4918

Means with the same letter are not significantly different.

Duncan Grouping	Mean	N	S
A	9.9375	16	S20
A			
A	9.8819	16	S00

The GLM Procedure

Duncan's Multiple Range Test for TDM

NOTE: This test controls the Type I comparisonwise error rate, not the experimentwise error rate.

Alpha	0.05
Error Degrees of Freedom	28
Error Mean Square	0

Number of Means	2
Critical Range	0

Means with the same letter are not significantly different.

Duncan Grouping	Mean	N	S
A	1.370	16	S20
B	1.335	16	S00

The GLM Procedure

Duncan's Multiple Range Test for TDMw

NOTE: This test controls the Type I comparisonwise error rate, not the experimentwise error rate.

Alpha	0.05
Error Degrees of Freedom	28
Error Mean Square	43.6513

Number of Means	2
Critical Range	4.785

Means with the same letter are not significantly different.

Duncan Grouping	Mean	N	S
A	95.034	16	S00
A			
A	94.858	16	S20

The GLM Procedure

Duncan's Multiple Range Test for TCP

NOTE: This test controls the Type I comparisonwise error rate, not the experimentwise error rate.

Alpha	0.05
Error Degrees of Freedom	28
Error Mean Square	0

Number of Means	2
Critical Range	0

Means with the same letter are not significantly different.

Duncan Grouping	Mean	N	S
A	229.9	16	S20
B	223.2	16	S00

The GLM Procedure

Duncan's Multiple Range Test for TCPw

NOTE: This test controls the Type I comparisonwise error rate, not the experimentwise error rate.

Alpha	0.05
Error Degrees of Freedom	28
Error Mean Square	1.227255

Number of Means	2
Critical Range	.8023

Means with the same letter are not significantly different.

Duncan Grouping	Mean	N	S
A	15.9138	16	S20
A			
A	15.8831	16	S00

The GLM Procedure

Duncan's Multiple Range Test for TDN

NOTE: This test controls the Type I comparisonwise error rate, not the experimentwise error rate.

Alpha	0.05
Error Degrees of Freedom	28
Error Mean Square	0.000178

Number of Means	2
Critical Range	.009666

Means with the same letter are not significantly different.

Duncan Grouping	Mean	N	S
A	1.003750	16	S20
B	0.956875	16	S00

The GLM Procedure

Duncan's Multiple Range Test for TDNw

NOTE: This test controls the Type I comparisonwise error rate, not the experimentwise error rate.

Alpha	0.05
Error Degrees of Freedom	28
Error Mean Square	22.94927

Number of Means	2
Critical Range	3.469

Means with the same letter are not significantly different.

Duncan Grouping	Mean	N	S
A	69.474	16	S20
A			
A	68.189	16	S00

The GLM Procedure

Duncan's Multiple Range Test for DCP

NOTE: This test controls the Type I comparisonwise error rate, not the experimentwise error rate.

Alpha	0.05
Error Degrees of Freedom	28
Error Mean Square	2.343839

Number of Means	2
Critical Range	1.109

Means with the same letter are not significantly different.

Duncan Grouping	Mean	N	S
A	196.5069	16	S20
B	183.4050	16	S00

The GLM Procedure

Duncan's Multiple Range Test for DCPw

NOTE: This test controls the Type I comparisonwise error rate, not the experimentwise error rate.

Alpha	0.05
Error Degrees of Freedom	28
Error Mean Square	0.889005

Number of Means	2
Critical Range	.6828

Means with the same letter are not significantly different.

Duncan Grouping	Mean	N	S
A	13.5938	16	S20
A			
A	13.0450	16	S00

The GLM Procedure

Duncan's Multiple Range Test for Initial

NOTE: This test controls the Type I comparisonwise error rate, not the experimentwise error rate.

Alpha	0.05
Error Degrees of Freedom	28
Error Mean Square	11.55357

Number of Means	2
Critical Range	2.462

Means with the same letter are not significantly different.

Duncan Grouping	Mean	N	S
A	21.625	16	S00
A			
A	21.625	16	S20

The GLM Procedure

Duncan's Multiple Range Test for Final

NOTE: This test controls the Type I comparisonwise error rate, not the experimentwise error rate.

Alpha	0.05
Error Degrees of Freedom	28
Error Mean Square	13.06246

Number of Means	2
Critical Range	2.617

Means with the same letter are not significantly different.

Duncan Grouping	Mean	N	S
A	49.163	16	S20
B	46.656	16	S00

The GLM Procedure

Duncan's Multiple Range Test for TGA

NOTE: This test controls the Type I comparisonwise error rate, not the experimentwise error rate.

Alpha	0.05
Error Degrees of Freedom	28
Error Mean Square	4.961562

Number of Means	2
Critical Range	1.613

Means with the same letter are not significantly different.

Duncan Grouping	Mean	N	S
A	27.5375	16	S20
B	25.0313	16	S00

The GLM Procedure

Duncan's Multiple Range Test for ADG

NOTE: This test controls the Type I comparisonwise error rate, not the experimentwise error rate.

Alpha	0.05
Error Degrees of Freedom	28
Error Mean Square	0.000333

Number of Means	2
Critical Range	.01323

Means with the same letter are not significantly different.

Duncan Grouping	Mean	N	S
A	0.231250	16	S20
B	0.209375	16	S00

The GLM Procedure

Duncan's Multiple Range Test for ADG

NOTE: This test controls the Type I comparisonwise error rate, not the experimentwise error rate.

Alpha	0.05
Error Degrees of Freedom	28
Error Mean Square	0.000333

Number of Means	2
Critical Range	.01323

Means with the same letter are not significantly different.

Duncan Grouping	Mean	N	S
A	0.231250	16	S20
B	0.209375	16	S00

The GLM Procedure

Duncan's Multiple Range Test for TDM

NOTE: This test controls the Type I comparisonwise error rate, not the experimentwise error rate.

Alpha	0.05
Error Degrees of Freedom	28
Error Mean Square	0

Number of Means	2
Critical Range	0

Means with the same letter are not significantly different.

Duncan Grouping	Mean	N	S
A	1.370	16	S20
B	1.335	16	S00

The GLM Procedure

Duncan's Multiple Range Test for DMC

NOTE: This test controls the Type I comparisonwise error rate, not the experimentwise error rate.

Alpha	0.05
Error Degrees of Freedom	28
Error Mean Square	0.304174

Number of Means	2
Critical Range	.3994

Means with the same letter are not significantly different.

Duncan Grouping	Mean	N	S
A	6.4600	16	S00
B	6.0281	16	S20

The GLM Procedure

Duncan's Multiple Range Test for TDN

NOTE: This test controls the Type I comparisonwise error rate, not the experimentwise error rate.

Alpha	0.05
Error Degrees of Freedom	28
Error Mean Square	0.000178

Number of Means	2
Critical Range	.009666

Means with the same letter are not significantly different.

Duncan Grouping	Mean	N	S
A	1.003750	16	S20
B	0.956875	16	S00

The GLM Procedure

Duncan's Multiple Range Test for TDNC

NOTE: This test controls the Type I comparisonwise error rate, not the experimentwise error rate.

Alpha	0.05
Error Degrees of Freedom	28
Error Mean Square	0.150808

Number of Means	2
Critical Range	.2812

Means with the same letter are not significantly different.

Duncan Grouping	Mean	N	S
A	4.6294	16	S00
A			
A	4.4106	16	S20

The GLM Procedure

Duncan's Multiple Range Test for DCPC

NOTE: This test controls the Type I comparisonwise error rate, not the experimentwise error rate.

Alpha	0.05
Error Degrees of Freedom	28
Error Mean Square	5663.106

Number of Means	2
Critical Range	54.50

Means with the same letter are not significantly different.

Duncan Grouping	Mean	N	S
A	884.68	16	S00
A			
A	862.45	16	S20

The GLM Procedure

Duncan's Multiple Range Test for CPC

NOTE: This test controls the Type I comparisonwise error rate, not the experimentwise error rate.

Alpha	0.05
Error Degrees of Freedom	28
Error Mean Square	8498.244

Number of Means	2
Critical Range	66.76

Means with the same letter are not significantly different.

Duncan Grouping	Mean	N	S
A	1079.56	16	S00
B	1011.27	16	S20

The GLM Procedure

Least Squares Means

P	AW LSMEAN	Standard Error	Pr > |t|
P00	35.6875000	0.8317009	<.0001
P25	33.8468750	0.8317009	<.0001

P	BW LSMEAN	Standard Error	Pr > |t|
P00	14.5875000	0.2549170	<.0001
P25	14.0256250	0.2549170	<.0001

P	FoDM LSMEAN	Standard Error	Pr > |t|
P00	557.545000	0.000000	.
P25	522.015000	0.000000	.

Standard

P	Fow LSMEAN	Error	Pr > |t|
P00	38.4506250	0.6591627	<.0001
P25	37.3200000	0.6591627	<.0001

P	FoCP LSMEAN	Standard Error	Pr > |t|
P00	94.3350000	0.0000000	.
P25	76.5700000	0.0000000	.

P	FoCPw LSMEAN	Standard Error	Pr > |t|
P00	6.50500000	0.10712182	<.0001
P25	5.47437500	0.10712182	<.0001

P	TconDM LSMEAN	Standard Error	Pr > |t|
P00	840.425000	0.000000	.
P25	785.610000	0.000000	.

The GLM Procedure

Least Squares Means

P	Tconw LSMEAN	Standard Error	Pr > |t|
P00	57.9593750	0.9929540	<.0001
P25	56.1631250	0.9929540	<.0001

P	TconCP LSMEAN	Standard Error	Pr > |t|
P00	140.390000	0.000000	.
P25	141.810000	0.000000	.

P	TconCPw LSMEAN	Standard Error	Pr > |t|
P00	9.6818750	0.1697659	<.0001
P25	10.1375000	0.1697659	<.0001

Standard

P	TDM LSMEAN	Error	Pr > |t|
P00	1.40000000	0.00000000	.
P25	1.30500000	0.00000000	.

P	TDMw LSMEAN	Standard Error	Pr > |t|
P00	96.4087500	1.6517282	<.0001
P25	93.4831250	1.6517282	<.0001

P	TCP LSMEAN	Standard Error	Pr > |t|
P00	234.725000	0.000000	.
P25	218.380000	0.000000	.

P	TCPw LSMEAN	Standard Error	Pr > |t|
P00	16.1856250	0.2769538	<.0001
P25	15.6112500	0.2769538	<.0001

The GLM Procedure

Least Squares Means

P	TDN LSMEAN	Standard Error	Pr > |t|
P00	1.03062500	0.00333659	<.0001
P25	0.93000000	0.00333659	<.0001

P	TDNw LSMEAN	Standard Error	Pr > |t|
P00	71.1206250	1.1976349	<.0001
P25	66.5425000	1.1976349	<.0001

P	DCP LSMEAN	Standard Error	Pr > |t|
P00	202.953750	0.382740	<.0001
P25	176.958125	0.382740	<.0001

Standard

P	DCPw LSMEAN	Error	Pr > |t|
P00	13.9912500	0.2357177	<.0001
P25	12.6475000	0.2357177	<.0001

	Initial	Standard	
P	LSMEAN	Error	Pr > |t|
P00	21.6250000	0.8497636	<.0001
P25	21.6250000	0.8497636	<.0001

		Standard	
P	Final LSMEAN	Error	Pr > |t|
P00	49.7500000	0.9035505	<.0001
P25	46.0687500	0.9035505	<.0001

		Standard	
P	TGA LSMEAN	Error	Pr > |t|
P00	28.1250000	0.5568641	<.0001
P25	24.4437500	0.5568641	<.0001

The GLM Procedure

Least Squares Means

P	ADG LSMEAN	Standard Error	Pr > |t|
P00	0.23562500	0.00456537	<.0001
P25	0.20500000	0.00456537	<.0001

P	ADG LSMEAN	Standard Error	Pr > |t|
P00	0.23562500	0.00456537	<.0001
P25	0.20500000	0.00456537	<.0001

P	TDM LSMEAN	Standard Error	Pr > |t|
P00	1.40000000	0.00000000	.
P25	1.30500000	0.00000000	.

Standard

P	DMC LSMEAN	Error	Pr > |t|
P00	5.99750000	0.13787985	<.0001
P25	6.49062500	0.13787985	<.0001

P	TDN LSMEAN	Standard Error	Pr > |t|
P00	1.03062500	0.00333659	<.0001
P25	0.93000000	0.00333659	<.0001

P	TDNC LSMEAN	Standard Error	Pr > |t|
P00	4.42375000	0.09708503	<.0001
P25	4.61625000	0.09708503	<.0001

P	DCPC LSMEAN	Standard Error	Pr > |t|
P00	870.161875	18.813403	<.0001
P25	876.965625	18.813403	<.0001

The GLM Procedure

Least Squares Means

P	CPC LSMEAN	Standard Error	Pr > |t|
P00	1007.17375	23.04648	<.0001
P25	1083.65500	23.04648	<.0001

S	AW LSMEAN	Standard Error	Pr > |t|
S00	34.1406250	0.8317009	<.0001
S20	35.3937500	0.8317009	<.0001

S	BW LSMEAN	Standard Error	Pr > |t|
S00	14.1131250	0.2549170	<.0001
S20	14.5000000	0.2549170	<.0001

Standard

S	FoDM LSMEAN	Error	Pr > |t|
S00	533.380000	0.000000	.
S20	546.180000	0.000000	.

S	Fow LSMEAN	Standard Error	Pr > |t|
S00	37.9593750	0.6591627	<.0001
S20	37.8112500	0.6591627	<.0001

S	FoCP LSMEAN	Standard Error	Pr > |t|
S00	84.4400000	0.0000000	.
S20	86.4650000	0.0000000	.

S	FoCPw LSMEAN	Standard Error	Pr > |t|
S00	6.00250000	0.10712182	<.0001
S20	5.97687500	0.10712182	<.0001

The GLM Procedure

Least Squares Means

S	TconDM LSMEAN	Standard Error	Pr > |t|
S00	801.985000	0.000000	.
S20	824.050000	0.000000	.

S	Tconw LSMEAN	Standard Error	Pr > |t|
S00	57.0737500	0.9929540	<.0001
S20	57.0487500	0.9929540	<.0001

S	TconCP LSMEAN	Standard Error	Pr > |t|
S00	138.770000	0.000000	.
S20	143.430000	0.000000	.

TconCPw Standard

S	LSMEAN	Error	Pr > |t|
S00	9.88187500	0.16976587	<.0001
S20	9.93750000	0.16976587	<.0001

S	TDM LSMEAN	Standard Error	Pr > |t|
S00	1.33500000	0.00000000	.
S20	1.37000000	0.00000000	.

S	TDMw LSMEAN	Standard Error	Pr > |t|
S00	95.0343750	1.6517282	<.0001
S20	94.8575000	1.6517282	<.0001

S	TCP LSMEAN	Standard Error	Pr > |t|
S00	223.210000	0.000000	.
S20	229.895000	0.000000	.

The GLM Procedure

Least Squares Means

S	TCPw LSMEAN	Standard Error	Pr > |t|
S00	15.8831250	0.2769538	<.0001
S20	15.9137500	0.2769538	<.0001

S	TDN LSMEAN	Standard Error	Pr > |t|
S00	0.95687500	0.00333659	<.0001
S20	1.00375000	0.00333659	<.0001

S	TDNw LSMEAN	Standard Error	Pr > |t|
S00	68.1887500	1.1976349	<.0001
S20	69.4743750	1.1976349	<.0001

Standard

S	DCP LSMEAN	Error	Pr > |t|
S00	183.405000	0.382740	<.0001
S20	196.506875	0.382740	<.0001

S	DCPw LSMEAN	Standard Error	Pr > |t|
S00	13.0450000	0.2357177	<.0001
S20	13.5937500	0.2357177	<.0001

S	Initial LSMEAN	Standard Error	Pr > |t|
S00	21.6250000	0.8497636	<.0001
S20	21.6250000	0.8497636	<.0001

S	Final LSMEAN	Standard Error	Pr > |t|
S00	46.6562500	0.9035505	<.0001
S20	49.1625000	0.9035505	<.0001

The GLM Procedure

Least Squares Means

S	TGA LSMEAN	Standard Error	Pr > |t|
S00	25.0312500	0.5568641	<.0001
S20	27.5375000	0.5568641	<.0001

S	ADG LSMEAN	Standard Error	Pr > |t|
S00	0.20937500	0.00456537	<.0001
S20	0.23125000	0.00456537	<.0001

S	ADG LSMEAN	Standard Error	Pr > |t|
S00	0.20937500	0.00456537	<.0001
S20	0.23125000	0.00456537	<.0001

Standard

S	TDM LSMEAN	Error	Pr > |t|
S00	1.33500000	0.00000000	.
S20	1.37000000	0.00000000	.

		Standard	
S	DMC LSMEAN	Error	Pr > |t|
S00	6.46000000	0.13787985	<.0001
S20	6.02812500	0.13787985	<.0001

		Standard	
S	TDN LSMEAN	Error	Pr > |t|
S00	0.95687500	0.00333659	<.0001
S20	1.00375000	0.00333659	<.0001

		Standard	
S	TDNC LSMEAN	Error	Pr > |t|
S00	4.62937500	0.09708503	<.0001
S20	4.41062500	0.09708503	<.0001

The GLM Procedure

Least Squares Means

S	DCPC LSMEAN	Standard Error	Pr > |t|
S00	884.676875	18.813403	<.0001
S20	862.450625	18.813403	<.0001

S	CPC LSMEAN	Standard Error	Pr > |t|
S00	1079.55938	23.04648	<.0001
S20	1011.26938	23.04648	<.0001

P	S	AW LSMEAN	Standard Error	Pr > |t|
P00	S00	35.0000000	1.1762027	<.0001
P00	S20	36.3750000	1.1762027	<.0001
P25	S00	33.2812500	1.1762027	<.0001
P25	S20	34.4125000	1.1762027	<.0001

P	S	BW LSMEAN	Standard Error	Pr > |t|
P00	S00	14.3725000	0.3605071	<.0001
P00	S20	14.8025000	0.3605071	<.0001
P25	S00	13.8537500	0.3605071	<.0001
P25	S20	14.1975000	0.3605071	<.0001

P	S	FoDM LSMEAN	Standard Error	Pr > |t|
P00	S00	550.880000	0.000000	.
P00	S20	564.210000	0.000000	.
P25	S00	515.880000	0.000000	.
P25	S20	528.150000	0.000000	.

P	S	Fow LSMEAN	Standard Error	Pr > |t|
P00	S00	38.6112500	0.9321969	<.0001
P00	S20	38.2900000	0.9321969	<.0001
P25	S00	37.3075000	0.9321969	<.0001
P25	S20	37.3325000	0.9321969	<.0001

The GLM Procedure

Least Squares Means

P	S	FoCP LSMEAN	Standard Error	Pr > |t|
P00	S00	93.2100000	0.0000000	.
P00	S20	95.4600000	0.0000000	.
P25	S00	75.6700000	0.0000000	.
P25	S20	77.4700000	0.0000000	.

P	S	FoCPw LSMEAN	Standard Error	Pr > |t|
P00	S00	6.53250000	0.15149313	<.0001
P00	S20	6.47750000	0.15149313	<.0001
P25	S00	5.47250000	0.15149313	<.0001
P25	S20	5.47625000	0.15149313	<.0001

P	S	TconDM LSMEAN	Standard Error	Pr > |t|
P00	S00	828.940000	0.000000	.

P00	S20	851.910000	0.000000	.
P25	S00	775.030000	0.000000	.
P25	S20	796.190000	0.000000	.

P	S	Tconw LSMEAN	Standard Error	Pr > |t|
P00	S00	58.1012500	1.4042490	<.0001
P00	S20	57.8175000	1.4042490	<.0001
P25	S00	56.0462500	1.4042490	<.0001
P25	S20	56.2800000	1.4042490	<.0001

P	S	TconCP LSMEAN	Standard Error	Pr > |t|
P00	S00	138.030000	0.000000	.
P00	S20	142.750000	0.000000	.
P25	S00	139.510000	0.000000	.
P25	S20	144.110000	0.000000	.

P	S	TconCPw LSMEAN	Standard Error	Pr > |t|
P00	S00	9.6750000	0.2400852	<.0001

P00	S20	9.6887500	0.2400852	<.0001
P25	S00	10.0887500	0.2400852	<.0001

The GLM Procedure

Least Squares Means

P	S	TconCPw LSMEAN	Standard Error	Pr > |t|
P25	S20	10.1862500	0.2400852	<.0001

P	S	TDM LSMEAN	Standard Error	Pr > |t|
P00	S00	1.38000000	0.00000000	.
P00	S20	1.42000000	0.00000000	.
P25	S00	1.29000000	0.00000000	.
P25	S20	1.32000000	0.00000000	.

P	S	TDMw LSMEAN	Standard Error	Pr > |t|
P00	S00	96.7125000	2.3358964	<.0001
P00	S20	96.1050000	2.3358964	<.0001
P25	S00	93.3562500	2.3358964	<.0001
P25	S20	93.6100000	2.3358964	<.0001

P	S	TCP LSMEAN	Standard Error	Pr > |t|
P00	S00	231.240000	0.000000	.
P00	S20	238.210000	0.000000	.
P25	S00	215.180000	0.000000	.
P25	S20	221.580000	0.000000	.

P	S	TCPw LSMEAN	Standard Error	Pr > |t|
P00	S00	16.2062500	0.3916719	<.0001
P00	S20	16.1650000	0.3916719	<.0001
P25	S00	15.5600000	0.3916719	<.0001
P25	S20	15.6625000	0.3916719	<.0001

P	S	TDN LSMEAN	Standard Error	Pr > |t|
P00	S00	1.01125000	0.00471865	<.0001
P00	S20	1.05000000	0.00471865	<.0001
P25	S00	0.90250000	0.00471865	<.0001
P25	S20	0.95750000	0.00471865	<.0001

The GLM Procedure

Least Squares Means

P	S	TDNw LSMEAN	Standard Error	Pr > |t|
P00	S00	70.9800000	1.6937116	<.0001
P00	S20	71.2612500	1.6937116	<.0001
P25	S00	65.3975000	1.6937116	<.0001
P25	S20	67.6875000	1.6937116	<.0001

P	S	DCP LSMEAN	Standard Error	Pr > |t|
P00	S00	196.777500	0.541276	<.0001
P00	S20	209.130000	0.541276	<.0001
P25	S00	170.032500	0.541276	<.0001
P25	S20	183.883750	0.541276	<.0001

P	S	DCPw LSMEAN	Standard Error	Pr > |t|
P00	S00	13.7937500	0.3333552	<.0001

P00	S20	14.1887500	0.3333552	<.0001
P25	S00	12.2962500	0.3333552	<.0001
P25	S20	12.9987500	0.3333552	<.0001

P	S	Initial LSMEAN	Standard Error	Pr > |t|
P00	S00	21.6250000	1.2017472	<.0001
P00	S20	21.6250000	1.2017472	<.0001
P25	S00	21.6250000	1.2017472	<.0001
P25	S20	21.6250000	1.2017472	<.0001

P	S	Final LSMEAN	Standard Error	Pr > |t|
P00	S00	48.3750000	1.2778133	<.0001
P00	S20	51.1250000	1.2778133	<.0001
P25	S00	44.9375000	1.2778133	<.0001
P25	S20	47.2000000	1.2778133	<.0001

P	S	TGA LSMEAN	Standard Error	Pr > |t|
P00	S00	26.7500000	0.7875248	<.0001

P00	S20	29.5000000	0.7875248	<.0001
P25	S00	23.3125000	0.7875248	<.0001

The GLM Procedure

Least Squares Means

P	S	TGA LSMEAN	Standard Error	Pr > |t|
P25	S20	25.5750000	0.7875248	<.0001

P	S	ADG LSMEAN	Standard Error	Pr > |t|
P00	S00	0.22375000	0.00645641	<.0001
P00	S20	0.24750000	0.00645641	<.0001
P25	S00	0.19500000	0.00645641	<.0001
P25	S20	0.21500000	0.00645641	<.0001

P	S	ADG LSMEAN	Standard Error	Pr > |t|
P00	S00	0.22375000	0.00645641	<.0001
P00	S20	0.24750000	0.00645641	<.0001
P25	S00	0.19500000	0.00645641	<.0001
P25	S20	0.21500000	0.00645641	<.0001

P	S	TDM LSMEAN	Standard Error	Pr > |t|
P00	S00	1.38000000	0.00000000	.
P00	S20	1.42000000	0.00000000	.
P25	S00	1.29000000	0.00000000	.
P25	S20	1.32000000	0.00000000	.

P	S	DMC LSMEAN	Standard Error	Pr > |t|
P00	S00	6.21375000	0.19499156	<.0001
P00	S20	5.78125000	0.19499156	<.0001
P25	S00	6.70625000	0.19499156	<.0001
P25	S20	6.27500000	0.19499156	<.0001

P	S	TDN LSMEAN	Standard Error	Pr > |t|
P00	S00	1.01125000	0.00471865	<.0001
P00	S20	1.05000000	0.00471865	<.0001
P25	S00	0.90250000	0.00471865	<.0001
P25	S20	0.95750000	0.00471865	<.0001

The GLM Procedure

Least Squares Means

P	S	TDNC LSMEAN	Standard Error	Pr > |t|
P00	S00	4.56125000	0.13729896	<.0001
P00	S20	4.28625000	0.13729896	<.0001
P25	S00	4.69750000	0.13729896	<.0001
P25	S20	4.53500000	0.13729896	<.0001

P	S	DCPC LSMEAN	Standard Error	Pr > |t|
P00	S00	886.350000	26.606170	<.0001
P00	S20	853.973750	26.606170	<.0001
P25	S00	883.003750	26.606170	<.0001
P25	S20	870.927500	26.606170	<.0001

P	S	CPC LSMEAN	Standard Error	Pr > |t|
P00	S00	1041.60125	32.59264	<.0001

P00	S20	972.74625	32.59264	<.0001
P25	S00	1117.51750	32.59264	<.0001
P25	S20	1049.79250	32.59264	<.0001

The MEANS Procedure

Variable	Std Dev
AW	3.3585617
BW	1.0294083
FoDM	19.1867010
Fow	2.5734112
FoCP	9.0837888
FoCPw	0.6635801
TconDM	30.0209281
Tconw	3.8857131
TconCP	2.4749376
TconCPw	0.6865316
TDM	0.0514938
TDMw	6.4569152
TCP	8.9720638
TCPw	1.0932524
TDN	0.0579479
TDNw	5.1791960
DCP	14.8645206
DCPw	1.1630825
Initial	3.2304000
Final	4.1148323
TGA	3.1007917

ADG	0.0258388
DMC	0.6209870
TDNC	0.3986752
DCPC	72.6710518
CPC	101.9260678

ƒƒƒƒƒƒƒƒƒƒƒƒƒƒƒƒƒƒƒƒƒƒƒƒƒƒ
