# Supplementary material for: Influence of substituting 25% alfalfa hay with Panicum maximum cv. Mombasa with or without spirulina supplementation on the productive performance of fattening Barki lambs
Source: Sci Rep. 2026 Jan 10;16:1347. doi: 10.1038/s41598-025-28525-1 (PMC12796356; doi:10.1038/s41598-025-28525-1)
Supplement: Supplementary file 1 — Supplementary Material 1 [file 41598_2025_28525_MOESM1_ESM.zip › Meteab_Supplementary/Raw Data/fermantation fattening factoral.pdf]

The MEANS Procedure

ffffffffffffffffffffffffffffffffffffffffffffffffffffffffffffffffffffffffffffffffff
ff

Variable	N	Mean	Std Dev	Minimum	Maximum
TIME	72	3.0000000	2.4666794	0	6.0000000
R	72	6.5000000	3.4762778	1.0000000	12.0000000
VFA	72	7.3591667	1.4641373	5.2000000	10.4000000
NH3	72	17.4447222	2.0330640	13.9300000	22.7800000
PH	72	6.4566667	0.2301622	5.9800000	6.8800000

ffffffffffffffffffffffffffffffffffffffffffffffffffffffffffffffffffffffffffffffffff
ff

The SAS System 17:53 Friday, October 4, 2002 97

The GLM Procedure

Class Level Information

Class	Levels	Values
T	2	P00 P25
ALGAE	2	S00 S20
TIME	3	0 3 6

Number of observations 72

The SAS System 17:53 Friday, October 4, 2002 98

The GLM Procedure

Dependent Variable: VFA

Source	DF	Sum of Squares	Mean Square	F Value	Pr > F
Model	11	141.4045500	12.8549591	71.43	<.0001
Error	60	10.7980000	0.1799667		
Corrected Total	71	152.2025500			

R-Square	Coeff Var	Root MSE	VFA Mean
0.929055	5.764576	0.424225	7.359167

Source	DF	Type III SS	Mean Square	F Value	Pr > F
T	1	46.17605000	46.17605000	256.58	<.0001
ALGAE	1	18.95227222	18.95227222	105.31	<.0001
TIME	2	71.51410000	35.75705000	198.69	<.0001
T*ALGAE	1	0.78960556	0.78960556	4.39	0.0404
T*TIME	2	1.46943333	0.73471667	4.08	0.0218
ALGAE*TIME	2	1.22754444	0.61377222	3.41	0.0396
T*ALGAE*TIME	2	1.27554444	0.63777222	3.54	0.0351

The GLM Procedure

Dependent Variable: NH3

Source	DF	Sum of Squares	Mean Square	F Value	Pr > F
Model	11	274.0180278	24.9107298	76.85	<.0001
Error	60	19.4497667	0.3241628		
Corrected Total	71	293.4677944			

R-Square	Coeff Var	Root MSE	NH3 Mean
0.933724	3.263755	0.569353	17.44472

Source	DF	Type III SS	Mean Square	F Value	Pr > F
T	1	98.7012500	98.7012500	304.48	<.0001
ALGAE	1	31.2313389	31.2313389	96.34	<.0001
TIME	2	139.6398861	69.8199431	215.39	<.0001
T*ALGAE	1	3.1166722	3.1166722	9.61	0.0029
T*TIME	2	0.4077083	0.2038542	0.63	0.5367
ALGAE*TIME	2	0.5027528	0.2513764	0.78	0.4650
T*ALGAE*TIME	2	0.4184194	0.2092097	0.65	0.5281

The GLM Procedure

Dependent Variable: PH

Source	DF	Sum of Squares	Mean Square	F Value	Pr > F
Model	11	3.40686667	0.30971515	52.44	<.0001
Error	60	0.35433333	0.00590556		
Corrected Total	71	3.76120000			

R-Square	Coeff Var	Root MSE	PH Mean
0.905792	1.190206	0.076848	6.456667

Source	DF	Type III SS	Mean Square	F Value	Pr > F
T	1	0.22445000	0.22445000	38.01	<.0001
ALGAE	1	0.08133889	0.08133889	13.77	0.0005
TIME	2	3.05875833	1.52937917	258.97	<.0001
T*ALGAE	1	0.01175556	0.01175556	1.99	0.1634
T*TIME	2	0.01547500	0.00773750	1.31	0.2774
ALGAE*TIME	2	0.00168611	0.00084306	0.14	0.8673
T*ALGAE*TIME	2	0.01340278	0.00670139	1.13	0.3283

The GLM Procedure
Least Squares Means

T	VFA LSMEAN	Standard Error	Pr > |t|
P00	8.16000000	0.07070413	<.0001
P25	6.55833333	0.07070413	<.0001

T	NH3 LSMEAN	Standard Error	Pr > |t|
P00	18.6155556	0.0948922	<.0001
P25	16.2738889	0.0948922	<.0001

T	PH LSMEAN	Standard Error	Pr > |t|
P00	6.40083333	0.01280794	<.0001
P25	6.51250000	0.01280794	<.0001

ALGAE	VFA LSMEAN	Standard Error	Pr > |t|
S00	6.84611111	0.07070413	<.0001
S20	7.87222222	0.07070413	<.0001

ALGAE	NH3 LSMEAN	Standard Error	Pr > |t|
S00	16.7861111	0.0948922	<.0001
S20	18.1033333	0.0948922	<.0001

ALGAE	PH LSMEAN	Standard Error	Pr > |t|
S00	6.49027778	0.01280794	<.0001
S20	6.42305556	0.01280794	<.0001

TIME	VFA LSMEAN	Standard Error	Pr > |t|
0	6.06083333	0.08659452	<.0001
3	8.48333333	0.08659452	<.0001
6	7.53333333	0.08659452	<.0001

The GLM Procedure
Least Squares Means

TIME	NH3 LSMEAN	Standard Error	Pr > |t|
0	15.7375000	0.1162187	<.0001
3	19.1487500	0.1162187	<.0001
6	17.4479167	0.1162187	<.0001

<table>
<tr><th>TIME</th><th>PH LSMEAN</th><th>Standard Error</th><th>Pr > |t|</th></tr>
<tr><td>0</td><td>6.72750000</td><td>0.01568645</td><td><.0001</td></tr>
<tr><td>3</td><td>6.22791667</td><td>0.01568645</td><td><.0001</td></tr>
<tr><td>6</td><td>6.41458333</td><td>0.01568645</td><td><.0001</td></tr>
</table>

<table>
<tr><th>T</th><th>ALGAE</th><th>VFA LSMEAN</th><th>Standard Error</th><th>Pr > |t|</th></tr>
<tr><td>P00</td><td>S00</td><td>7.54222222</td><td>0.09999074</td><td><.0001</td></tr>
<tr><td>P00</td><td>S20</td><td>8.77777778</td><td>0.09999074</td><td><.0001</td></tr>
<tr><td>P25</td><td>S00</td><td>6.15000000</td><td>0.09999074</td><td><.0001</td></tr>
<tr><td>P25</td><td>S20</td><td>6.96666667</td><td>0.09999074</td><td><.0001</td></tr>
</table>

<table>
<tr><th>T</th><th>ALGAE</th><th>NH3 LSMEAN</th><th>Standard Error</th><th>Pr > |t|</th></tr>
<tr><td>P00</td><td>S00</td><td>17.7488889</td><td>0.1341978</td><td><.0001</td></tr>
<tr><td>P00</td><td>S20</td><td>19.4822222</td><td>0.1341978</td><td><.0001</td></tr>
<tr><td>P25</td><td>S00</td><td>15.8233333</td><td>0.1341978</td><td><.0001</td></tr>
<tr><td>P25</td><td>S20</td><td>16.7244444</td><td>0.1341978</td><td><.0001</td></tr>
</table>

<table>
<tr><th>T</th><th>ALGAE</th><th>PH LSMEAN</th><th>Standard Error</th><th>Pr > |t|</th></tr>
<tr><td>P00</td><td>S00</td><td>6.42166667</td><td>0.01811316</td><td><.0001</td></tr>
<tr><td>P00</td><td>S20</td><td>6.38000000</td><td>0.01811316</td><td><.0001</td></tr>
<tr><td>P25</td><td>S00</td><td>6.55888889</td><td>0.01811316</td><td><.0001</td></tr>
<tr><td>P25</td><td>S20</td><td>6.46611111</td><td>0.01811316</td><td><.0001</td></tr>
</table>

<table>
<tr><th>T</th><th>TIME</th><th>VFA LSMEAN</th><th>Standard Error</th><th>Pr > |t|</th></tr>
<tr><td>P00</td><td>0</td><td>6.66333333</td><td>0.12246315</td><td><.0001</td></tr>
<tr><td>P00</td><td>3</td><td>9.41666667</td><td>0.12246315</td><td><.0001</td></tr>
</table>

T	TIME	VFA LSMEAN	Standard Error	Pr > |t|
P00	6	8.40000000	0.12246315	<.0001
P25	0	5.45833333	0.12246315	<.0001
P25	3	7.55000000	0.12246315	<.0001
P25	6	6.66666667	0.12246315	<.0001

T	TIME	NH3 LSMEAN	Standard Error	Pr > |t|
P00	0	16.9333333	0.1643580	<.0001
P00	3	20.3966667	0.1643580	<.0001
P00	6	18.5166667	0.1643580	<.0001
P25	0	14.5416667	0.1643580	<.0001
P25	3	17.9008333	0.1643580	<.0001
P25	6	16.3791667	0.1643580	<.0001

T	TIME	PH LSMEAN	Standard Error	Pr > |t|
P00	0	6.68500000	0.02218399	<.0001
P00	3	6.17916667	0.02218399	<.0001
P00	6	6.33833333	0.02218399	<.0001
P25	0	6.77000000	0.02218399	<.0001
P25	3	6.27666667	0.02218399	<.0001
P25	6	6.49083333	0.02218399	<.0001

ALGAE	TIME	VFA LSMEAN	Standard Error	Pr > |t|
S00	0	5.71333333	0.12246315	<.0001
S00	3	7.81666667	0.12246315	<.0001
S00	6	7.00833333	0.12246315	<.0001
S20	0	6.40833333	0.12246315	<.0001
S20	3	9.15000000	0.12246315	<.0001
S20	6	8.05833333	0.12246315	<.0001

ALGAE	TIME	NH3 LSMEAN	Standard Error	Pr > |t|

S00	0	15.0616667	0.1643580	<.0001
S00	3	18.3975000	0.1643580	<.0001
S00	6	16.8991667	0.1643580	<.0001

The GLM Procedure
Least Squares Means

ALGAE	TIME	NH3 LSMEAN	Standard Error	Pr > |t|
S20	0	16.4133333	0.1643580	<.0001
S20	3	19.9000000	0.1643580	<.0001
S20	6	17.9966667	0.1643580	<.0001

ALGAE	TIME	PH LSMEAN	Standard Error	Pr > |t|
S00	0	6.75833333	0.02218399	<.0001
S00	3	6.25750000	0.02218399	<.0001
S00	6	6.45500000	0.02218399	<.0001
S20	0	6.69666667	0.02218399	<.0001
S20	3	6.19833333	0.02218399	<.0001
S20	6	6.37416667	0.02218399	<.0001

T	ALGAE	TIME	VFA LSMEAN	Standard Error	Pr > |t|
P00	S00	0	6.1100000	0.1731890	<.0001
P00	S00	3	8.8333333	0.1731890	<.0001
P00	S00	6	7.6833333	0.1731890	<.0001
P00	S20	0	7.2166667	0.1731890	<.0001
P00	S20	3	10.0000000	0.1731890	<.0001
P00	S20	6	9.1166667	0.1731890	<.0001
P25	S00	0	5.3166667	0.1731890	<.0001
P25	S00	3	6.8000000	0.1731890	<.0001
P25	S00	6	6.3333333	0.1731890	<.0001
P25	S20	0	5.6000000	0.1731890	<.0001
P25	S20	3	8.3000000	0.1731890	<.0001
P25	S20	6	7.0000000	0.1731890	<.0001

T	ALGAE	TIME	NH3 LSMEAN	Standard Error	Pr > |t|

P00	S00	0	15.9833333	0.2324374	<.0001
P00	S00	3	19.3966667	0.2324374	<.0001
P00	S00	6	17.8666667	0.2324374	<.0001
P00	S20	0	17.8833333	0.2324374	<.0001
P00	S20	3	21.3966667	0.2324374	<.0001
P00	S20	6	19.1666667	0.2324374	<.0001
P25	S00	0	14.1400000	0.2324374	<.0001
P25	S00	3	17.3983333	0.2324374	<.0001
P25	S00	6	15.9316667	0.2324374	<.0001

The GLM Procedure
Least Squares Means

T	ALGAE	TIME	NH3 LSMEAN	Standard Error	Pr > |t|
P25	S20	0	14.9433333	0.2324374	<.0001
P25	S20	3	18.4033333	0.2324374	<.0001
P25	S20	6	16.8266667	0.2324374	<.0001

T	ALGAE	TIME	PH LSMEAN	Standard Error	Pr > |t|
P00	S00	0	6.70166667	0.03137291	<.0001
P00	S00	3	6.21333333	0.03137291	<.0001
P00	S00	6	6.35000000	0.03137291	<.0001
P00	S20	0	6.66833333	0.03137291	<.0001
P00	S20	3	6.14500000	0.03137291	<.0001
P00	S20	6	6.32666667	0.03137291	<.0001
P25	S00	0	6.81500000	0.03137291	<.0001
P25	S00	3	6.30166667	0.03137291	<.0001
P25	S00	6	6.56000000	0.03137291	<.0001
P25	S20	0	6.72500000	0.03137291	<.0001
P25	S20	3	6.25166667	0.03137291	<.0001
P25	S20	6	6.42166667	0.03137291	<.0001

The GLM Procedure

Duncan's Multiple Range Test for VFA

NOTE: This test controls the Type I comparisonwise error rate, not the experimentwise error

rate.

Alpha	0.05
Error Degrees of Freedom	60
Error Mean Square	0.179967

Number of Means	2
Critical Range	.2000

Means with the same letter are not significantly different.

Duncan Grouping	Mean	N	T
A	8.16[a]	36	P00
B	6.55[b]	36	P25

The GLM Procedure

Duncan's Multiple Range Test for NH3

NOTE: This test controls the Type I comparisonwise error rate, not the experimentwise error
rate.

Alpha	0.05
Error Degrees of Freedom	60
Error Mean Square	0.324163

Number of Means	2
Critical Range	.2684

Means with the same letter are not significantly different.

Duncan Grouping	Mean	N	T
A	18.61[a]	36	P00

B 16.27[b] 36 P25

The GLM Procedure

Duncan's Multiple Range Test for PH

NOTE: This test controls the Type I comparisonwise error rate, not the experimentwise error rate.

Alpha	0.05
Error Degrees of Freedom	60
Error Mean Square	0.005906

Number of Means	2
Critical Range	.03623

Means with the same letter are not significantly different.

Duncan Grouping	Mean	N	T
A	6.51[a]	36	P25
B	6.40[b]	36	P00

The GLM Procedure

Duncan's Multiple Range Test for VFA

NOTE: This test controls the Type I comparisonwise error rate, not the experimentwise error rate.

Alpha	0.05
Error Degrees of Freedom	60
Error Mean Square	0.179967

Number of Means	2
Critical Range	.2000

Means with the same letter are not significantly different.

Duncan Grouping	Mean	N	ALGAE
A	7.87^{a}	36	S20
B	6.84^{b}	36	S00

The GLM Procedure

Duncan's Multiple Range Test for NH_3

NOTE: This test controls the Type I comparisonwise error rate, not the experimentwise error
rate.

Alpha	0.05
Error Degrees of Freedom	60
Error Mean Square	0.324163

Number of Means	2
Critical Range	.2684

Means with the same letter are not significantly different.

Duncan Grouping	Mean	N	ALGAE
A	18.10^{a}	36	S20
B	16.78^{b}	36	S00

The GLM Procedure

Duncan's Multiple Range Test for PH

NOTE: This test controls the Type I comparisonwise error rate, not the experimentwise error rate.

**Alpha**	**0.05**
**Error Degrees of Freedom**	**60**
**Error Mean Square**	**0.005906**

**Number of Means**	**2**
**Critical Range**	**.03623**

Means with the same letter are not significantly different.

Duncan Grouping	Mean	N	ALGAE
A	6.49[a]	36	S00
B	6.42[b]	36	S20

The GLM Procedure

Duncan's Multiple Range Test for VFA

NOTE: This test controls the Type I comparisonwise error rate, not the experimentwise error rate.

**Alpha**	**0.05**
**Error Degrees of Freedom**	**60**
**Error Mean Square**	**0.179967**

**Number of Means**	**2**	**3**
**Critical Range**	**.2450**	**.2577**

Means with the same letter are not significantly different.

Duncan Grouping	Mean	N	TIME

A	8.48[a]	24	3
B	7.53[b]	24	6
C	6.06[c]	24	0

The GLM Procedure

Duncan's Multiple Range Test for NH3

NOTE: This test controls the Type I comparisonwise error rate, not the experimentwise error rate.

Alpha	0.05
Error Degrees of Freedom	60
Error Mean Square	0.324163

Number of Means	2	3
Critical Range	.3288	.3459

Means with the same letter are not significantly different.

Duncan Grouping	Mean	N	TIME
A	19.14[a]	24	3
B	17.44[b]	24	6
C	15.73[c]	24	0

The GLM Procedure

Duncan's Multiple Range Test for PH

NOTE: This test controls the Type I comparisonwise error rate, not the experimentwise error rate.

**Alpha**	**0.05**
**Error Degrees of Freedom**	**60**
**Error Mean Square**	**0.005906**

**Number of Means**	**2**	**3**
**Critical Range**	**.04438**	**.04668**

Means with the same letter are not significantly different.

Duncan Grouping	Mean	N	TIME
A	6.72[a]	24	0
B	6.41[b]	24	6
C	6.22[c]	24	3
