# Supplementary material for: Influence of substituting 25% alfalfa hay with Panicum maximum cv. Mombasa with or without spirulina supplementation on the productive performance of fattening Barki lambs
Source: Sci Rep. 2026 Jan 10;16:1347. doi: 10.1038/s41598-025-28525-1 (PMC12796356; doi:10.1038/s41598-025-28525-1)
Supplement: Supplementary file 1 — Supplementary Material 1 [file 41598_2025_28525_MOESM1_ESM.zip › Meteab_Supplementary/Raw Data/fermantation fattening FACTORIAL data.sas.pdf]

```
DATA ONE;
INPUT T$    ALGAE$      TIME  R     VFA   NH3 PH;
CARDS;
P00   S00   0     1     5.60  15.89 6.80
P00   S00   0     1     6.65  16.15 6.73
P00   S00   0     1     5.75  15.32 6.75
P00   S00   0     1     6.66  16.05 6.67
P00   S00   0     1     5.45  16.11 6.66
P00   S00   0     1     6.55  16.38 6.60
P00   S00   3     2     8.40  19.77 6.31
P00   S00   3     2     8.50  18.48 6.12
P00   S00   3     2     8.40  19.24 6.29
P00   S00   3     2     9.30  19.67 6.14
P00   S00   3     2     9.80  19.48 6.15
P00   S00   3     2     8.60  19.74 6.27
P00   S00   6     3     7.90  17.57 6.46
P00   S00   6     3     6.90  17.44 6.35
P00   S00   6     3     7.60  17.79 6.32
P00   S00   6     3     7.80  17.04 6.30
P00   S00   6     3     7.90  18.68 6.31
P00   S00   6     3     8.00  18.68 6.36
P00   S20   0     4     7.80  17.87 6.62
P00   S20   0     4     7.50  18.16 6.75
P00   S20   0     4     6.60  18.50 6.66
P00   S20   0     4     6.30  18.87 6.71
P00   S20   0     4     7.20  17.04 6.62
P00   S20   0     4     7.90  16.86 6.65
P00   S20   3     5     9.40  21.04 6.12
P00   S20   3     5     10.10 20.67 6.23
P00   S20   3     5     10.00 21.03 6.25
P00   S20   3     5     10.20 22.78 6.09
```

P00	S20	3	5	10.40	21.83	6.12
P00	S20	3	5	9.90	21.03	6.06
P00	S20	6	6	8.80	19.04	6.23
P00	S20	6	6	9.40	19.13	6.40
P00	S20	6	6	8.80	19.33	6.40
P00	S20	6	6	9.70	19.04	6.30
P00	S20	6	6	8.70	19.13	6.30
P00	S20	6	6	9.30	19.33	6.33
P25	S00	0	7	5.20	14.47	6.85
P25	S00	0	7	5.40	13.93	6.72
P25	S00	0	7	5.20	14.02	6.75
P25	S00	0	7	5.35	14.47	6.88
P25	S00	0	7	5.45	13.93	6.84
P25	S00	0	7	5.30	14.02	6.85
P25	S00	3	8	7.00	17.50	6.40
P25	S00	3	8	7.00	17.79	6.29
P25	S00	3	8	6.50	17.30	6.28
P25	S00	3	8	6.90	17.57	6.31
P25	S00	3	8	6.50	16.44	6.29
P25	S00	3	8	6.90	17.79	6.24
P25	S00	6	9	6.30	15.60	6.61
P25	S00	6	9	6.70	15.56	6.59
P25	S00	6	9	6.30	16.09	6.47
P25	S00	6	9	6.50	16.28	6.65
P25	S00	6	9	6.30	16.08	6.45
P25	S00	6	9	5.90	15.98	6.59
P25	S20	0	10	5.30	14.32	6.71
P25	S20	0	10	5.60	14.05	6.72
P25	S20	0	10	5.80	15.96	6.80
P25	S20	0	10	5.70	15.32	6.70
P25	S20	0	10	5.90	14.05	6.60

```
P25   S20   0     10    5.30  15.96 6.82
P25   S20   3     11    8.70  18.47 5.98
P25   S20   3     11    8.60  19.03 6.33
P25   S20   3     11    8.50  18.21 6.25
P25   S20   3     11    7.80  17.67 6.38
P25   S20   3     11    8.30  18.03 6.34
P25   S20   3     11    7.90  19.01 6.23
P25   S20   6     12    7.80  16.93 6.46
P25   S20   6     12    6.70  16.44 6.47
P25   S20   6     12    6.40  17.79 6.41
P25   S20   6     12    7.50  16.57 6.39
P25   S20   6     12    6.90  16.44 6.38
P25   S20   6     12    6.70  16.79 6.42
;
PROC MEANS;
PROC GLM;
CLASS T ALGAE TIME;
MODEL VFA NH3 PH =T ALGAE TIME T*ALGAE T*TIME ALGAE*TIME
T*ALGAE*TIME/SS3;
LSMEANS T ALGAE TIME T*ALGAE T*TIME ALGAE*TIME
T*ALGAE*TIME/STDERR;
MEANS T ALGAE TIME/DUNCAN;
RUN;
```
