# Supplementary material for: Influence of substituting 25% alfalfa hay with Panicum maximum cv. Mombasa with or without spirulina supplementation on the productive performance of fattening Barki lambs
Source: Sci Rep. 2026 Jan 10;16:1347. doi: 10.1038/s41598-025-28525-1 (PMC12796356; doi:10.1038/s41598-025-28525-1)
Supplement: Supplementary file 1 — Supplementary Material 1 [file 41598_2025_28525_MOESM1_ESM.zip › Meteab_Supplementary/Raw Data/Fermantation Mixed result.pdf]

The Mixed Procedure

Model Information

Data Set	WORK.RUMEN
Dependent Variable	VFA
Covariance Structure	Compound Symmetry
Subject Effect	ID(A*B)
Estimation Method	REML
Residual Variance Method	Profile
Fixed Effects SE Method	Prasad-Rao-Jeske-Kackar-Harville
Degrees of Freedom Method	Kenward-Roger

Class Level Information

Class	Levels	Values
ID	24	1 2 3 4 5 6 7 8 9 10 11 12 13 14 15 16 17 18 19 20 21 22 23 24
A	2	P00 P25
B	2	S00 S20
time	3	0 3 6

Dimensions

Covariance Parameters	2
Columns in X	36
Columns in Z	0
Subjects	24
Max Obs Per Subject	3
Observations Used	72
Observations Not Used	0
Total Observations	72

Iteration History

Iteration	Evaluations	-2 Res Log Like	Criterion
0	1	88.87471979	
1	1	87.97169871	0.00000000

Convergence criteria met.

The Mixed Procedure

Covariance Parameter Estimates

Cov Parm	Subject	Estimate
CS	ID(A*B)	-0.02103
Residual		0.2010

Fit Statistics

-2 Res Log Likelihood	88.0
AIC (smaller is better)	92.0
AICC (smaller is better)	92.2
BIC (smaller is better)	94.3

Null Model Likelihood Ratio Test

DF	Chi-Square	Pr > ChiSq
1	0.90	0.3420

Type 3 Tests of Fixed Effects

Effect	Num DF	Den DF	F Value	Pr > F
A	1	20	334.82	<.0001
B	1	20	137.42	<.0001
A*B	1	20	5.73	0.0266
time	2	40	177.90	<.0001
A*time	2	40	3.66	0.0348
B*time	2	40	3.05	0.0583
A*B*time	2	40	3.17	0.0526

Least Squares Means

Effect	A	B	Estimate	Standard Error	DF	t Value	Pr > |t|
A	P00		8.1600	0.06189	20	131.84	<.0001
A	P25		6.5583	0.06189	20	105.96	<.0001
B		S00	6.8461	0.06189	20	110.61	<.0001
B		S20	7.8722	0.06189	20	127.19	<.0001
A*B	P00	S00	7.5422	0.08753	20	86.17	<.0001
A*B	P00	S20	8.7778	0.08753	20	100.28	<.0001
A*B	P25	S00	6.1500	0.08753	20	70.26	<.0001
A*B	P25	S20	6.9667	0.08753	20	79.59	<.0001

The Mixed Procedure

Differences of Least Squares Means

Effect	A	B	_A	_B	Estimate	Standard Error	DF	t Value	Pr > |t|	Adjustment	Adj P
A	P00		P25		1.6017	0.08753	20	18.30	<.0001	Tukey	<.0001
B		S00		S20	-1.0261	0.08753	20	-11.72	<.0001	Tukey	<.0001
A*B	P00	S00	P00	S20	-1.2356	0.1238	20	-9.98	<.0001	Tukey	<.0001
A*B	P00	S00	P25	S00	1.3922	0.1238	20	11.25	<.0001	Tukey	<.0001
A*B	P00	S00	P25	S20	0.5756	0.1238	20	4.65	0.0002	Tukey	0.0008
A*B	P00	S20	P25	S00	2.6278	0.1238	20	21.23	<.0001	Tukey	<.0001
A*B	P00	S20	P25	S20	1.8111	0.1238	20	14.63	<.0001	Tukey	<.0001
A*B	P25	S00	P25	S20	-0.8167	0.1238	20	-6.60	<.0001	Tukey	<.0001

The Mixed Procedure

Model Information

Data Set	WORK.RUMEN
Dependent Variable	NH
Covariance Structure	Compound Symmetry
Subject Effect	ID(A*B)
Estimation Method	REML
Residual Variance Method	Profile
Fixed Effects SE Method	Prasad-Rao-Jeske-Kackar-Harville
Degrees of Freedom Method	Kenward-Roger

Class Level Information

Class	Levels	Values
ID	24	1 2 3 4 5 6 7 8 9 10 11 12 13 14 15 16 17 18 19 20 21 22 23 24
A	2	P00 P25
B	2	S00 S20
time	3	0 3 6

Dimensions

Covariance Parameters	2
Columns in X	36
Columns in Z	0
Subjects	24
Max Obs Per Subject	3
Observations Used	72
Observations Not Used	0
Total Observations	72

Iteration History

Iteration	Evaluations	-2 Res Log Like	Criterion
0	1	124.18316829	
1	1	123.18419980	0.00000000

Convergence criteria met.

The Mixed Procedure

Covariance Parameter Estimates

Cov Parm	Subject	Estimate
CS	ID(A*B)	0.04328
Residual		0.2809

Fit Statistics

-2 Res Log Likelihood	123.2
AIC (smaller is better)	127.2
AICC (smaller is better)	127.4
BIC (smaller is better)	129.5

Null Model Likelihood Ratio Test

DF	Chi-Square	Pr > ChiSq
1	1.00	0.3176

Type 3 Tests of Fixed Effects

Effect	Num DF	Den DF	F Value	Pr > F
A	1	20	240.31	<.0001
B	1	20	76.04	<.0001
A*B	1	20	7.59	0.0122
time	2	40	248.57	<.0001
A*time	2	40	0.73	0.4902
B*time	2	40	0.89	0.4166
A*B*time	2	40	0.74	0.4813

Least Squares Means

Effect	A	B	Estimate	Standard Error	DF	t Value	Pr > |t|
A	P00		18.6156	0.1068	20	174.28	<.0001
A	P25		16.2739	0.1068	20	152.36	<.0001
B		S00	16.7861	0.1068	20	157.15	<.0001
B		S20	18.1033	0.1068	20	169.49	<.0001
A*B	P00	S00	17.7489	0.1511	20	117.50	<.0001
A*B	P00	S20	19.4822	0.1511	20	128.97	<.0001
A*B	P25	S00	15.8233	0.1511	20	104.75	<.0001
A*B	P25	S20	16.7244	0.1511	20	110.72	<.0001

The Mixed Procedure

Differences of Least Squares Means

Effect	A	B	_A	_B	Estimate	Standard Error	DF	t Value	Pr > |t|	Adjustment	Adj P
A	P00		P25		2.3417	0.1511	20	15.50	<.0001	Tukey	<.0001
B		S00		S20	-1.3172	0.1511	20	-8.72	<.0001	Tukey	<.0001
A*B	P00	S00	P00	S20	-1.7333	0.2136	20	-8.11	<.0001	Tukey	<.0001
A*B	P00	S00	P25	S00	1.9256	0.2136	20	9.01	<.0001	Tukey	<.0001
A*B	P00	S00	P25	S20	1.0244	0.2136	20	4.80	0.0001	Tukey	0.0006
A*B	P00	S20	P25	S00	3.6589	0.2136	20	17.13	<.0001	Tukey	<.0001
A*B	P00	S20	P25	S20	2.7578	0.2136	20	12.91	<.0001	Tukey	<.0001
A*B	P25	S00	P25	S20	-0.9011	0.2136	20	-4.22	0.0004	Tukey	0.0022

The Mixed Procedure

Model Information

Data Set	WORK.RUMEN
Dependent Variable	PH
Covariance Structure	Compound Symmetry
Subject Effect	ID(A*B)
Estimation Method	REML
Residual Variance Method	Profile
Fixed Effects SE Method	Prasad-Rao-Jeske-Kackar-Harville
Degrees of Freedom Method	Kenward-Roger

Class Level Information

Class	Levels	Values
ID	24	1 2 3 4 5 6 7 8 9 10 11 12 13 14 15 16 17 18 19 20 21 22 23 24
A	2	P00 P25
B	2	S00 S20
time	3	0 3 6

Dimensions

Covariance Parameters	2
Columns in X	36
Columns in Z	0
Subjects	24
Max Obs Per Subject	3
Observations Used	72
Observations Not Used	0
Total Observations	72

Iteration History

Iteration	Evaluations	-2 Res Log Like	Criterion
0	1	-116.13796748	
1	1	-118.24151536	0.00000000

Convergence criteria met.

The Mixed Procedure

Covariance Parameter Estimates

Cov Parm	Subject	Estimate
CS	ID(A*B)	0.001155
Residual		0.004750

Fit Statistics

-2 Res Log Likelihood	-118.2
AIC (smaller is better)	-114.2
AICC (smaller is better)	-114.0
BIC (smaller is better)	-111.9

Null Model Likelihood Ratio Test

DF	Chi-Square	Pr > ChiSq
1	2.10	0.1470

Type 3 Tests of Fixed Effects

Effect	Num DF	Den DF	F Value	Pr > F
A	1	20	27.32	<.0001
B	1	20	9.90	0.0051
A*B	1	20	1.43	0.2456
time	2	40	321.96	<.0001
A*time	2	40	1.63	0.2089
B*time	2	40	0.18	0.8380
A*B*time	2	40	1.41	0.2558

Least Squares Means

Effect	A	B	Estimate	Standard Error	DF	t Value	Pr > |t|
A	P00		6.4008	0.01511	20	423.70	<.0001
A	P25		6.5125	0.01511	20	431.09	<.0001
B		S00	6.4903	0.01511	20	429.62	<.0001
B		S20	6.4231	0.01511	20	425.17	<.0001
A*B	P00	S00	6.4217	0.02136	20	300.57	<.0001
A*B	P00	S20	6.3800	0.02136	20	298.62	<.0001
A*B	P25	S00	6.5589	0.02136	20	307.00	<.0001
A*B	P25	S20	6.4661	0.02136	20	302.65	<.0001

The Mixed Procedure

Differences of Least Squares Means

Effect	A	B	_A	_B	Estimate	Standard Error	DF	t Value	Pr > |t|	Adjustment	Adj P
A	P00		P25		-0.1117	0.02136	20	-5.23	<.0001	Tukey	<.0001
B		S00		S20	0.06722	0.02136	20	3.15	0.0051	Tukey	0.0051
A*B	P00	S00	P00	S20	0.04167	0.03021	20	1.38	0.1831	Tukey	0.5262
A*B	P00	S00	P25	S00	-0.1372	0.03021	20	-4.54	0.0002	Tukey	0.0011
A*B	P00	S00	P25	S20	-0.04444	0.03021	20	-1.47	0.1569	Tukey	0.4725
A*B	P00	S20	P25	S00	-0.1789	0.03021	20	-5.92	<.0001	Tukey	<.0001
A*B	P00	S20	P25	S20	-0.08611	0.03021	20	-2.85	0.0099	Tukey	0.0450
A*B	P25	S00	P25	S20	0.09278	0.03021	20	3.07	0.0060	Tukey	0.0284
