# Supplementary material for: Influence of substituting 25% alfalfa hay with Panicum maximum cv. Mombasa with or without spirulina supplementation on the productive performance of fattening Barki lambs
Source: Sci Rep. 2026 Jan 10;16:1347. doi: 10.1038/s41598-025-28525-1 (PMC12796356; doi:10.1038/s41598-025-28525-1)
Supplement: Supplementary file 1 — Supplementary Material 1 [file 41598_2025_28525_MOESM1_ESM.zip › Meteab_Supplementary/Raw Data/fermentation repeated Meteab.sas.pdf]

```
Data Rumen;
 Input A$      B$      time       ID       VFA      NH
PH;
Cards;
P00      S00      0      1      5.60      15.89      6.80
P00      S00      0      2      6.65      16.15      6.73
P00      S00      0      3      5.75      15.32      6.75
P00      S00      0      4      6.66      16.05      6.67
P00      S00      0      5      5.45      16.11      6.66
P00      S00      0      6      6.55      16.38      6.60
P00      S00      3      1      8.40      19.77      6.31
P00      S00      3      2      8.50      18.48      6.12
P00      S00      3      3      8.40      19.24      6.29
P00      S00      3      4      9.30      19.67      6.14
P00      S00      3      5      9.80      19.48      6.15
P00      S00      3      6      8.60      19.74      6.27
P00      S00      6      1      7.90      17.57      6.46
P00      S00      6      2      6.90      17.44      6.35
P00      S00      6      3      7.60      17.79      6.32
P00      S00      6      4      7.80      17.04      6.30
P00      S00      6      5      7.90      18.68      6.31
P00      S00      6      6      8.00      18.68      6.36
P00      S20      0      7      7.80      17.87      6.62
P00      S20      0      8      7.50      18.16      6.75
P00      S20      0      9      6.60      18.50      6.66
P00      S20      0      10      6.30      18.87
6.71
P00      S20      0      11      7.20      17.04
6.62
P00      S20      0      12      7.90      16.86
6.65
```

P00	S20	3	7	9.40	21.04	6.12
P00	S20	3	8	10.10	20.67	6.23
P00	S20	3	9	10.00	21.03	6.25
P00	S20	3	10	10.20	22.78	6.09
P00	S20	3	11	10.40	21.83	6.12
P00	S20	3	12	9.90	21.03	6.06
P00	S20	6	7	8.80	19.04	6.23
P00	S20	6	8	9.40	19.13	6.40
P00	S20	6	9	8.80	19.33	6.40
P00	S20	6	10	9.70	19.04	6.30
P00	S20	6	11	8.70	19.13	6.30
P00	S20	6	12	9.30	19.33	6.33
P25	S00	0	13	5.20	14.47	6.85
P25	S00	0	14	5.40	13.93	6.72
P25	S00	0	15	5.20	14.02	6.75
P25	S00	0	16	5.35	14.47	6.88
P25	S00	0	17	5.45	13.93	6.84

P25	S00	0	18	5.30	14.02	6.85
P25	S00	3	13	7.00	17.50	6.40
P25	S00	3	14	7.00	17.79	6.29
P25	S00	3	15	6.50	17.30	6.28
P25	S00	3	16	6.90	17.57	6.31
P25	S00	3	17	6.50	16.44	6.29
P25	S00	3	18	6.90	17.79	6.24
P25	S00	6	13	6.30	15.60	6.61
P25	S00	6	14	6.70	15.56	6.59
P25	S00	6	15	6.30	16.09	6.47
P25	S00	6	16	6.50	16.28	6.65
P25	S00	6	17	6.30	16.08	6.45
P25	S00	6	18	5.90	15.98	6.59
P25	S20	0	19	5.30	14.32	6.71
P25	S20	0	20	5.60	14.05	6.72

P25	S20	0	21	5.80	15.96	6.80
P25	S20	0	22	5.70	15.32	6.70
P25	S20	0	23	5.90	14.05	6.60
P25	S20	0	24	5.30	15.96	6.82
P25	S20	3	19	8.70	18.47	5.98
P25	S20	3	20	8.60	19.03	6.33
P25	S20	3	21	8.50	18.21	6.25
P25	S20	3	22	7.80	17.67	6.38
P25	S20	3	23	8.30	18.03	6.34
P25	S20	3	24	7.90	19.01	6.23
P25	S20	6	19	7.80	16.93	6.46
P25	S20	6	20	6.70	16.44	6.47
P25	S20	6	21	6.40	17.79	6.41
P25	S20	6	22	7.50	16.57	6.39
P25	S20	6	23	6.90	16.44	6.38

```
P25      S20      6      24      6.70      16.79
6.42
;
proc mixed data=Rumen;
class ID A B Time;
model VFA = A|B|Time / ddfm=kr;
repeated Time / subject=ID(A*B) type=cs;
lsmeans A B A*B / pdiff=all adjust=tukey;
run;


proc mixed data=Rumen;
class ID A B Time;
model NH= A|B|Time / ddfm=kr;
repeated Time / subject=ID(A*B) type=cs;
lsmeans A B A*B / pdiff=all adjust=tukey;
run;


proc mixed data=Rumen;
class ID A B Time;
model PH = A|B|Time / ddfm=kr;
repeated Time / subject=ID(A*B) type=cs;
lsmeans A B A*B / pdiff=all adjust=tukey;
run;
```
