# Supplementary material for: Influence of substituting 25% alfalfa hay with Panicum maximum cv. Mombasa with or without spirulina supplementation on the productive performance of fattening Barki lambs
Source: Sci Rep. 2026 Jan 10;16:1347. doi: 10.1038/s41598-025-28525-1 (PMC12796356; doi:10.1038/s41598-025-28525-1)
Supplement: Supplementary file 1 — Supplementary Material 1 [file 41598_2025_28525_MOESM1_ESM.zip › Meteab_Supplementary/Raw Data/Nitrogen fattening two ways in data.sas.pdf]

```
Data Nitrogen balanc fattening two;
Input P$ S$ TNi  FN    UN    TNE   NB ;
Cards;
P00   S00   49.05 6.86  33.69 40.55 8.50
P00   S00   49.05 7.21  32.52 39.73 9.31
P00   S00   49.05 7.45  32.84 40.30 8.75
P00   S00   49.05 7.80  32.21 40.01 9.04
P00   S00   49.05 7.23  32.69 39.93 9.12
P00   S00   49.05 7.31  32.71 40.02 9.03
P00   S20   50.31 4.87  35.47 40.34 9.97
P00   S20   50.31 6.32  34.18 40.50 9.81
P00   S20   50.31 6.52  34.23 40.75 9.56
P00   S20   50.31 5.85  34.46 40.32 9.99
P00   S20   50.31 6.09  34.39 40.48 9.82
P00   S20   50.31 5.50  35.45 40.95 9.36
P25   S00   44.17 9.24  28.11 37.35 6.82
P25   S00   44.17 9.30  29.03 38.33 5.84
P25   S00   44.17 9.34  27.99 37.33 6.83
P25   S00   44.17 9.32  26.87 36.19 7.98
P25   S00   44.17 9.14  28.68 37.81 6.36
P25   S00   44.17 9.45  26.92 36.37 7.80
P25   S20   46.08 7.90  29.59 37.49 8.59
P25   S20   46.08 8.16  29.74 37.90 8.17
P25   S20   46.08 7.38  31.26 38.64 7.44
P25   S20   46.08 8.80  28.32 37.12 8.96
P25   S20   46.08 7.38  29.93 37.32 8.76
P25   S20   46.08 7.58  29.73 37.31 8.77
;
Proc GLM;
Class P S;
Model   TNi FN    UN    TNE   NB = P S P*S ;
```

```
MEANS P S / duncan;
LSMEANS P S P*S  / STDERR;
PROC MEANS STD; VAR  TNi     FN    UN    TNE   NB;
RUN;
```
