# Supplementary material for: Influence of substituting 25% alfalfa hay with Panicum maximum cv. Mombasa with or without spirulina supplementation on the productive performance of fattening Barki lambs
Source: Sci Rep. 2026 Jan 10;16:1347. doi: 10.1038/s41598-025-28525-1 (PMC12796356; doi:10.1038/s41598-025-28525-1)
Supplement: Supplementary file 1 — Supplementary Material 1 [file 41598_2025_28525_MOESM1_ESM.zip › Meteab_Supplementary/Raw Data/Nitrogen fattening two ways.pdf]

The GLM Procedure

Class Level Information

Class	Levels	Values
P	2	P00 P25
S	2	S00 S20

Number of observations 24

The GLM Procedure

Dependent Variable: TNi

Source	DF	Sum of Squares	Mean Square	F Value	Pr > F
Model	3	140.1952500	46.7317500	Infty	<.0001
Error	20	0.0000000	0.0000000		
Corrected Total	23	140.1952500			

R-Square	Coeff Var	Root MSE	TNi Mean
1.000000	0	0	47.40250

Source	DF	Type I SS	Mean Square	F Value	Pr > F
P	1	124.4881500	124.4881500	Infty	<.0001
S	1	15.0733500	15.0733500	Infty	<.0001
P*S	1	0.6337500	0.6337500	Infty	<.0001

Source	DF	Type III SS	Mean Square	F Value	Pr > F
P	1	124.4881500	124.4881500	Infty	<.0001
S	1	15.0733500	15.0733500	Infty	<.0001

P*S	1	0.6337500	0.6337500	Infty	<.0001

The GLM Procedure

Dependent Variable: FN

Source	DF	Sum of Squares	Mean Square	F Value	Pr > F
Model	3	36.43103333	12.14367778	62.98	<.0001
Error	20	3.85630000	0.19281500		
Corrected Total	23	40.28733333			

R-Square	Coeff Var	Root MSE	FN Mean
0.904280	5.790423	0.439107	7.583333

Source	DF	Type I SS	Mean Square	F Value	Pr > F
P	1	23.96001667	23.96001667	124.26	<.0001
S	1	12.47041667	12.47041667	64.68	<.0001
P*S	1	0.00060000	0.00060000	0.00	0.9561

Source	DF	Type III SS	Mean Square	F Value	Pr > F
P	1	23.96001667	23.96001667	124.26	<.0001
S	1	12.47041667	12.47041667	64.68	<.0001
P*S	1	0.00060000	0.00060000	0.00	0.9561

The GLM Procedure

Dependent Variable: UN

Source	DF	Sum of Squares	Mean Square	F Value	Pr > F
Model	3	164.5113125	54.8371042	96.44	<.0001
Error	20	11.3722833	0.5686142		

Corrected Total	23	175.8835958

R-Square	Coeff Var	Root MSE	UN Mean
0.935342	2.409763	0.754065	31.29208

Source	DF	Type I SS	Mean Square	F Value	Pr > F
P	1	143.4237042	143.4237042	252.23	<.0001
S	1	21.0750042	21.0750042	37.06	<.0001
P*S	1	0.0126042	0.0126042	0.02	0.8831

Source	DF	Type III SS	Mean Square	F Value	Pr > F
P	1	143.4237042	143.4237042	252.23	<.0001
S	1	21.0750042	21.0750042	37.06	<.0001
P*S	1	0.0126042	0.0126042	0.02	0.8831

The GLM Procedure

Dependent Variable: TNE

Source	DF	Sum of Squares	Mean Square	F Value	Pr > F
Model	3	51.36160000	17.12053333	60.18	<.0001
Error	20	5.68953333	0.28447667		
Corrected Total	23	57.05113333			

R-Square	Coeff Var	Root MSE	TNE Mean
0.900273	1.371937	0.533364	38.87667

Source	DF	Type I SS	Mean Square	F Value	Pr > F
P	1	50.22826667	50.22826667	176.56	<.0001
S	1	1.12666667	1.12666667	3.96	0.0604

P*S	1	0.00666667	0.00666667	0.02	0.8799

Source	DF	Type III SS	Mean Square	F Value	Pr > F
P	1	50.22826667	50.22826667	176.56	<.0001
S	1	1.12666667	1.12666667	3.96	0.0604
P*S	1	0.00666667	0.00666667	0.02	0.8799

The GLM Procedure

Dependent Variable: NB

Source	DF	Sum of Squares	Mean Square	F Value	Pr > F
Model	3	25.29525000	8.43175000	29.64	<.0001
Error	20	5.68853333	0.28442667		
Corrected Total	23	30.98378333			

R-Square	Coeff Var	Root MSE	NB Mean
0.816403	6.256526	0.533317	8.524167

Source	DF	Type I SS	Mean Square	F Value	Pr > F
P	1	16.56681667	16.56681667	58.25	<.0001
S	1	7.95801667	7.95801667	27.98	<.0001
P*S	1	0.77041667	0.77041667	2.71	0.1154

Source	DF	Type III SS	Mean Square	F Value	Pr > F
P	1	16.56681667	16.56681667	58.25	<.0001
S	1	7.95801667	7.95801667	27.98	<.0001
P*S	1	0.77041667	0.77041667	2.71	0.1154

The GLM Procedure

Duncan's Multiple Range Test for TNi

NOTE: This test controls the Type I comparisonwise error rate, not the experimentwise error rate.

**Alpha**	**0.05**
**Error Degrees of Freedom**	**20**
**Error Mean Square**	**0**

**Number of Means**	**2**
**Critical Range**	**0**

Means with the same letter are not significantly different.

Duncan Grouping	Mean	N	P
A	49.68	12	P00
B	45.13	12	P25

The GLM Procedure

Duncan's Multiple Range Test for FN

NOTE: This test controls the Type I comparisonwise error rate, not the experimentwise error rate.

**Alpha**	**0.05**
**Error Degrees of Freedom**	**20**
**Error Mean Square**	**0.192815**

**Number of Means**	**2**
**Critical Range**	**.3739**

Means with the same letter are not significantly different.

Duncan Grouping	Mean	N	P
A	8.5825	12	P25
B	6.5842	12	P00

The GLM Procedure

Duncan's Multiple Range Test for UN

NOTE: This test controls the Type I comparisonwise error rate, not the experimentwise error rate.

Alpha	0.05
Error Degrees of Freedom	20
Error Mean Square	0.568614

Number of Means	2
Critical Range	.6422

Means with the same letter are not significantly different.

Duncan Grouping	Mean	N	P
A	33.7367	12	P00
B	28.8475	12	P25

The GLM Procedure

Duncan's Multiple Range Test for TNE

NOTE: This test controls the Type I comparisonwise error rate, not the experimentwise error rate.

Alpha	0.05
Error Degrees of Freedom	20

Error Mean Square 0.284477

Number of Means 2
Critical Range .4542

Means with the same letter are not significantly different.

Duncan Grouping	Mean	N	P
A	40.3233	12	P00
B	37.4300	12	P25

The GLM Procedure

Duncan's Multiple Range Test for NB

NOTE: This test controls the Type I comparisonwise error rate, not the experimentwise error rate.

Alpha 0.05
Error Degrees of Freedom 20
Error Mean Square 0.284427

Number of Means 2
Critical Range .4542

Means with the same letter are not significantly different.

Duncan Grouping	Mean	N	P
A	9.3550	12	P00
B	7.6933	12	P25

The GLM Procedure

Duncan's Multiple Range Test for TNi

NOTE: This test controls the Type I comparisonwise error rate, not the experimentwise error rate.

Alpha	0.05
Error Degrees of Freedom	20
Error Mean Square	0

Number of Means	2
Critical Range	0

Means with the same letter are not significantly different.

Duncan Grouping	Mean	N	S
A	48.20	12	S20
B	46.61	12	S00

The GLM Procedure

Duncan's Multiple Range Test for FN

NOTE: This test controls the Type I comparisonwise error rate, not the experimentwise error rate.

Alpha	0.05
Error Degrees of Freedom	20
Error Mean Square	0.192815

Number of Means	2
Critical Range	.3739

Means with the same letter are not significantly different.

Duncan Grouping	Mean	N	S
A	8.3042	12	S00
B	6.8625	12	S20

The GLM Procedure

Duncan's Multiple Range Test for UN

NOTE: This test controls the Type I comparisonwise error rate, not the experimentwise error
rate.

Alpha	0.05
Error Degrees of Freedom	20
Error Mean Square	0.568614

Number of Means	2
Critical Range	.6422

Means with the same letter are not significantly different.

Duncan Grouping	Mean	N	S
A	32.2292	12	S20
B	30.3550	12	S00

The GLM Procedure

Duncan's Multiple Range Test for TNE

NOTE: This test controls the Type I comparisonwise error rate, not the experimentwise error
rate.

Alpha	0.05
Error Degrees of Freedom	20
Error Mean Square	0.284477

Number of Means	2
Critical Range	.4542

Means with the same letter are not significantly different.

Duncan Grouping	Mean	N	S
A	39.0933	12	S20
A			
A	38.6600	12	S00

The GLM Procedure

Duncan's Multiple Range Test for NB

NOTE: This test controls the Type I comparisonwise error rate, not the experimentwise error rate.

Alpha	0.05
Error Degrees of Freedom	20
Error Mean Square	0.284427

Number of Means	2
Critical Range	.4542

Means with the same letter are not significantly different.

Duncan Grouping	Mean	N	S
A	9.1000	12	S20
B	7.9483	12	S00

The GLM Procedure
Least Squares Means

P	TNi LSMEAN	Standard Error	Pr > |t|
P00	49.6800000	0.0000000	.
P25	45.1250000	0.0000000	.

P	FN LSMEAN	Standard Error	Pr > |t|
P00	6.58416667	0.12675929	<.0001
P25	8.58250000	0.12675929	<.0001

P	UN LSMEAN	Standard Error	Pr > |t|
P00	33.7366667	0.2176798	<.0001
P25	28.8475000	0.2176798	<.0001

P	TNE LSMEAN	Standard Error	Pr > |t|
P00	40.3233333	0.1539688	<.0001
P25	37.4300000	0.1539688	<.0001

P	NB LSMEAN	Standard Error	Pr > |t|
P00	9.35500000	0.15395526	<.0001
P25	7.69333333	0.15395526	<.0001

S	TNi LSMEAN	Standard Error	Pr > |t|
S00	46.6100000	0.0000000	.
S20	48.1950000	0.0000000	.

S	FN LSMEAN	Standard Error	Pr > |t|
S00	8.30416667	0.12675929	<.0001
S20	6.86250000	0.12675929	<.0001

The GLM Procedure
Least Squares Means

S	UN LSMEAN	Standard Error	Pr > |t|
S00	30.3550000	0.2176798	<.0001
S20	32.2291667	0.2176798	<.0001

S	TNE LSMEAN	Standard Error	Pr > |t|
S00	38.6600000	0.1539688	<.0001
S20	39.0933333	0.1539688	<.0001

S	NB LSMEAN	Standard Error	Pr > |t|
S00	7.94833333	0.15395526	<.0001
S20	9.10000000	0.15395526	<.0001

P	S	TNi LSMEAN	Standard Error	Pr > |t|
P00	S00	49.0500000	0.0000000	.
P00	S20	50.3100000	0.0000000	.
P25	S00	44.1700000	0.0000000	.
P25	S20	46.0800000	0.0000000	.

P	S	FN LSMEAN	Standard Error	Pr > |t|
P00	S00	7.31000000	0.17926470	<.0001
P00	S20	5.85833333	0.17926470	<.0001
P25	S00	9.29833333	0.17926470	<.0001

P25	S20	7.86666667	0.17926470	<.0001

P	S	UN LSMEAN	Standard Error	Pr > |t|
P00	S00	32.7766667	0.3078458	<.0001
P00	S20	34.6966667	0.3078458	<.0001
P25	S00	27.9333333	0.3078458	<.0001
P25	S20	29.7616667	0.3078458	<.0001

The GLM Procedure
Least Squares Means

P	S	TNE LSMEAN	Standard Error	Pr > |t|
P00	S00	40.0900000	0.2177448	<.0001
P00	S20	40.5566667	0.2177448	<.0001
P25	S00	37.2300000	0.2177448	<.0001
P25	S20	37.6300000	0.2177448	<.0001

P	S	NB LSMEAN	Standard Error	Pr > |t|
P00	S00	8.95833333	0.21772562	<.0001
P00	S20	9.75166667	0.21772562	<.0001
P25	S00	6.93833333	0.21772562	<.0001
P25	S20	8.44833333	0.21772562	<.0001

The MEANS Procedure

Variable	Std Dev
TNi	2.4688956
FN	1.3234890
UN	2.7653414
TNE	1.5749553
NB	1.1606554
