# Supplementary figures and images for: Influence of substituting 25% alfalfa hay with Panicum maximum cv. Mombasa with or without spirulina supplementation on the productive performance of fattening Barki lambs
Source: Sci Rep. 2026 Jan 10;16:1347. doi: 10.1038/s41598-025-28525-1 (PMC12796356; doi:10.1038/s41598-025-28525-1)

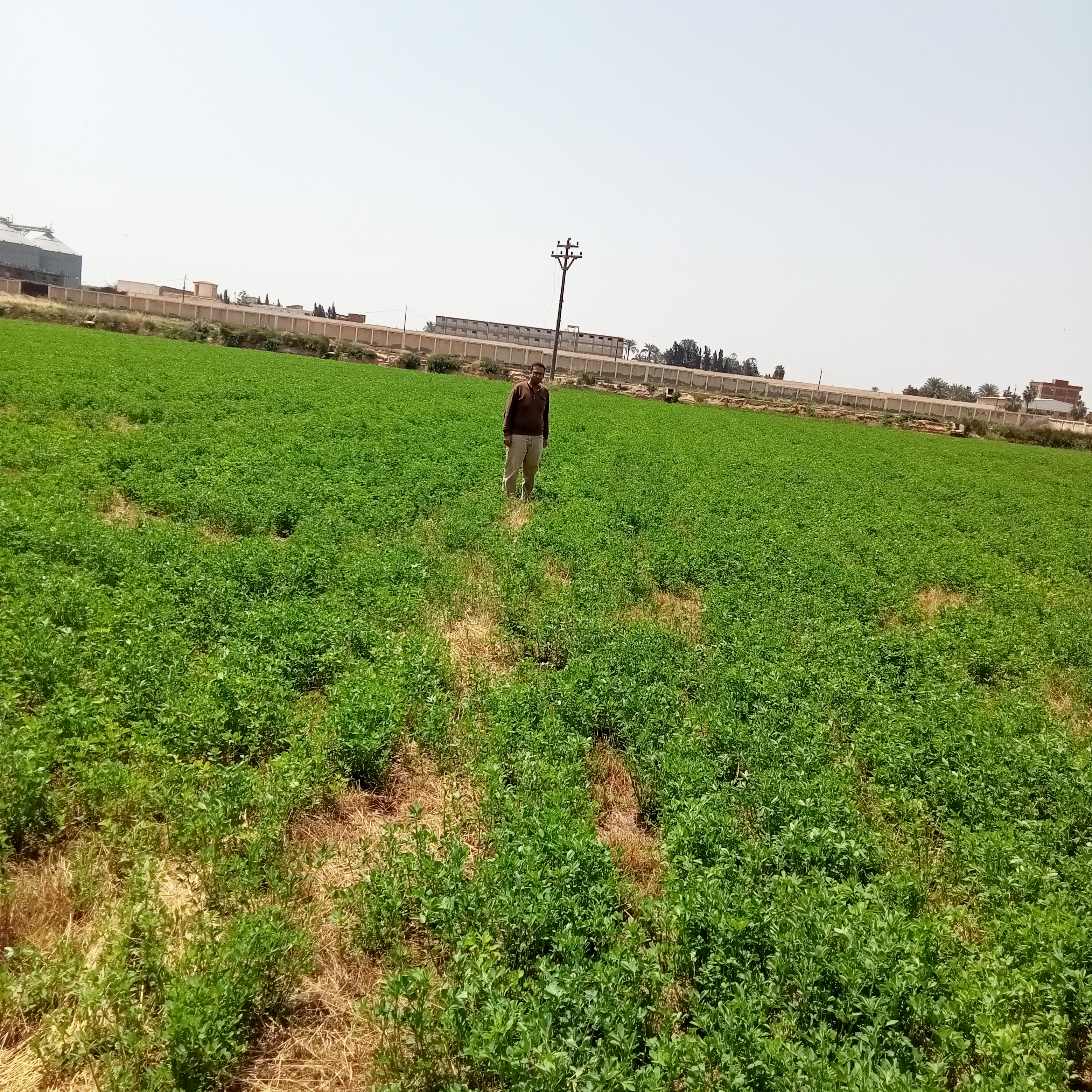

Supplement: Supplementary file 1 — Supplementary Material 1 [file 41598_2025_28525_MOESM1_ESM.zip › Meteab_Supplementary/photos/Alfalfa in Maryout Station.jpg]

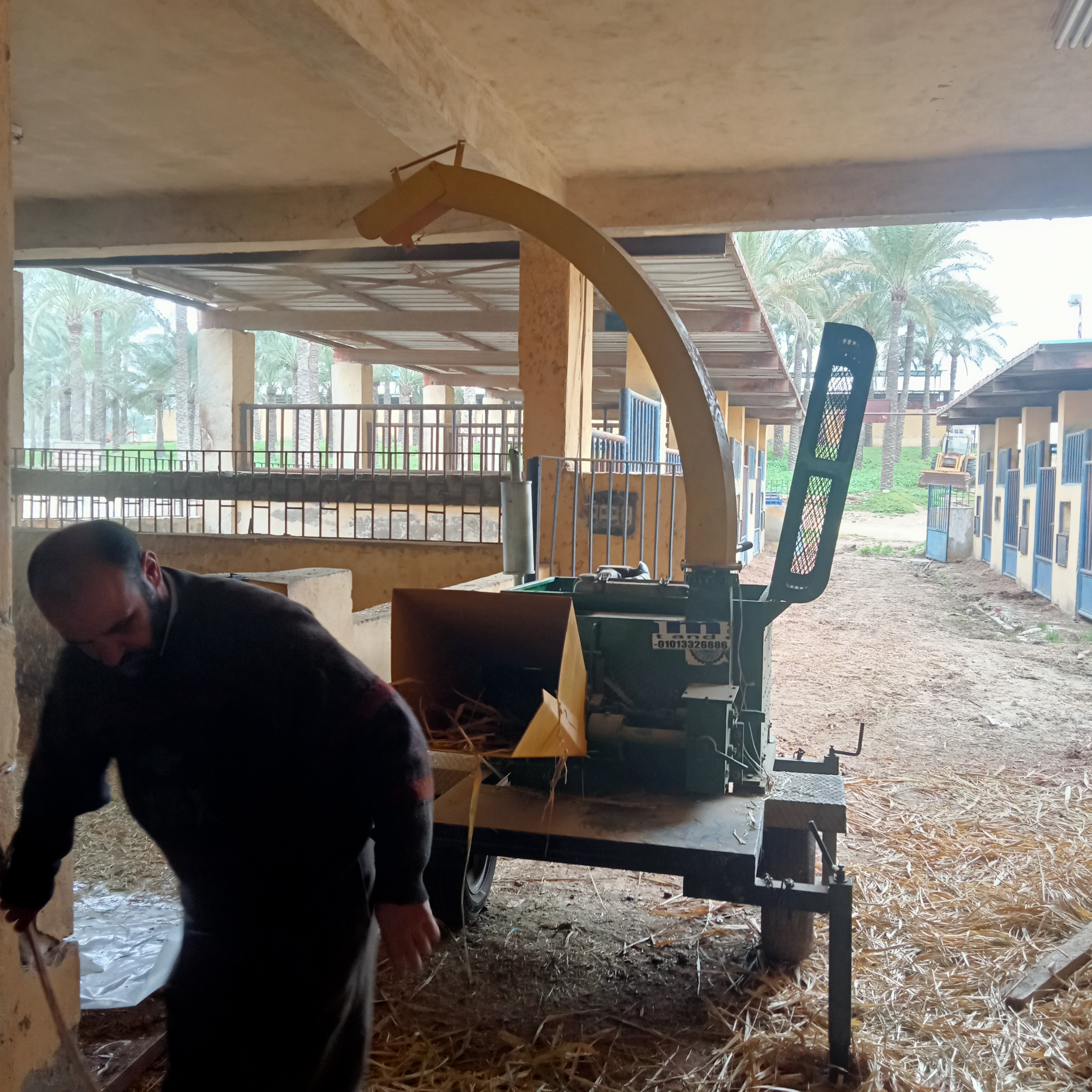

Supplement: Supplementary file 1 — Supplementary Material 1 [file 41598_2025_28525_MOESM1_ESM.zip › Meteab_Supplementary/photos/Fodder Cutter.jpg]

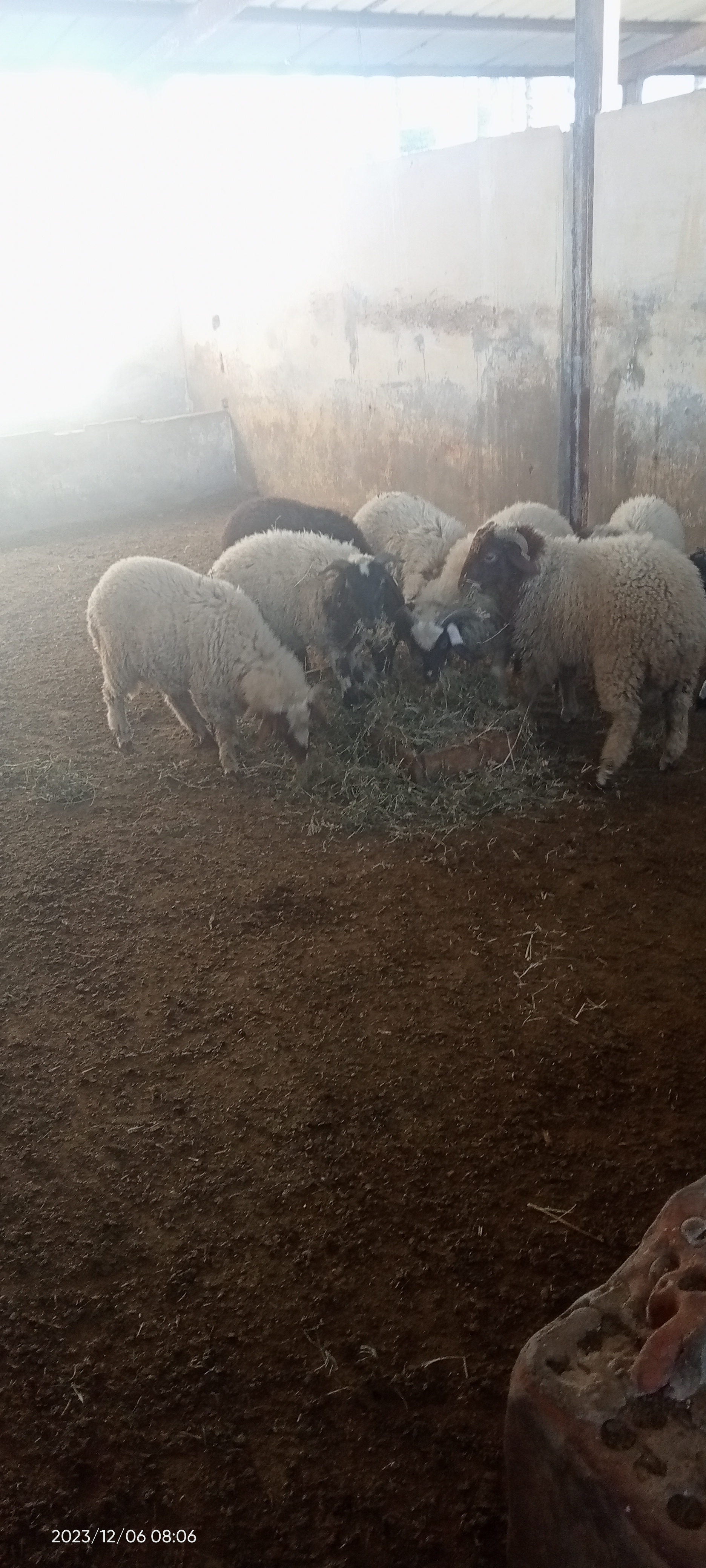

Supplement: Supplementary file 1 — Supplementary Material 1 [file 41598_2025_28525_MOESM1_ESM.zip › Meteab_Supplementary/photos/Lambs of experience.jpg]

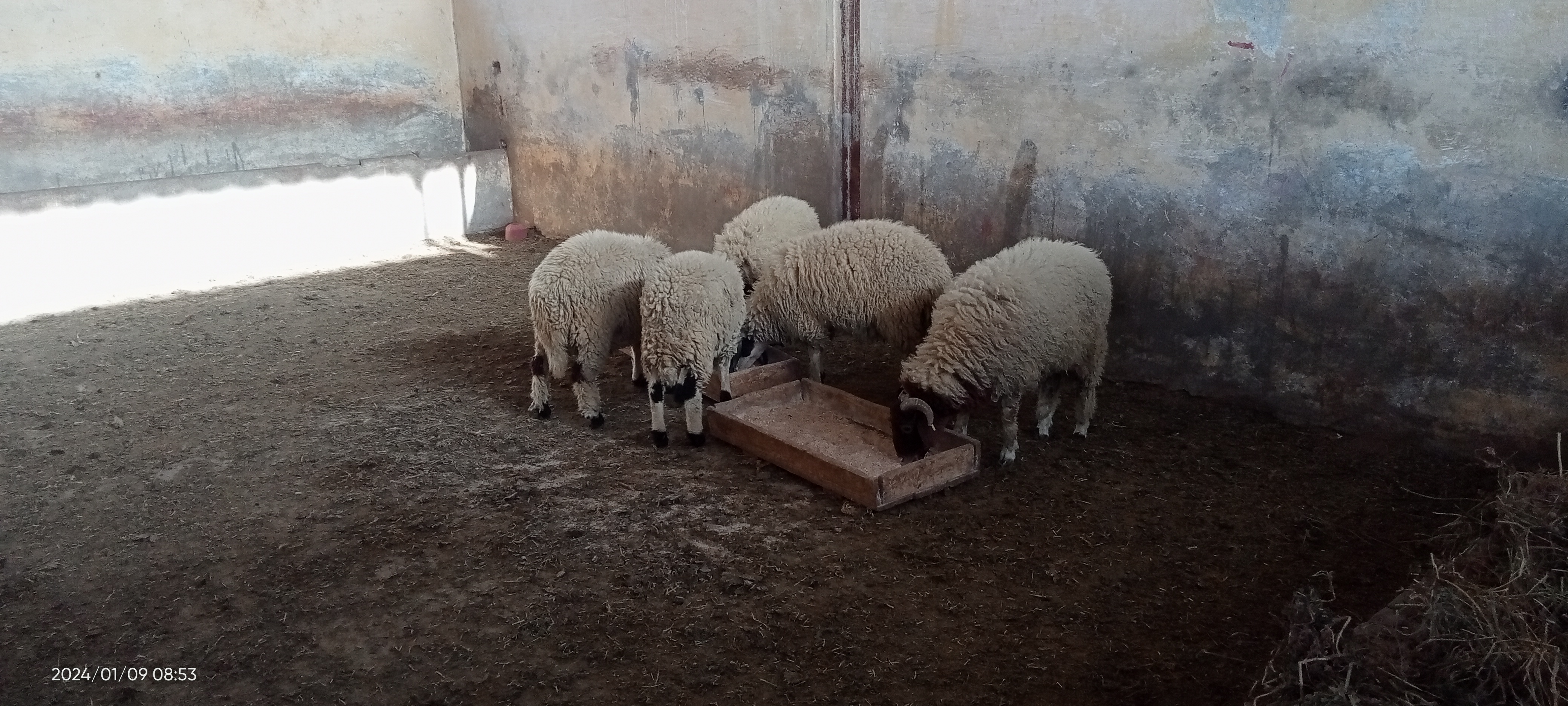

Supplement: Supplementary file 1 — Supplementary Material 1 [file 41598_2025_28525_MOESM1_ESM.zip › Meteab_Supplementary/photos/Lambs of experience2.jpg]

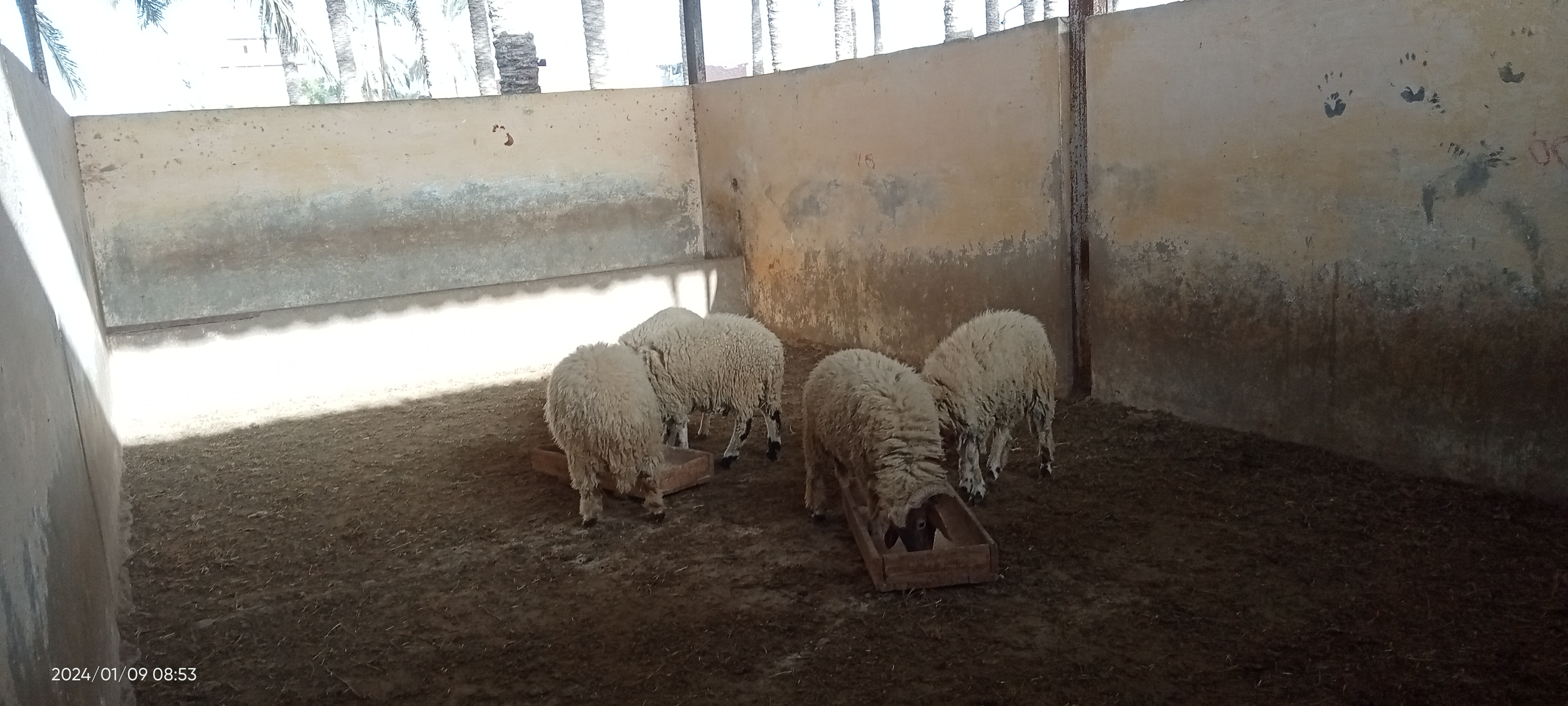

Supplement: Supplementary file 1 — Supplementary Material 1 [file 41598_2025_28525_MOESM1_ESM.zip › Meteab_Supplementary/photos/Lambs of experience3.jpg]

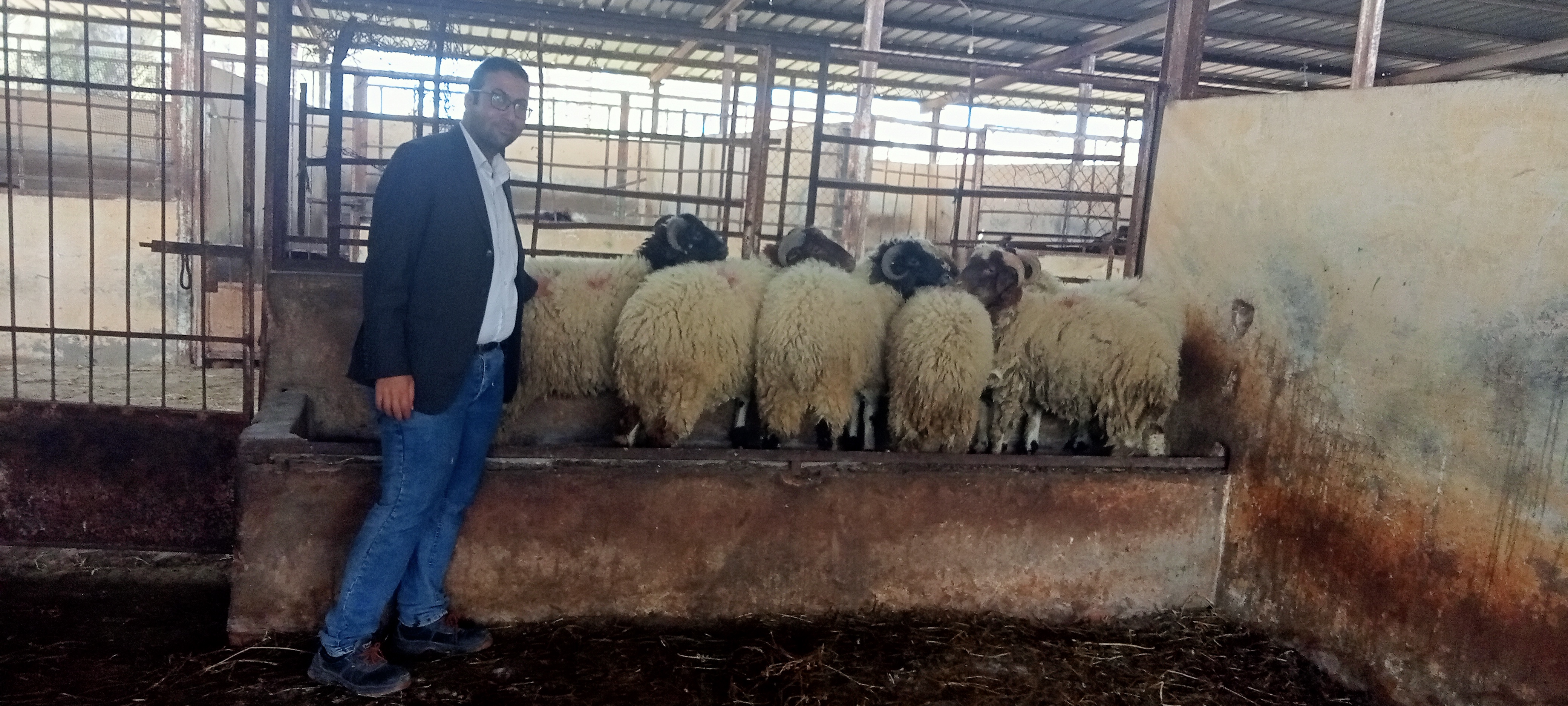

Supplement: Supplementary file 1 — Supplementary Material 1 [file 41598_2025_28525_MOESM1_ESM.zip › Meteab_Supplementary/photos/Lambs of experience4.jpg]

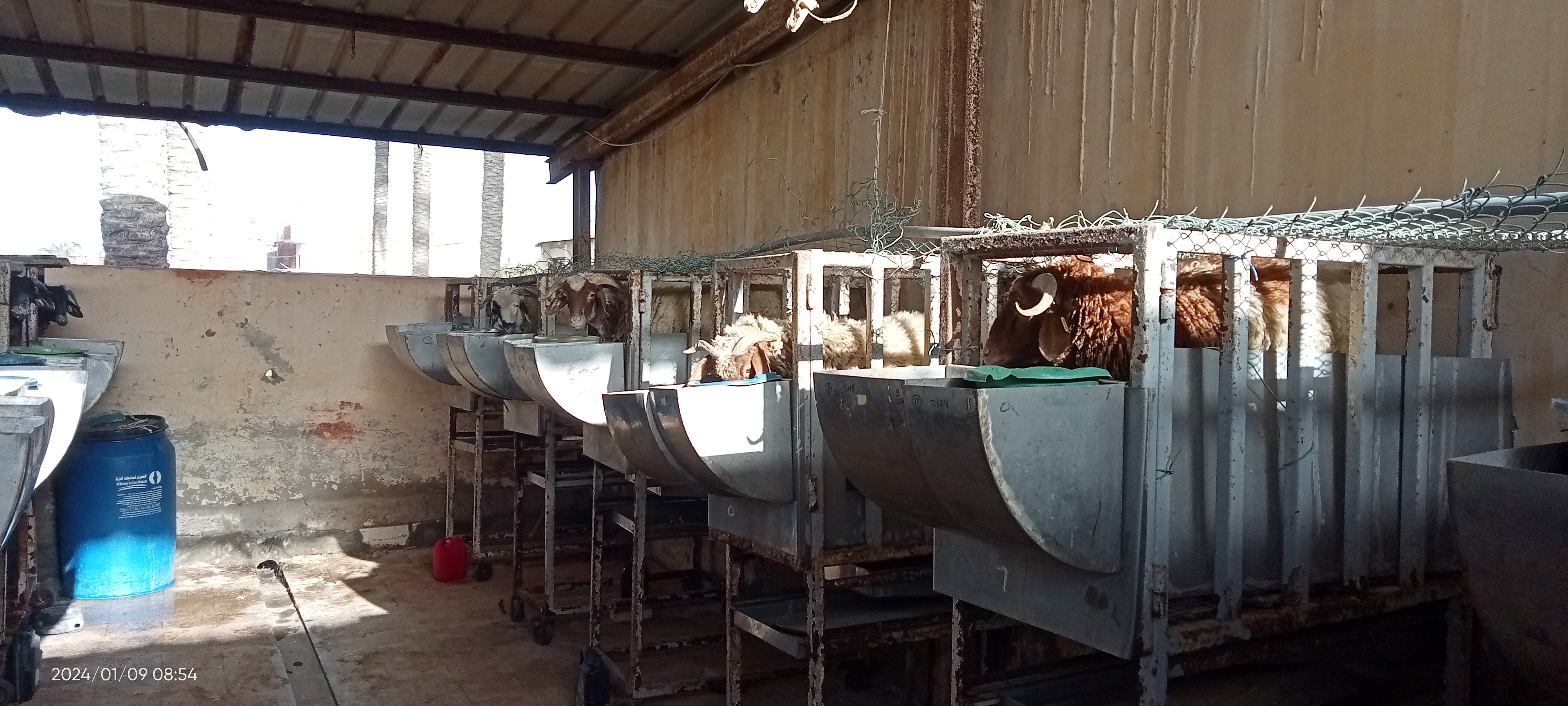

Supplement: Supplementary file 1 — Supplementary Material 1 [file 41598_2025_28525_MOESM1_ESM.zip › Meteab_Supplementary/photos/metabolic crates 2.jpg]

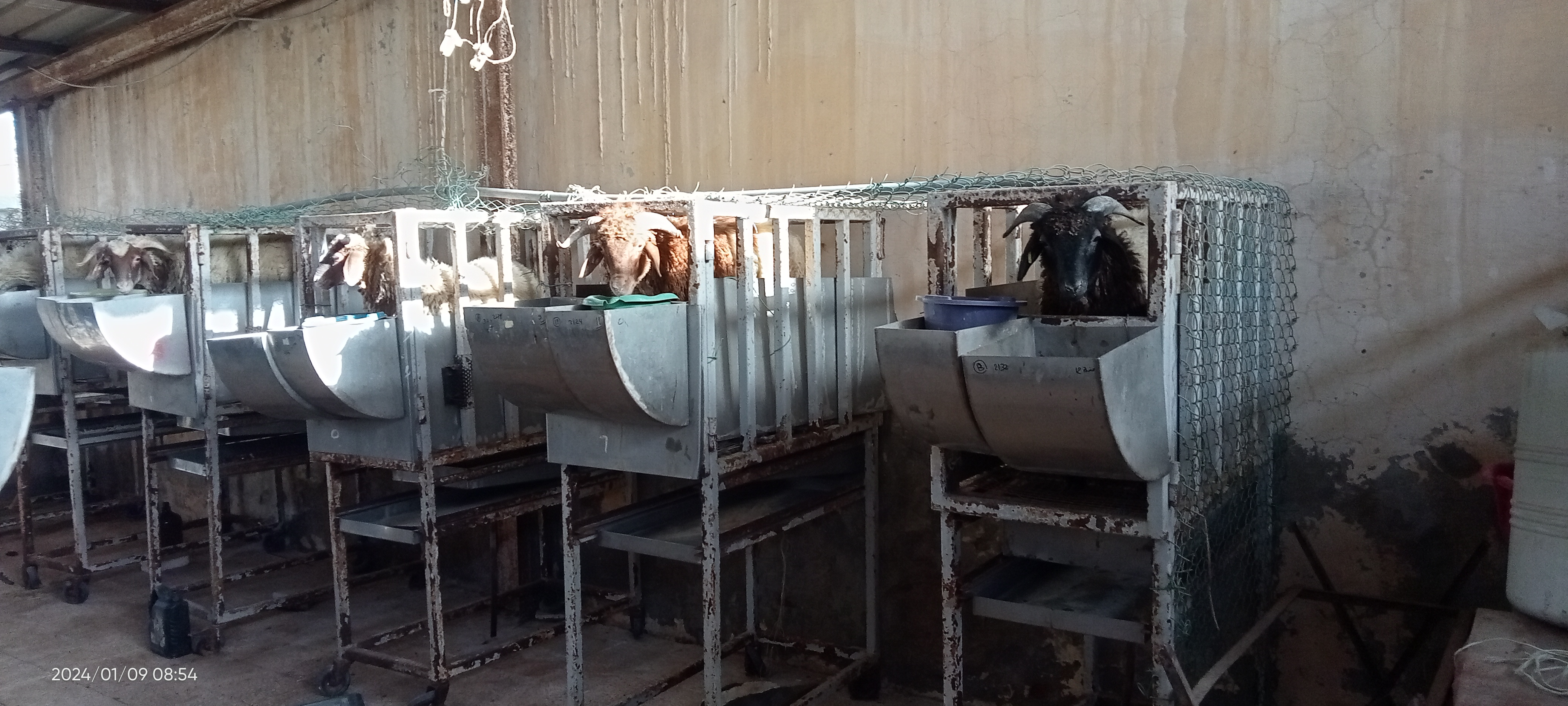

Supplement: Supplementary file 1 — Supplementary Material 1 [file 41598_2025_28525_MOESM1_ESM.zip › Meteab_Supplementary/photos/metabolic crates.jpg]

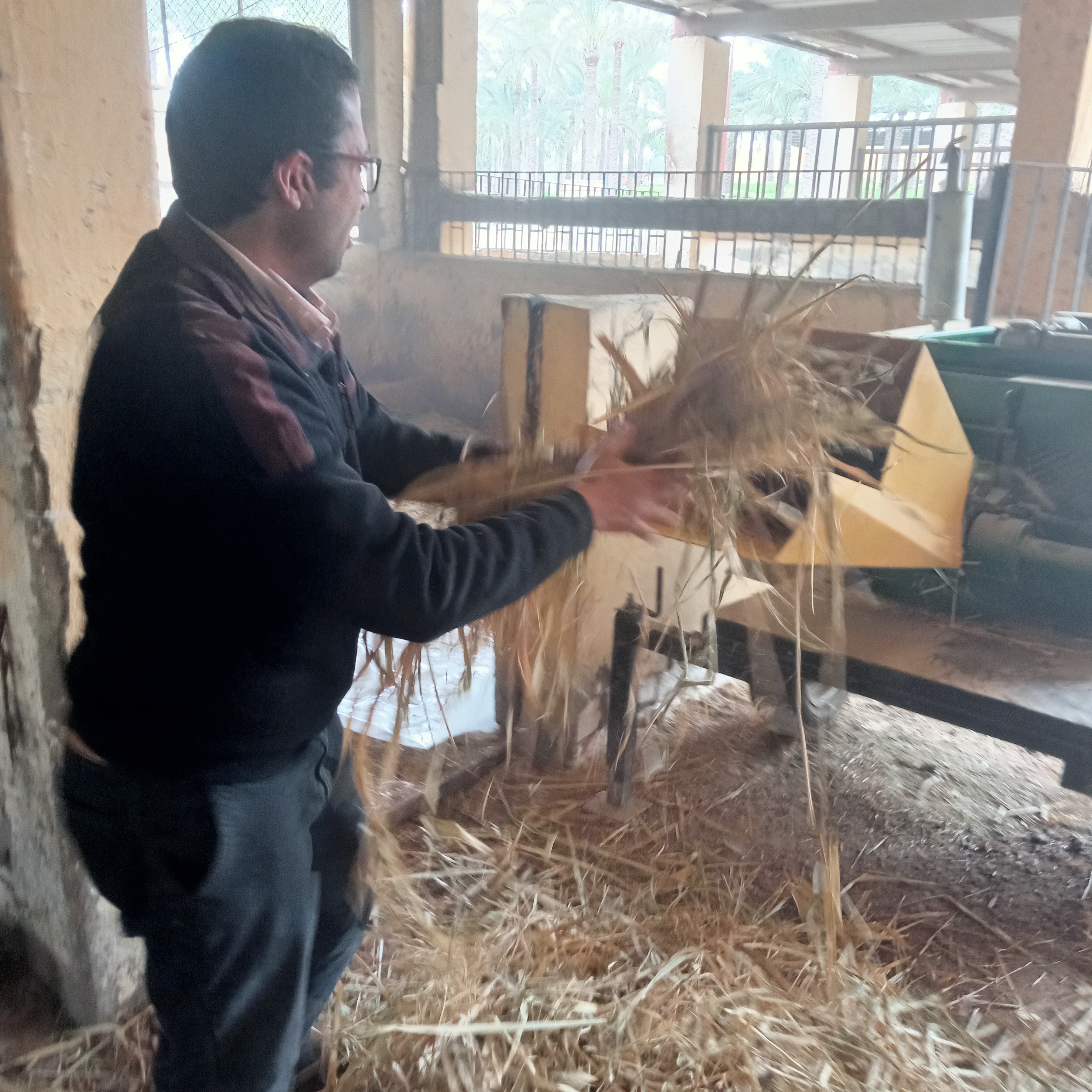

Supplement: Supplementary file 1 — Supplementary Material 1 [file 41598_2025_28525_MOESM1_ESM.zip › Meteab_Supplementary/photos/Panicum Forage Processing.jpg]

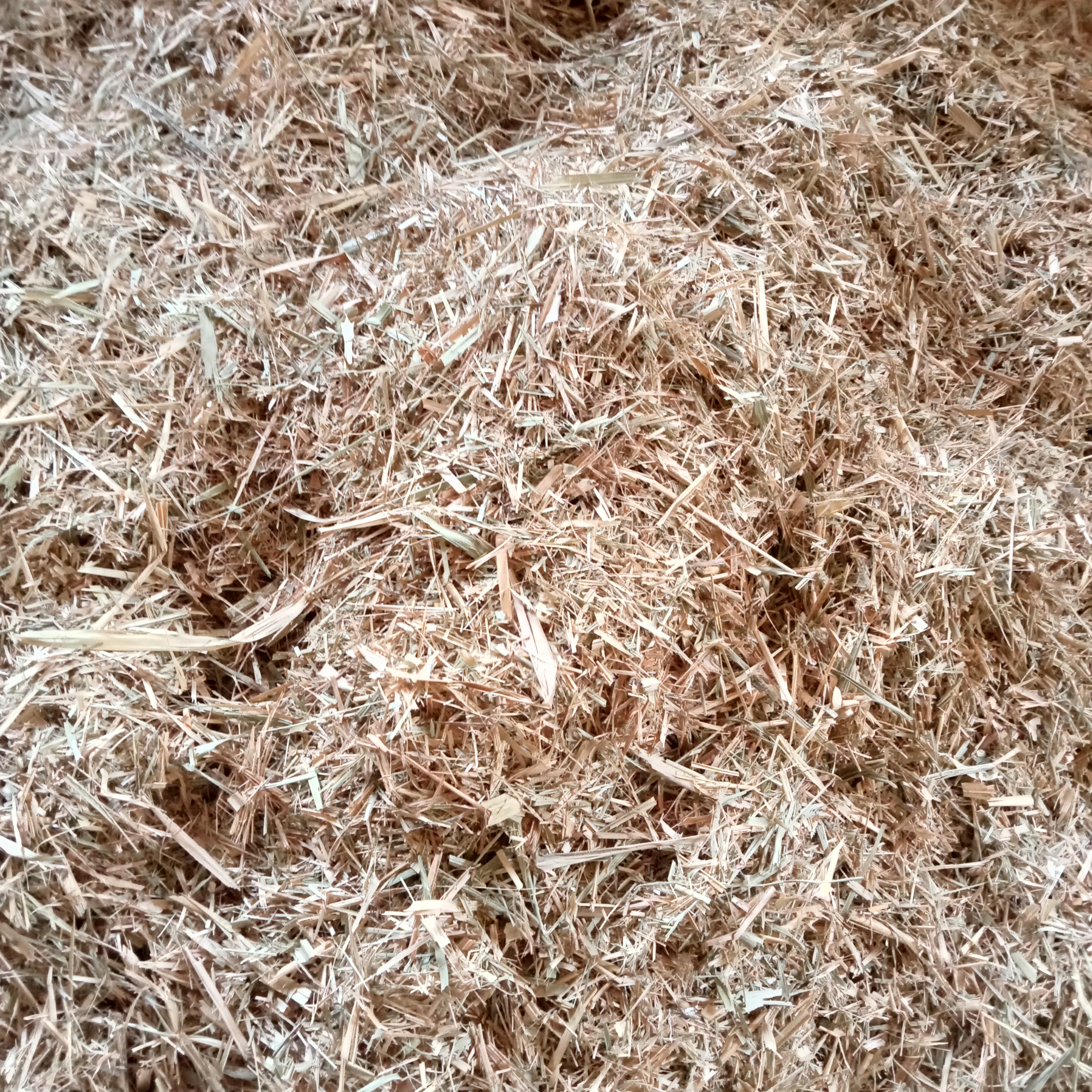

Supplement: Supplementary file 1 — Supplementary Material 1 [file 41598_2025_28525_MOESM1_ESM.zip › Meteab_Supplementary/photos/Panicum Forage Processing2.jpg]

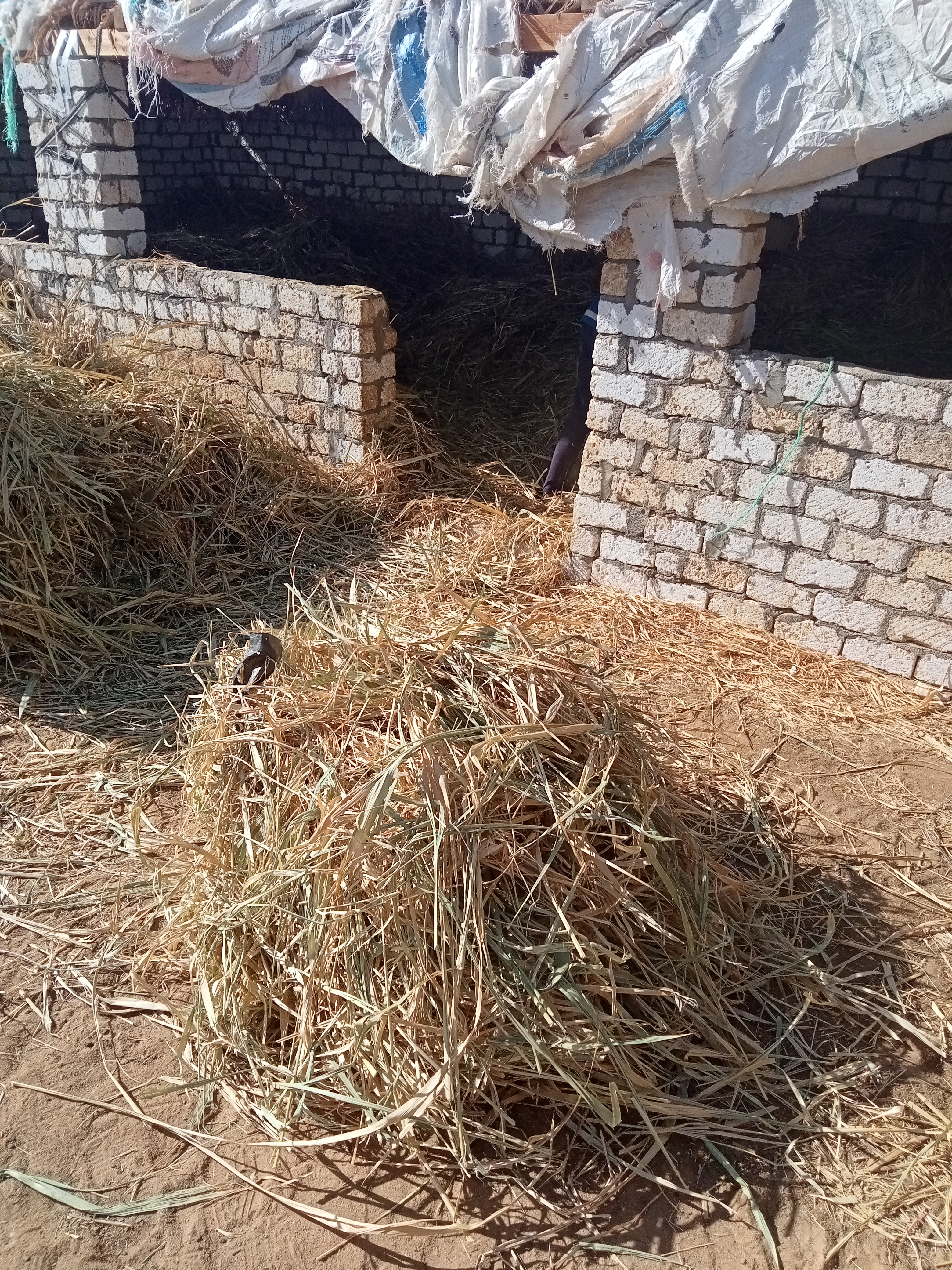

Supplement: Supplementary file 1 — Supplementary Material 1 [file 41598_2025_28525_MOESM1_ESM.zip › Meteab_Supplementary/photos/Panicum hay.jpg]

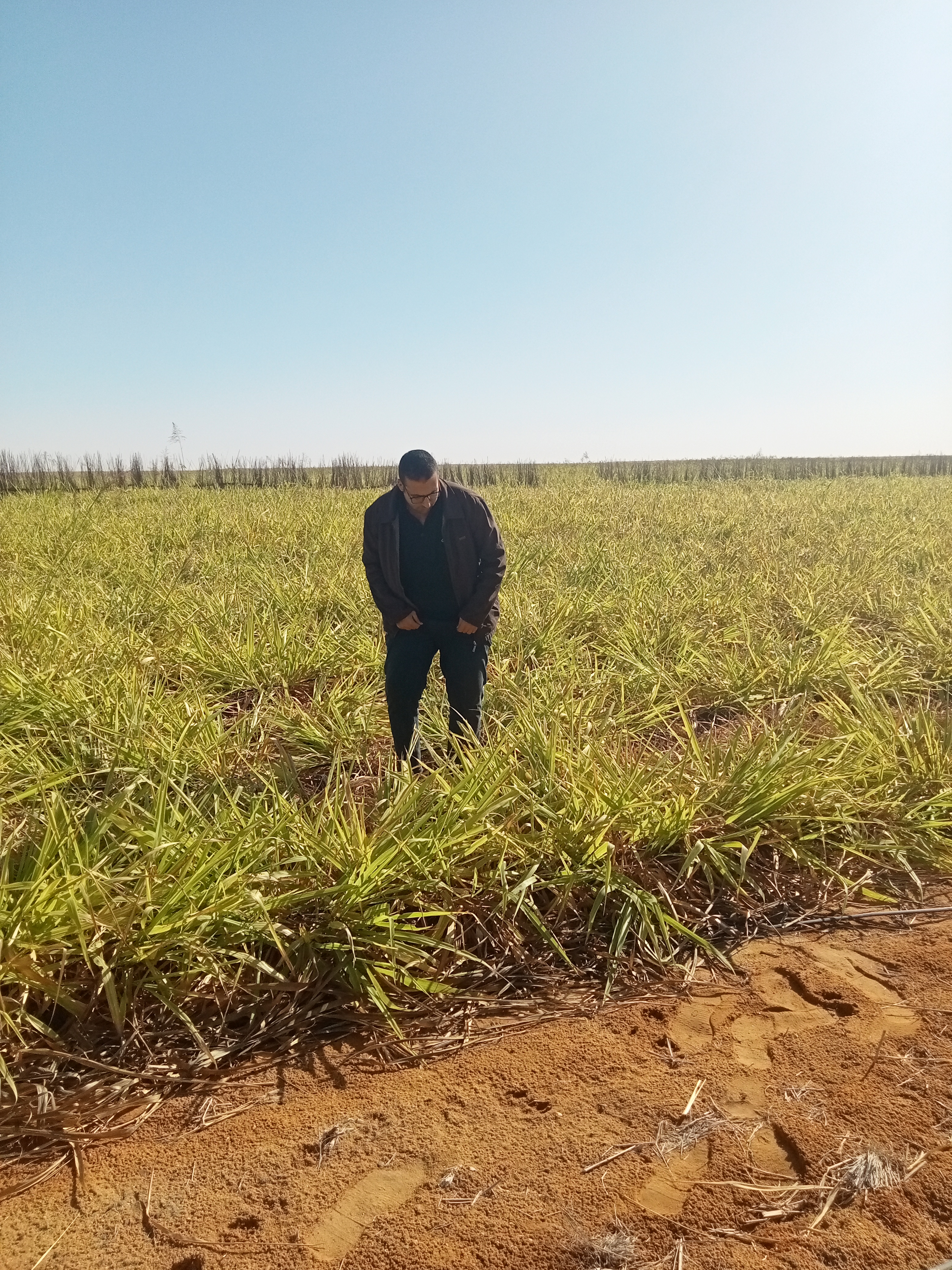

Supplement: Supplementary file 1 — Supplementary Material 1 [file 41598_2025_28525_MOESM1_ESM.zip › Meteab_Supplementary/photos/Panicum in Al-Maghrah 2.jpg]

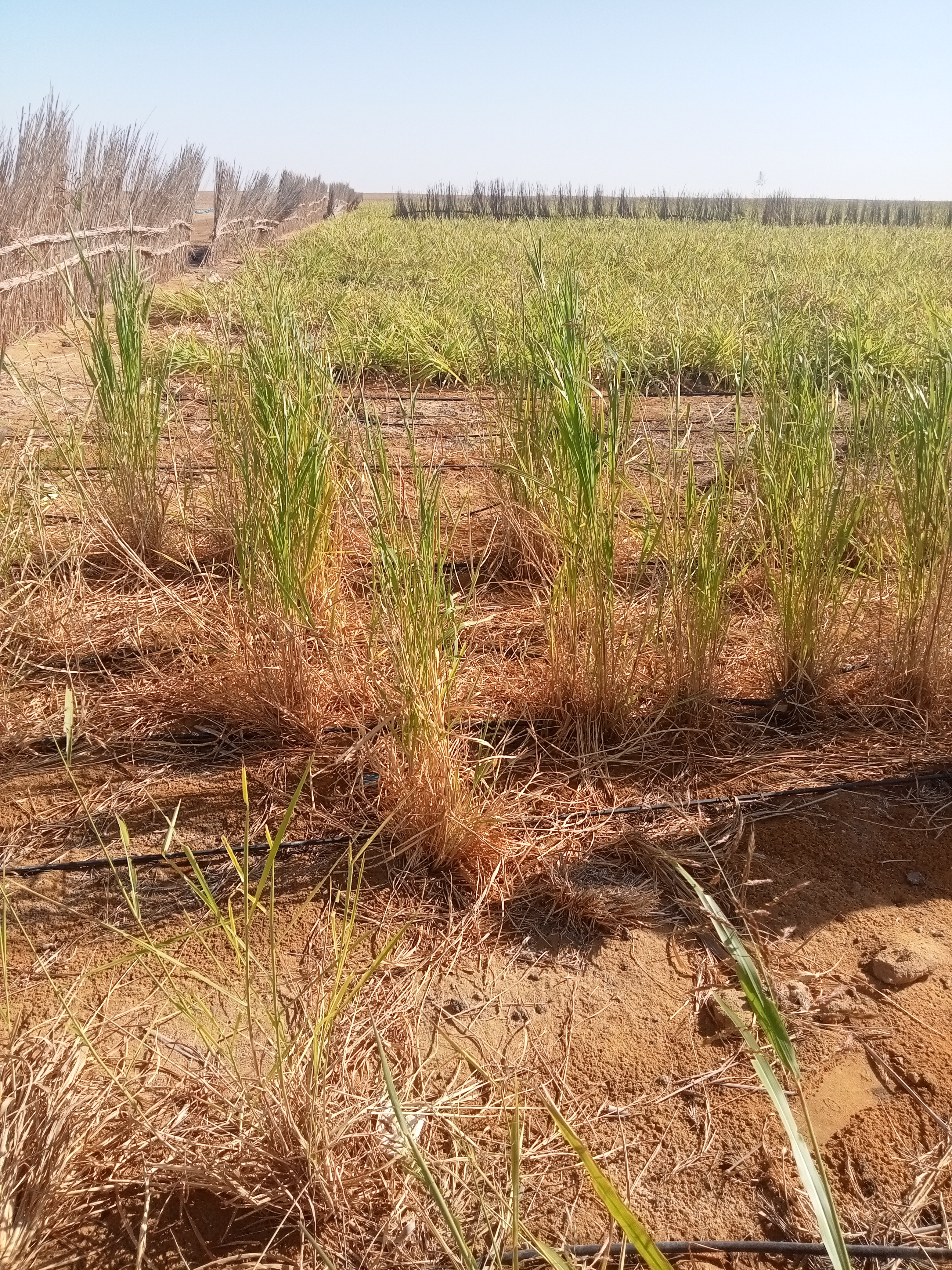

Supplement: Supplementary file 1 — Supplementary Material 1 [file 41598_2025_28525_MOESM1_ESM.zip › Meteab_Supplementary/photos/Panicum in Al-Maghrah.jpg]

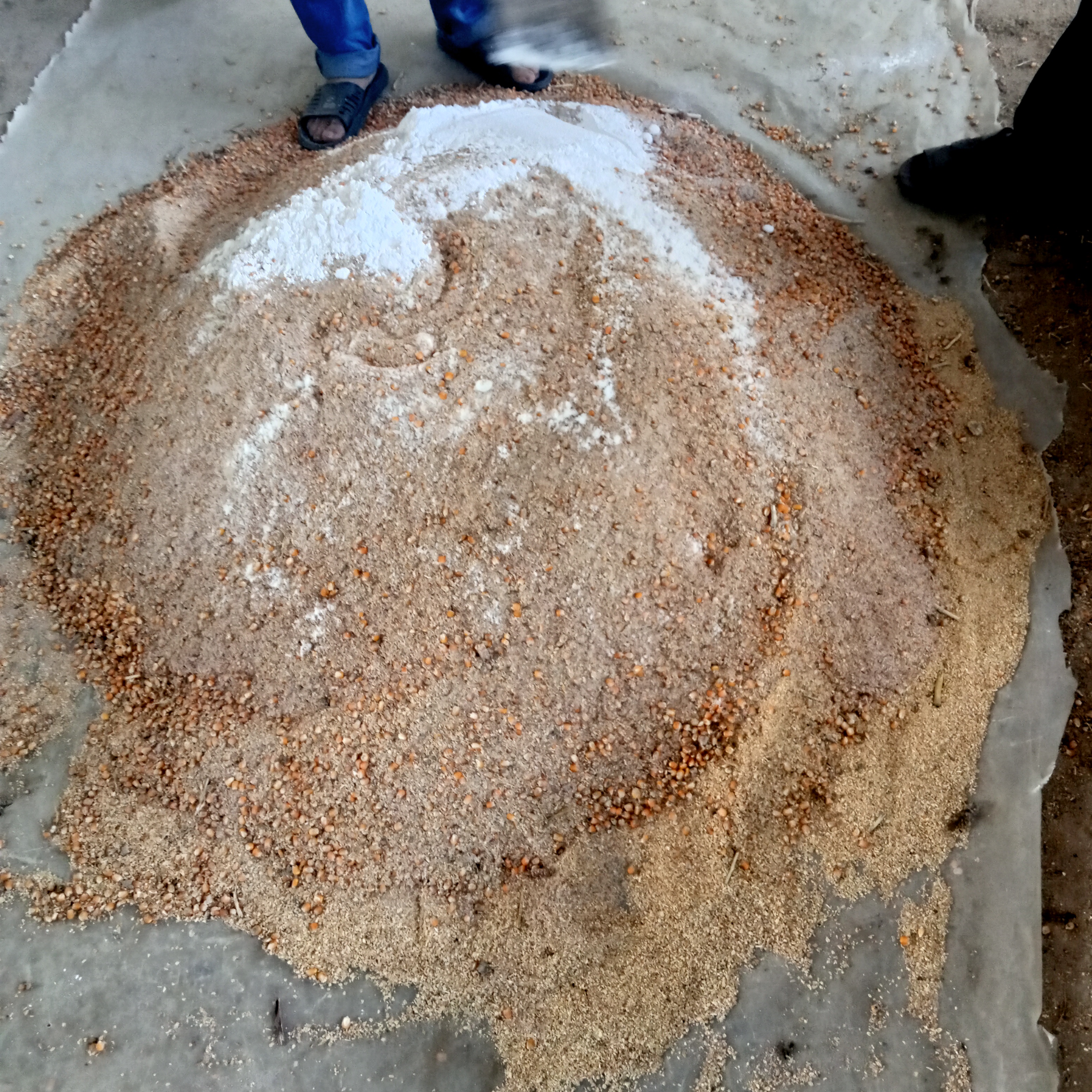

Supplement: Supplementary file 1 — Supplementary Material 1 [file 41598_2025_28525_MOESM1_ESM.zip › Meteab_Supplementary/photos/Ration Formulation.jpg]

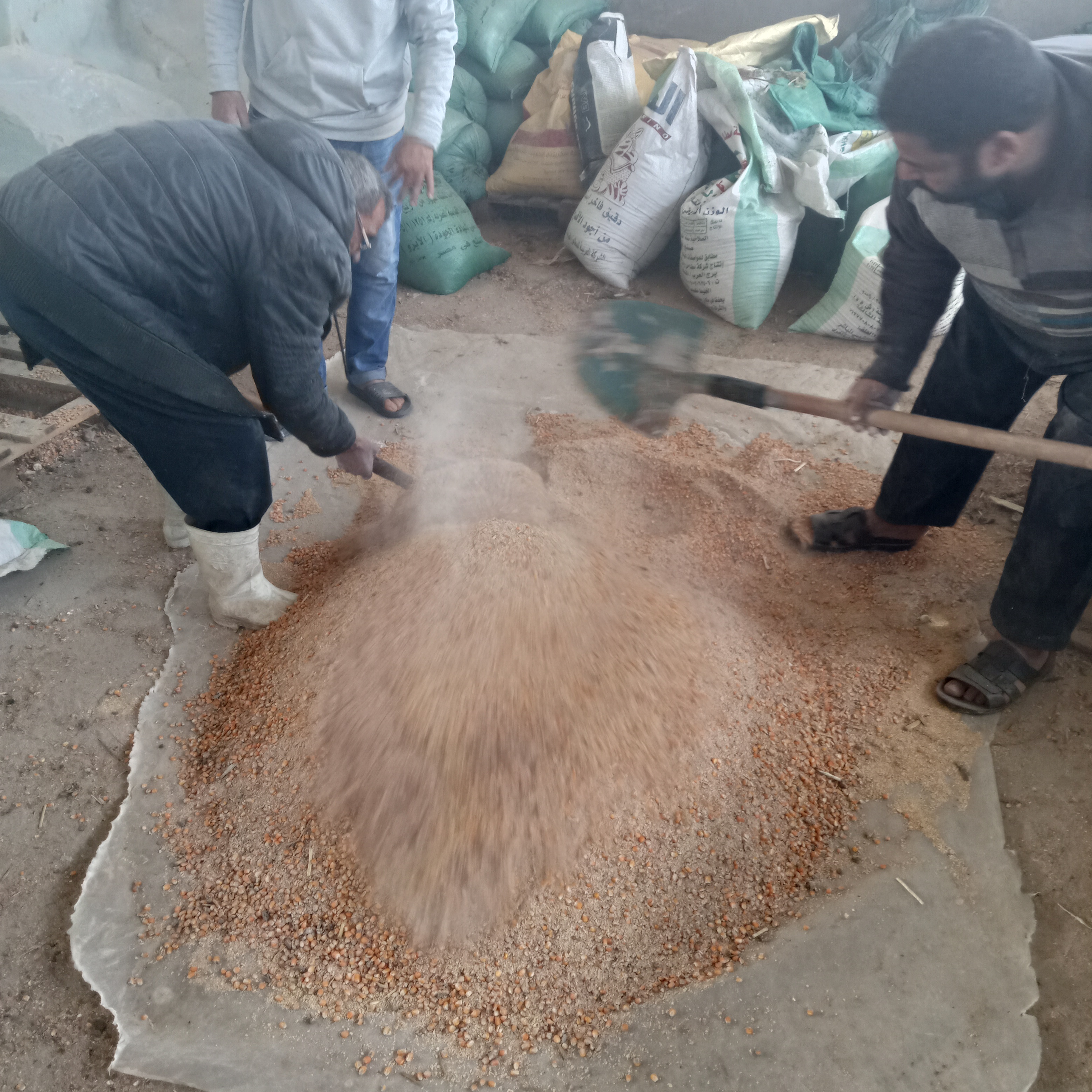

Supplement: Supplementary file 1 — Supplementary Material 1 [file 41598_2025_28525_MOESM1_ESM.zip › Meteab_Supplementary/photos/Ration Formulation2.jpg]

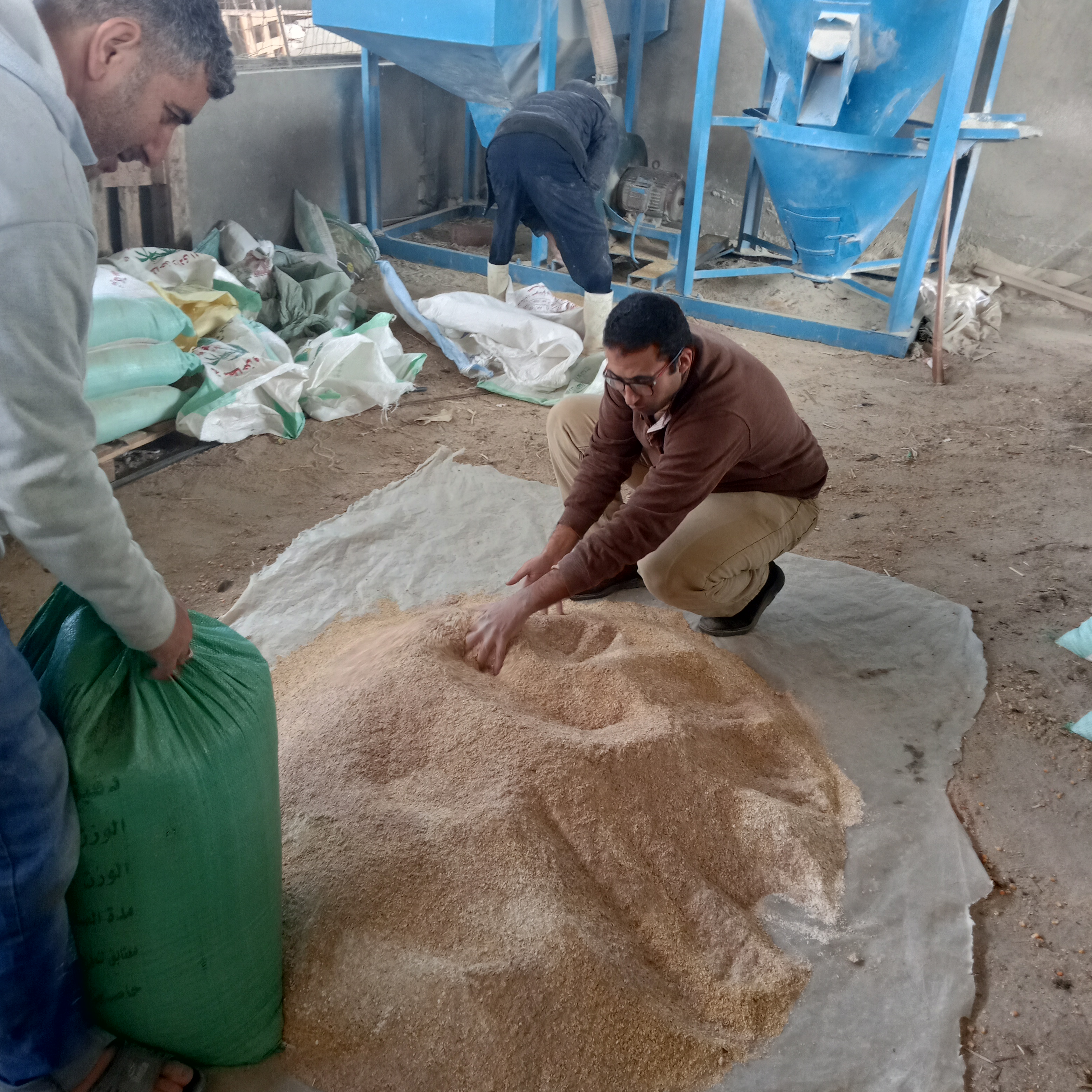

Supplement: Supplementary file 1 — Supplementary Material 1 [file 41598_2025_28525_MOESM1_ESM.zip › Meteab_Supplementary/photos/Ration Formulation3.jpg]

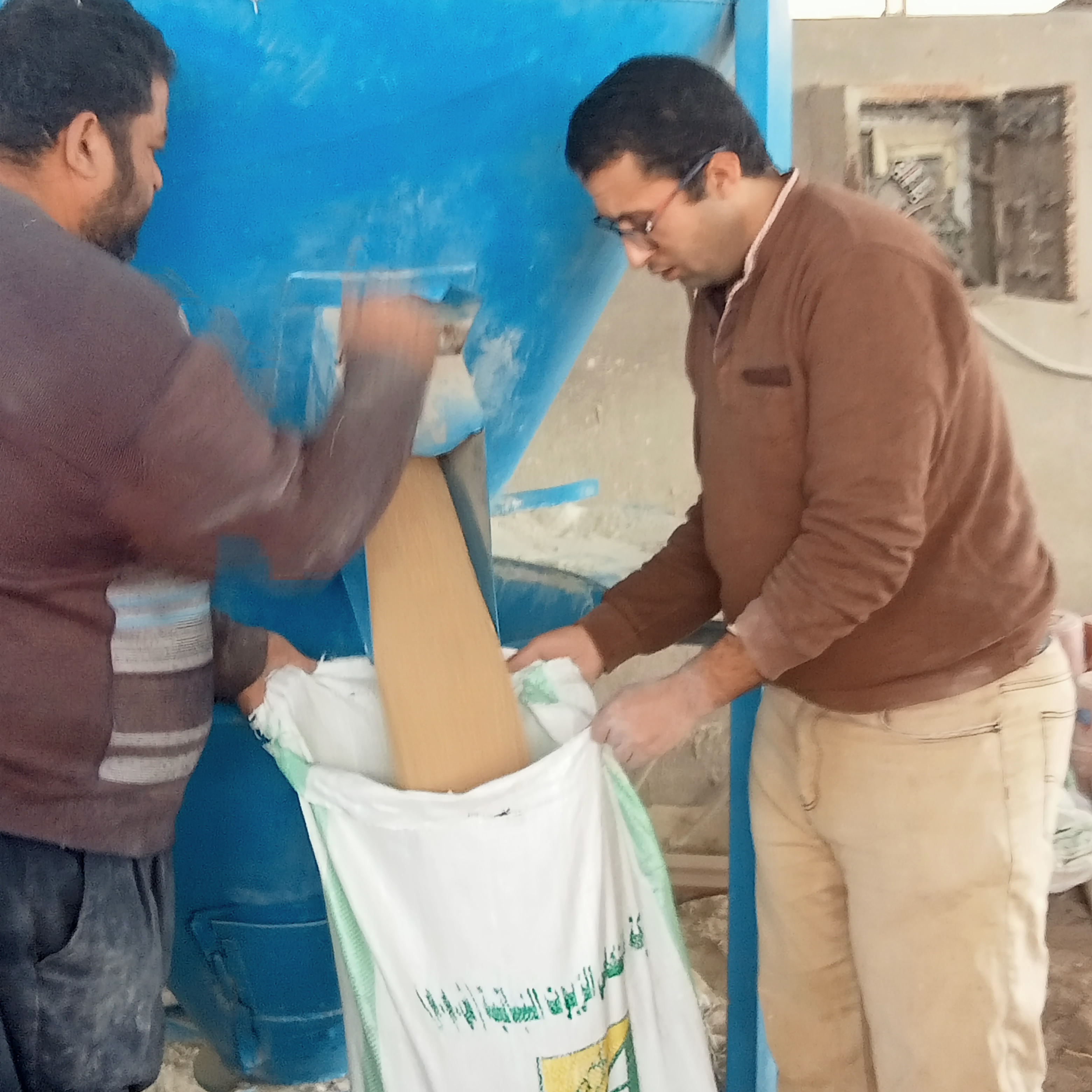

Supplement: Supplementary file 1 — Supplementary Material 1 [file 41598_2025_28525_MOESM1_ESM.zip › Meteab_Supplementary/photos/Ration Formulation4.jpg]

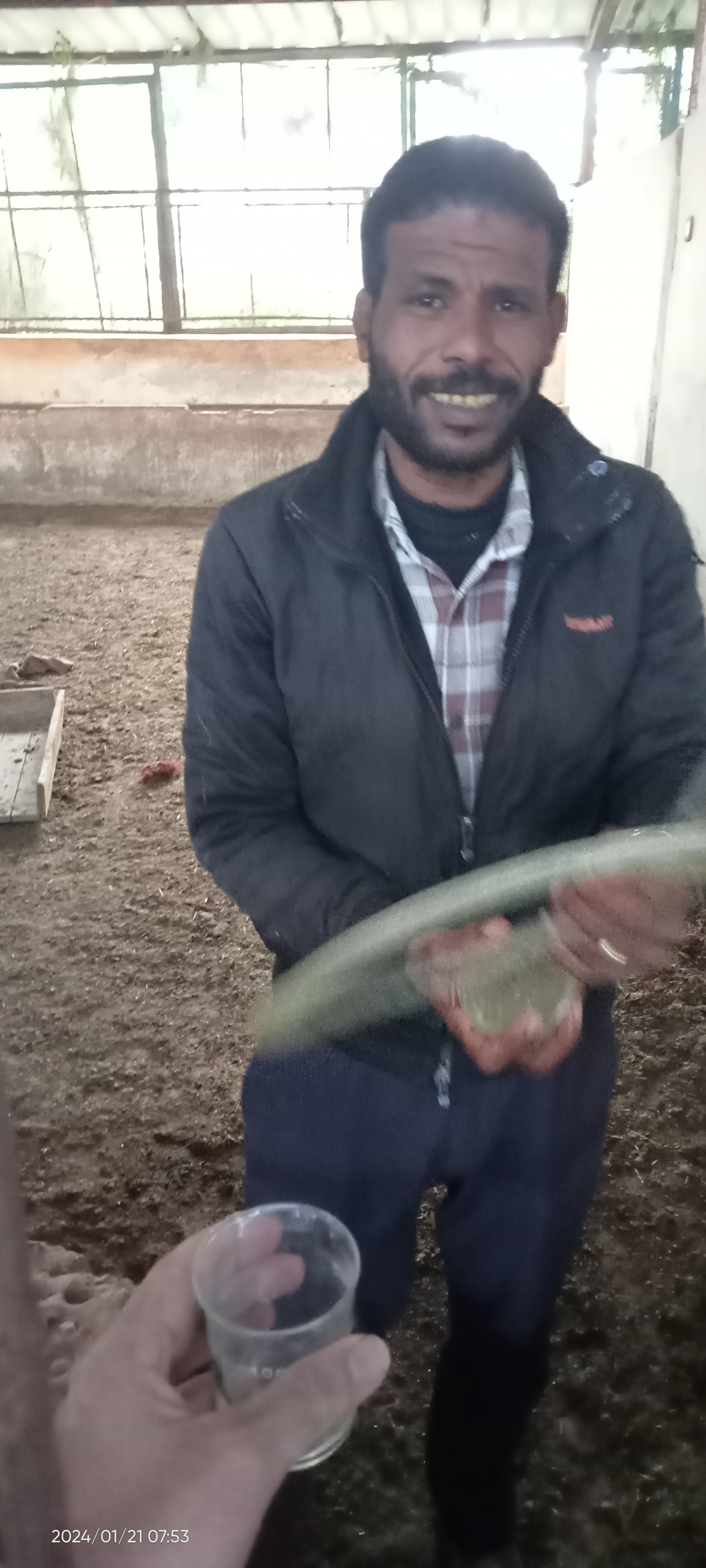

Supplement: Supplementary file 1 — Supplementary Material 1 [file 41598_2025_28525_MOESM1_ESM.zip › Meteab_Supplementary/photos/samples of rumen liquor 2.jpg]

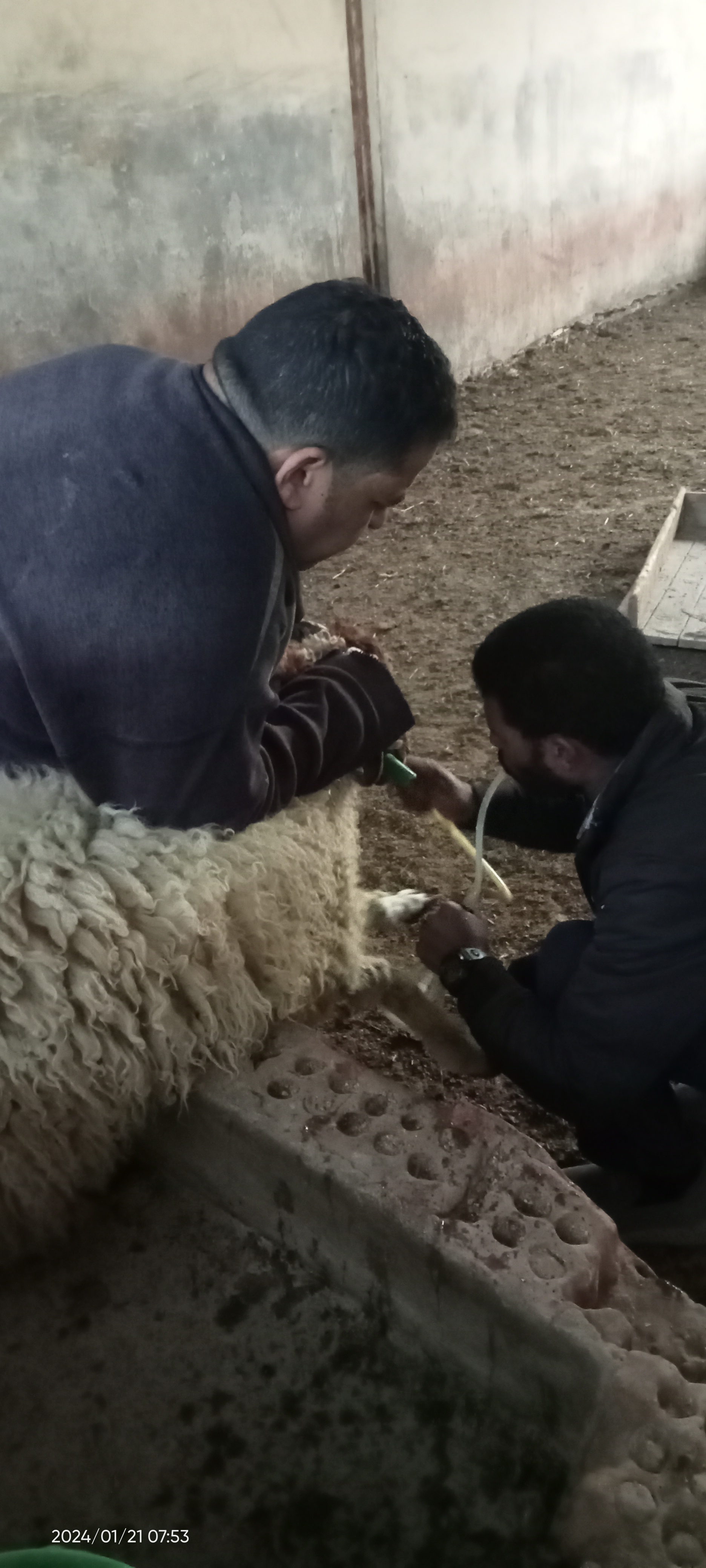

Supplement: Supplementary file 1 — Supplementary Material 1 [file 41598_2025_28525_MOESM1_ESM.zip › Meteab_Supplementary/photos/samples of rumen liquor.jpg]
